# Obese mice induced by high-fat diet have differential expression of circular RNAs involved in endoplasmic reticulum stress and neuronal synaptic plasticity of hippocampus leading to obesity-associated cognitive impairment

**DOI:** 10.3389/fnmol.2022.1000482

**Published:** 2022-10-03

**Authors:** Yan Niu, Pan Chang, Tian Liu, Xi Shen, Hui Zhao, Mingxia Zhang, Shengping Lei, Baoying Chen, Jun Yu

**Affiliations:** ^1^Clinical Experimental Center, Xi'an International Medical Center Hospital, Xi'an, China; ^2^Department of Cardiology, The Second Affiliated Hospital, Xi'an Medical University, Xi'an, China; ^3^Imaging Diagnosis and Treatment Center, Xi'an International Medical Center Hospital, Xi'an, China

**Keywords:** high-fat diet-induced obesity, circRNAs, obesity-associated cognitive impairment, endoplasmic reticulum stress, neuronal synaptic plasticity

## Abstract

Obesity induced by a high-fat diet (HFD) is an important cause of impaired memory and cognitive function, but the underlying mechanisms are not clear. In the present study, we analyzed the levels of circRNAs in the hippocampus of C57BL/6J mice and evaluated the memory and cognition ability of C57BL/6J mice with HFD using Morris water maze and Y-maze approaches to explore the potential mechanisms linking circRNAs in obesity-associated cognitive impairment. Learning performance showed that HFD-induced obesity mice have impaired memory and cognition. The Arraystar analysis of the hippocampus displayed that HFD-induced obesity leads to the differential expression of circRNAs (DE-circRNAs) in mice. In total, 46 circular RNAs with elevated expression and 10 with decreased expression were identified. Among them, mmu_circRNA_004797 was identified to be significantly downregulated and the expression of mmu_circRNA_21040 was significantly upregulated in the HFD-fed mice, compared with control mice by PCR test. Bioinformatics analysis also showed that the upregulated circRNAs were related to the neuronal function and behavior, and material transport process, while downregulated circRNAs participated in the process of cell response to external stimuli, such as cellular response to nutrient levels. Furthermore, the KEGG pathway analysis showed that the upregulated circRNAs are mainly involved in Axon guidance, calcium signaling pathway, and ErbB signaling pathway. Only a single significant pathway, that is, “protein processing in endoplasmic reticulum”, was observed in the downregulated circRNAs. Finally, we examined the deficits of hippocampal synaptic plasticity and detected the expression of ER stress-related protein. The results showed that ER stress was activated in the hippocampus, and hippocampal synaptic plasticity deficits were displayed. Our results demonstrated that circRNAs were most likely implicated in the predisposition to obesity-associated cognitive impairment.

## Introduction

Obesity has become a pandemic in recent decades, having been considered to be undoubtedly one of the greatest public health challenges around the world. Obesity is defined as a complex chronic disease, caused by an imbalance between food intake and energy expenditure (Pan et al., 2021). Obesity is a contributor to many adverse health outcomes, including increased risk for dementia and adverse structural and functional changes to multiple organs (Vecchié et al., 2018; Zhang W. et al., 2019). The high prevalence of obesity is one of the leading causes of elevated cognitive dysfunction. It has been reported that obesity has many deleterious effects on the brain, including changes in endoplasmic reticulum stress, autophagy, oxidative stress, and inflammation reaction that are believed to promote neurological disease (Alzoubi et al., 2018; Kong et al., 2018; Tan et al., 2018; Ye et al., 2018; Tan and Norhaizan, 2019). Moreover, accumulating evidence indicates that obesity is a potential risk factor for many metabolic syndromes, including glucose intolerance, insulin resistance, hyperinsulinemia, dyslipidemia, nonalcoholic fatty liver disease, and type 2 diabetes (Polyzos et al., 2019; Piché et al., 2020) These are closely related to the occurrence of cognitive dysfunction. Thus, it is noteworthy that obesity-associated cognitive impairment has become increasingly serious, and may be an early manifestation of neurodegenerative diseases, which should be given high attention. Our study aimed to elucidate the pathogenesis that predisposes to cognitive impairment after obesity.

Circular RNAs are defined as a kind of endogenous non-coding RNAs (ncRNAs) which are a group of RNAs generated during splicing with a covalently closed cyclic structure (Kristensen et al., 2019). An important feature of circRNA is its “microRNA (miRNA) sponge” function, which can effectively bind and inhibit miRNA activity, further affecting the downstream mRNA expression and ultimately participating in various diseases (Patop et al., 2019); that is, circRNA can pass the competitive endogenous RNA (ceRNA) network and act as a microRNA sponge to inhibit the function of microRNAs. Therefore, it is very important to identify the circRNA-miRNA-mRNA network in the process of disease occurrence and development, and as a new form of post-transcriptional regulation, this network has great potential significance in disease research. Accumulating evidence indicates that circRNA plays a critical role in many diseases, including nervous system disorders, cardiovascular diseases (CVDs), diabetes, and cancer (Mehta et al., 2020; Chen and Shan, 2021; Sakshi et al., 2021; Zhu et al., 2021). Recent research indicates that circRNAs are highly enriched in mammalian brain tissue (Mehta et al., 2020). As the nervous system ages, circRNAs are significantly expressed in age-related brain tissues and have been identified as an indicator of the aging process (Lo et al., 2020). Additionally, some of the conserved circRNAs have been identified to be derived from synaptic genes and are regulated depending on the developmental stage (Hanan et al., 2020). Thus, it is important to clarify the role that circRNAs play in obesity-associated cognitive impairment. Given that the hippocampus is a brain region linked to cognition, we analyzed the expression patterns with Arraystar Mouse circRNA Array analysis of hippocampus tissues of mice exposed to HFD for 16 weeks in the study. The present study contributes to the understanding of the potential mechanisms of circRNA to obesity-associated cognitive dysfunction.

In addition, it has been reported that endoplasmic reticulum stress plays a key role in cognition, and it has been shown that endoplasmic reticulum stress is activated in HFD-fed mice (Zhu, 2020). Neuronal synaptic plasticity is the foundation for the neurons to exert their function (Wiera et al., 2021). In this study, we also detected the expression of related proteins to fully clarify the pathogenesis of obesity-associated cognitive impairment. Therefore, we set out to determine the role of circRNA, endoplasmic reticulum stress, and hippocampal synaptic plasticity in the process of obesity-associated cognitive dysfunction.

## Materials and methods

### Animals and diets

C57BL/6J mice aged 6 weeks (male, body weight: 18–22 g; *n* = 15 for each group) were obtained from the Laboratory Animal Center of Xi'an Jiaotong University (Shaanxi, China) and housed in a clean environment at the Laboratory of Clinical Experimental Center, Xi'an International Medical Center Hospital (Shaanxi, China) with standard conditions (room temperature = 23 ± 2°C; humidity = 45 ± 5%), and 12 h/12 h light-dark cycle (light on at 8:30 a.m.). All animal experimental protocols involved were performed in strict accordance with the Guide for the Care and Use of Laboratory Animals and approved by the Institutional Animal Care and Use Committee of Xi'an International Medical Center Hospital. All mice had access to a standard chow and tap water. After adaptive feeding for 1 week, the mice were randomly assigned to two groups as follows: (1) STD (male mice, given a normal diet, *n* = 12); (2) HFD (male mice, given a high-fat diet, *n* = 12). The normal diet contained 10 kcal% fat, 20 kcal% protein, and 70 kcal% carbohydrates, while the high-fat diet (HFD, D12492, Research Diets, Inc.) contained 60 kcal% fat, 20 kcal% protein, and 20 kcal% carbohydrates.

### Body weight and blood glucose levels

During the feeding period, the body weight of mice was tested at a fixed time every other week and blood glucose levels were determined with a tail nick and Accu-Chek glucose meter (Roche Diagnostics, Germany).

### Oral glucose tolerance test and insulin resistance test

An oral glucose tolerance test was conducted at 16 weeks after feeding HFD. Briefly, after a 12-h fasting, tail-vein blood was collected and measured with an Accu-Chek glucose meter (Roche Diagnostics, Germany) at 0, 15, 30, 60, 90, and 120 min after glucose treatment (orally administrated 2 g/kg of glucose dissolved in water). ITT was carried out for 3 days after OGTT to ensure recovery.

### Spatial learning memory test

#### Morris water maze

Morris water maze (MWM) test was used to evaluate the spatial learning and memory of mice. The whole experiment was divided into two parts. First, mice were given acquired training for 4 days, and then exploration training was carried out the next day after the training. The specific methods were as follows: (1) Acquired training: randomly choose a direction from the four starting positions of east, west, south, and north, and then put the mouse in the pool with the head toward the pool wall. Record the time (s) when the mouse finds the underwater platform; if the time exceeds 60 s, guide the animal to the platform and let the mouse stay on the platform for 10 s. After that, remove and dry the mouse and put it back into the cage. Each mouse was trained for 4 days. (2) Exploration training: on the second day after the last acquired training, remove the platform and start exploration training for 60 s. Place the mouse in the water from the opposite side of the original platform quadrant. The time spent in the target quadrant (the quadrant where the platform was originally placed) and the times of entering the quadrant were recorded as the detection index.

#### Y-maze

In the Y-maze test, we used a spontaneous alternation experiment and spatial recognition experiment to further evaluate the memory function of mice. (1) Spontaneous alternation experiment: In this experiment, the mouse is placed in the maze from the end of any arm and allowed to explore freely in it for 8 min, and the total number of times the mouse enters each arm is recorded (the animals have all four feet entering an arm is considered entering the arm once). An alternation is defined as the entry of the mouse into the three arms of the maze in sequence, such as 1, 2, 3 or 1, 3, 2. Record the total distance and the total number of arms entry that mouse moved in the maze. The maximum number of turns is the total number of arm advances-2, and the percentage of alternation = total number of turns/maximum number of turns × 100%. (2) In the spatial recognition test, there are two stages. The first stage is the learning period. In this period, one arm of the Y-maze is closed (the arm is defined as the novel arm), and the other two arms are opened. The mice are allowed to explore freely for 3 min. After an interval of 20 min, the next stage (recall stage) of the experiment was conducted. In this stage, the mice are allowed to explore freely for 5 min in the Y-maze with all arms opened, and the time and distance explored by the mice in the novel arm are recorded.

### Sample collection and preparation

After 16 weeks of HFD feeding, all mice were euthanized, and hippocampus samples were isolated immediately after mice were sacrificed, snap-frozen in liquid nitrogen, and then stored at −80°C until analysis.

### Histopathological examination

Brain tissue was fixed in 4% formalin before being embedded in paraffin. Serial sections of the embedded specimens were stained with hematoxylin, eosin, and toluidine blue with the standard protocol. The histopathological changes in hippocampus tissues were randomly assessed.

### Arraystar mouse circRNA array analysis

#### Experiment workflow

First, total RNA from the samples of both groups was extracted from hippocampus tissues, and the concentrations of the RNA samples were determined by measuring the absorbance at OD260 using a NanoDrop ND-1000 instrument. The integrity of RNA was assessed by electrophoresis on a denaturing agarose gel. Then, sample labeling and array hybridization were performed according to the manufacturer's protocol (Arraystar Inc.) as follows. (1) RNA labeling: total RNAs were digested with Rnase R (Epicentre, Inc.) to remove linear RNAs and enrich circular RNAs. Then, the enriched circular RNAs were amplified and transcribed into fluorescent cRNA utilizing a random priming method (Arraystar Super RNA Labeling Kit; Arraystar). The labeled cRNAs were purified by RNeasy Mini Kit (Qiagen). The concentration and specific activity of the labeled cRNAs (pmol Cy3/μg cRNA) were measured by NanoDrop ND-1000. (2) Array hybridization: 1 μg of each labeled cRNA was fragmented by adding 5 μl of 10 × blocking agent and 1 μl of 25 × fragmentation buffer, then heated the mixture at 60°C for 30 min, and finally, 25 μl of 2 × hybridization buffer was added to dilute the labeled cRNA. About 50 μl of hybridization solution was dispensed into the gasket slide and assembled to the circRNA expression microarray slide. (3) Array scanning: after washing, the slides were incubated for 17 h at 65°C in an Agilent Hybridization Oven. The hybridized arrays were washed, fixed, and scanned using the Agilent Scanner G2505C. (4) Data collection and analysis: Scanned images were imported into Agilent Feature Extraction software for raw data extraction.

#### Flowchart of data analysis

(1) Raw data extraction: data were extracted using Agilent Feature Extraction software. (2) Expression Profiles of circRNAs: A series of data processing steps, including quantile normalization, were performed using the R software limma package. The circRNAs that have at least 1 out of 10 samples have flags in “p” or “M” (“All Targets Value”, defined by GeneSpring software) were retained for further differential analyses. (3) Differentially expressed circRNAs: differentially expressed circRNAs with statistical significance between two samples or two groups were identified using fold change cutoff or through volcano plot filtering, respectively. (4) Annotation for circRNA/microRNA interaction: the circRNA/microRNA interaction was predicted with Arraystar's home-made miRNA target prediction software. All the differentially expressed circRNAs were annotated in detail with the circRNA miRNA interaction information. When comparing two groups of profile differences (such as disease vs. control), the “fold change” (i.e., the ratio of the group averages) between the groups for each circRNA is computed. The statistical significance of the difference may be conveniently estimated by a *t*-test.

#### Gene ontology enrichment

The GO project is a major bioinformatics initiative to unify the representation of gene and gene product attributes across all species. It provides an ontology of defined terms representing gene product properties. To probe the main biological process (BPs), cellular components (CCs), and molecular functions (MFs) of differentially abundant proteins, the circRNAs were further summarized based on GO terms.

#### KEGG enrichment

The KEGG database is a collection of manually drawn KEGG pathway maps representing experimental knowledge on metabolism and various other functions of the cell and organism. Furthermore, the KEGG map module was employed to display the enzymatic functions of the detected proteins from the perspective of the metabolic pathways in which they participate. In this study, we analyzed differentially abundant proteins associated with metabolic pathways to elucidate the effects of HFD on the cognitive phenotype of mice by KEGG enrichment.

### Prediction of DE-circRNA-miRNA-mRNA network

According to the CeRNA hypothesis, RNA transcripts can crosstalk by competing for common microRNAs, with microRNA response elements (MREs) as the foundation of this interaction (Salmena et al., 2011). These RNA transcripts have been termed as competing endogenous RNAs-ceRNAs (Phelps et al., 2016). Any RNA transcript with MREs might act as ceRNA, and ceRNAs include pseudogene transcripts, lncRNAs, circRNAs, and mRNAs; these transcripts can compete for the same microRNA response elements (MERs) to regulate mutually. To find the potential target of microRNAs, the target/microRNAs are predicted with home-made miRNA target prediction software based on TargetScan & miRanda (Enright et al., 2003; Friedman et al., 2009). Through merging the commonly targeted miRNAs, we constructed the ceRNA network. Three conditions must exist for the ceRNA network to occur (Salmena et al., 2011). First, the relative concentration of the ceRNAs and their microRNAs is clearly important; second, the effectiveness of a ceRNA would depend on the number of microRNAs that it can “sponge”; third, not all of the MREs on ceRNAs are equal. So, we only accept these ceRNA-pair relations passing some measures filtering with the threshold value of p-value calculated by hypergeometric test. The pvalue should be smaller than this threshold value (default value is 0.05, the smaller the better).

### Quantitative real-time PCR

To confirm the expression of differentially expressed circRNAs (DE-circRNAs), total RNA was extracted from the hippocampus by using TRIzol (Invitrogen). The cDNA was reverse-transcribed using SuperScriptTM III Reverse Transcriptase (Invitrogen). Briefly, the mixture contained RNA, Random (N9), dNTPs Mix, and RNase-free dH2O, and was placed in a 65°C water bath for 5 min and on ice for 2 min. After brief centrifugation, RT reaction solution with 5X First-Strand Buffer, DTT, RNase inhibitor, and SuperScript III RT was added. After mixing, the temperature was kept at 37°C for 1 min, and then incubated at 50°C for 60 min and 70°C for 15 min to inactivate the enzyme. Finally, synthesized cDNA was stored at −20°C for real-time quantitative PCR. Then, real-time quantitative PCR was performed using a 2X PCR master mix (Arraystar) and ViiA 7 Real-time PCR System (Applied Biosystems). The qPCR reaction system included 2 μl of cDNA, 5 μl of 2C Master Mix (Arraystar, Inc.), and 0.5 μl of each primer (10 μM solution), and diethylpyrocarbonate-treated water was added to obtain a total volume of 10 μl. The thermocycling conditions consisted of an initial denaturation at 94°C for 3 min, followed by 40 cycles of denaturation at 94°C for 10 s, annealing at 56°C for 20 s, extension at 72°C for 30 s, and a final extension at 72°C for 5 min. All qPCR reactions were performed in triplicate. The GAPDH were used as internal controls, and the data were analyzed using the comparative Cq (ΔΔCq) method. All primer sequences are presented in Table 1.

**Table 1:** Quantitative real-time PCR primers used to confirm the expression of circRNAs.

**Target genes**	**Primer sequences**	**Annealing temperature, (°C)**	**Product length, (bp)**
GAPDH	F:5′ CACTGAGCAAGAGAGGCCCTAT3′	60	124
	R:5′ GCAGCGAACTTTATTGATGGTATT3′		
mmu_circRNA_004797	F:5′ CCATTGGCAACGAGTTGAAC 3′	60	58
	R:5′ TCGGTCTTCCAGCCTTGTCT 3′		
mmu_circRNA_19700	F:5′ CTACTGCTGAAGCCATAGGTT 3′	60	122
	R:5′ TATCGTGGAGAGAAGCTGAAA 3′		
mmu_circRNA_21040	F:5′ TGCTCTGTTCTTCATCCTTGC 3′	60	200
	R:5′ ACATTCCTCATCCCTTCCCA 3′		
mmu_circRNA_28712	F:5′ AAGAGCGATTCCTGAAACCTG 3′	60	97
	R:5′ CTCTGCATAGTCATCTGTCTCGG 3′		
mmu_circRNA_36123	F:5′ AGGAGACTGTAGACTGCTTGAAGA 3′	60	125
	R:5′ GGTGTCACTGTATCCCATTCTG 3′		
mmu_circRNA_37501	F:5′ TCGGGGAGATCAACACAAGTG 3′	60	168
	R:5′ CTTTCTTCCCCTTCTTCATCCA 3′		

### Western blot

Hippocampus tissues from mice were dissected and washed with ice-cold PBS and homogenized with RIPA buffer with 150 mmol/L NaCl as described in our previous study. Then, the tissue suspension was centrifuged at 4°C and 20,000 g for 30 min. After adjusting the protein concentration, the lysates were boiled in 5x SDS sample loading buffer for 5 min and separated by SDS-polyacrylamide gel electrophoresis. Gels were blotted on a polyvinylidene difluoride (PVDF) membrane (Immobilon P; Millipore, Bedford, MA, USA) and stained with the indicated primary antibodies: anti-CHOP, (1:1,000 dilution, M, CST, 2895s), anti-PERK(1:1,000 dilution, R, CST,3192S), anti-pPERK (1:1,000 dilution, R, CST 3179s), anti-EIFf2A (1:1,000 dilution, R, CST 9722s), anti-pEIFf2A (1:1,000 dilution, R, CST 9721s), anti-GRP78 (1:1,000 dilution, R, CST 3183s), anti-GAPDH (1:5,000, R, Protein tech), anti-Actin (1:5,000, R, Protein tech), and anti-Tubulin (1:5,000, M, Protein tech). Antibody binding was detected with horseradish peroxidase (HRP)-conjugated secondary antibodies followed by chemiluminescence detection (ECL Plus; Amersham Pharmacia, Uppsala, Sweden).

### Statistical analysis

Statistical analysis was performed using the R software limma package, SPSS software (version 13.0, SPSS), and Prism 6 (GraphPad Software). A total of five samples were included in each group. DE-circRNAs were obtained by comparing the HFD group and the control. The student's *t*-test was used to obtain *P*-values. The adjusted *P*-values of DE-circRNAs were obtained by multiple checking and correction based on the Benjamini and Hochberg method and RT-qPCR. The statistical significance was assessed with Student's *t*-test. The results were expressed as the mean ± SEM of three experimental repeats. *P* < 0.05 was considered to indicate a statistically significant difference. All statistical analysis was performed in SPSS software (version 13.0, SPSS).

## Results

### HFD-induced obesity in mice showed weight gain, glucose tolerance, and insulin resistance

**Figure 1:**
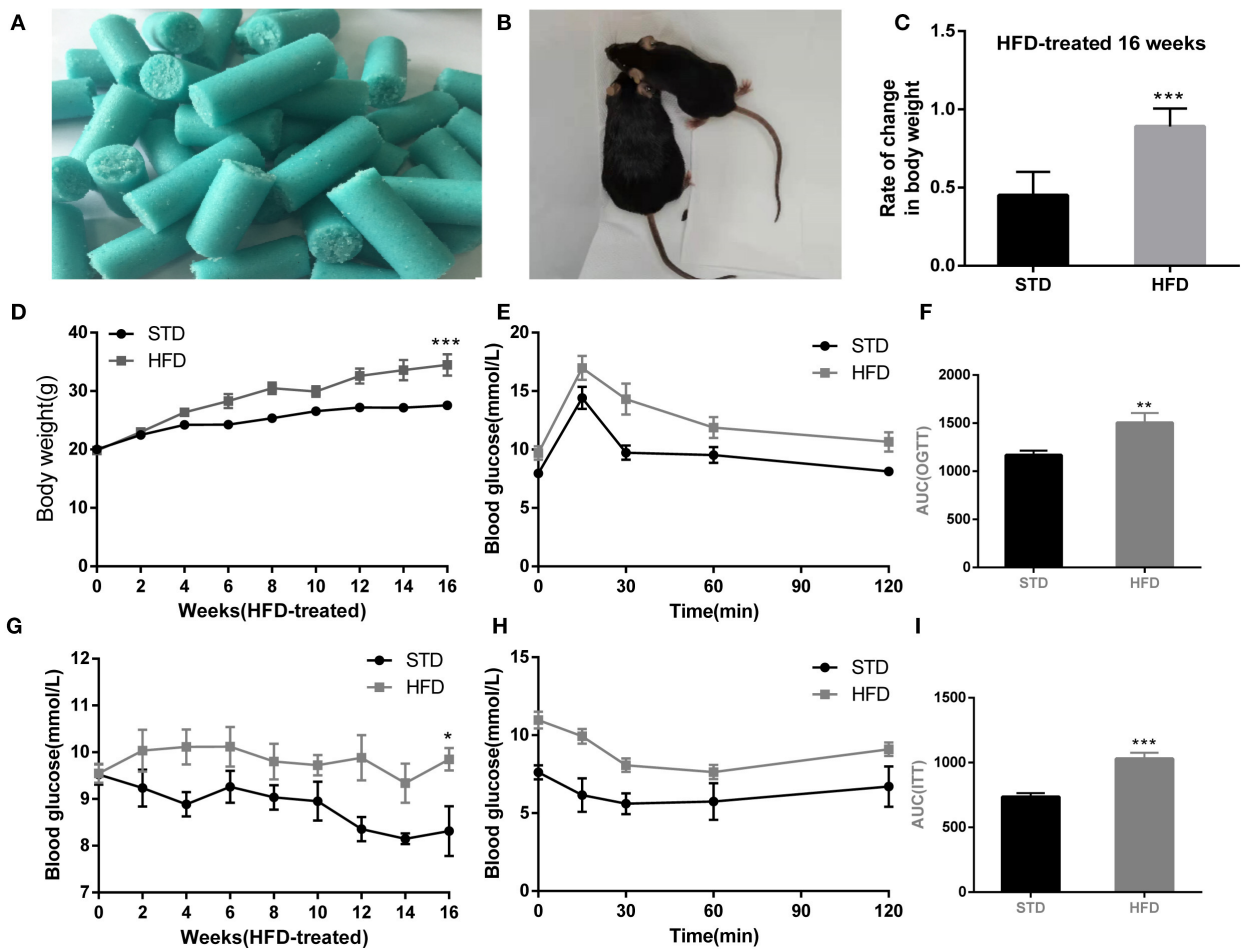
High-fat diet-induced obesity phenotype in mice. **(A)** High-fat diet D12492 from Research Diets was used in the study. **(B)** The picture of the mice treated by STD diet or HFD diet at 16 weeks. **(C)** Rate of change in body weight of the mice with HFD fed at 16 weeks. **(D)** The body weight of the mice during the HFD treatment was observed. **(E)** Levels of blood glucose during OGTT test. **(F)** Area under the curve from OGTT test. **(G)** Levels of random blood glucose of mice during feeding high-fat diet. **(H)** Levels of blood glucose during ITT test. **(I)** Area under the curve from ITT test. *P < 0.05; **P < 0.01;***P < 0.001. For the body weight and blood glucose test, during the monitoring of body weight and blood glucose, three measurements were made at each point, and the mean value was calculated as the data for final analysis (*n* = 12, 12).

Obesity is defined as a chronic disease resulting from abnormal or excessive fat accumulation due to a chronic imbalance between energy intake and expenditure and finally leading to nutritional and metabolic disturbances. Therefore, we used a high-fat diet (D12492, Research Diets, Inc. Figure 1A) in this study and constructed an obesity model in mice (Figure 1B). The final body weight of mice with 16 weeks of high-fat diet (HFD) feeding was substantially higher than that of standard diet (STD)-fed mice (34.47 ± 6.99 g vs. 27.55 ± 1.57 g in controls, *P* < 0.001; rate of change in body weight, 89%) (Figures 1C,D). In addition, the random blood glucose of mice increased remarkably in response to HFD (Figure 1G). To evaluate glucose tolerance in HFD mice, the oral glucose tolerance test (OGTT) was conducted at 16 weeks. The results showed the characteristic rapid rise of blood glucose in all mice, peaking within 15–30 min, and approaching the baseline level by 120 min after glucose challenge. However, the blood glucose level of HFD mice remained elevated after 120 min compared to other mice (Figures 1E,F), indicating that the HFD-fed mice were more likely to develop glucose tolerance. Insulin resistance test (ITT) results (Figures 1H,I) also showed that HFD mice were with lower insulin sensitivity and were easier to develop hyperinsulinemia than STD mice. Collectively, these results suggested that high-fat diets (HFD) represent a public health concern as they can predispose individuals to obesity.

### HFD-induced obesity is associated with cognitive impairment

**Figure 2:**
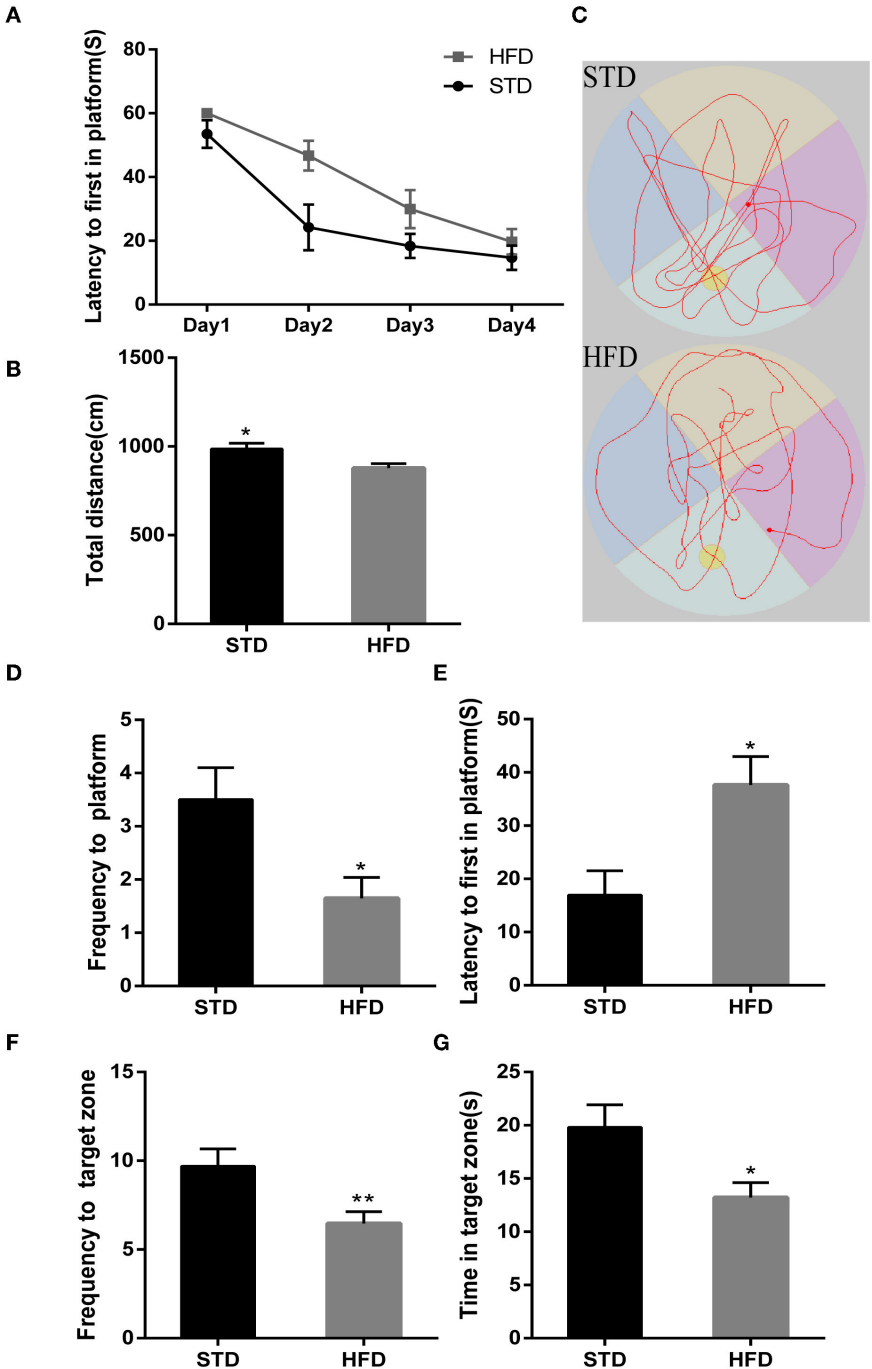
The results in mice during Morris water maze test. **(A)** The place navigation test. **(B)** The total swimming distance of mice during the test. **(C)** Swimming trajectory of mice during 1 min in the space probe test. **(D)** The frequency to a platform in the space probe test. **(E)** The escape latency of mice in the space probe test. **(F)** The frequency to the target zone where the platform is in during the test. **(G)** The target zone retention time during the test. *P < 0.05; **P < 0.01. In the place navigation test, mice were trained from the 4th quadrant of the maze, and four replicate experiments were performed for each mouse, *n* = 12, 12. In the space probe test, a fixed direction was selected to perform the test independently three times for each mouse, and mean values were calculated for final analysis, *n* = 12, 12.

To test cognitive performance, Morris water maze and Y-maze were used to observe the spatial memory and learning ability of mice in each group. Compared with the normal diet group, HFD mice have to take more time to escape to the platform in the place navigation test (Figure 2A). In the space probe test, HFD mice showed poor mobility, leading to a significant decrease in the total swimming distance (Figure 2B) during the test period when compared to that of the control mice. In addition, we observed that the frequency to the platform was reduced (Figures 2C,D), and the escape latency of HFD mice was significantly prolonged (Figure 2E). Meanwhile, Frequency to target zone where platform (Figure 2F) is in of HFD mice were significantly reduced, so as the target zone retention time reduced (Figure 2G). These data indicated that a high-fat diet leads to impaired cognitive function.

**Figure 3:**
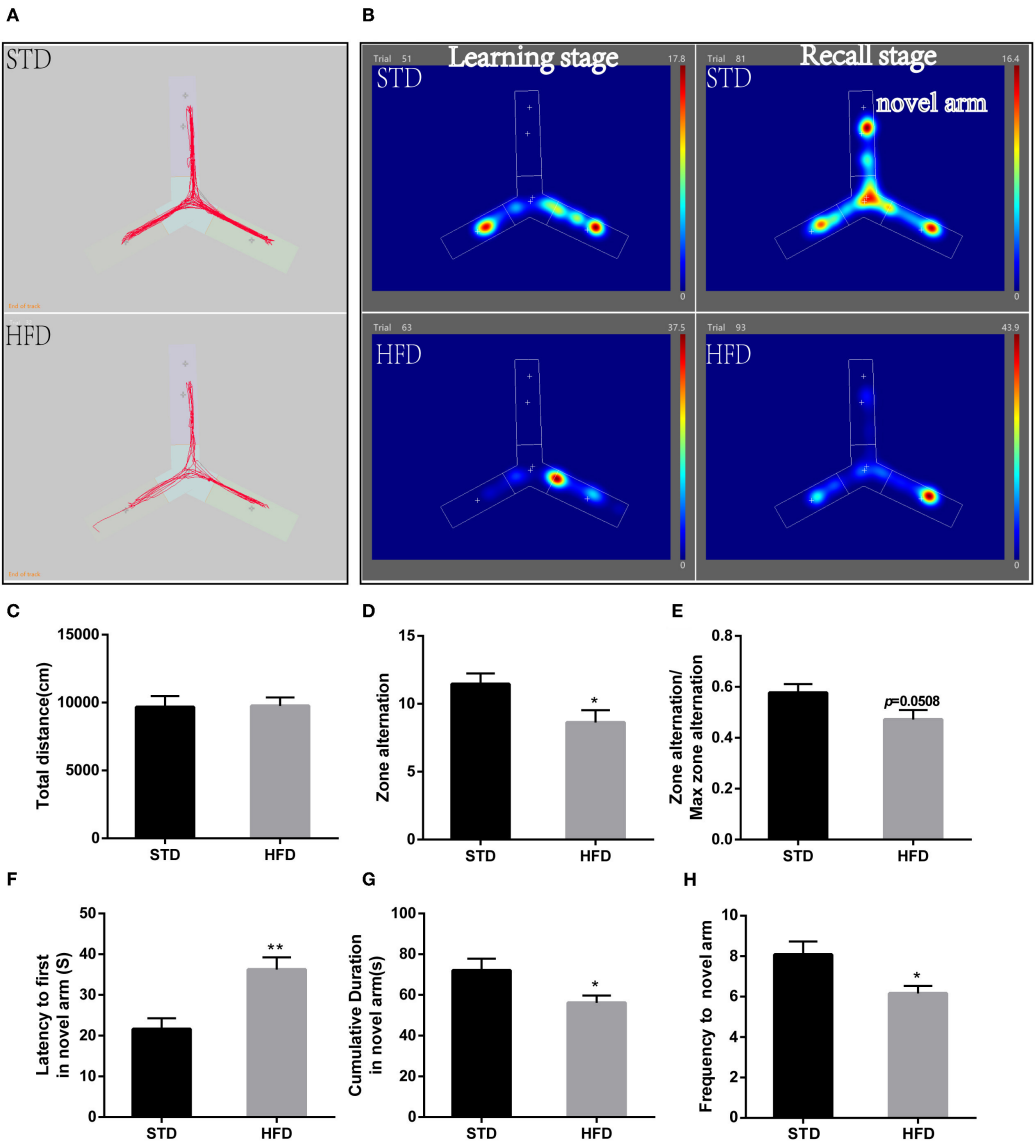
The results of mice during Morris Y-maze test. **(A)** The trajectory map free exploration experiment spontaneous alternating reaction test. **(B)** The heatmap during the process of new environment and space identification experiments. **(C)** The total walking distance of mice during the test. **(D)** Times of zone alteration. **(E)** The percentage of alternation. **(F)** The escape latency of mice in the novel arm during the process of new environment and space identification experiments. **(G)** The escape latency of mice in the novel arm during the process of new environment and space identification experiments. **(H)** The frequency to novel arm during the process of new environment and space identification experiments.*P < 0.05; **P < 0.01. Both in the spontaneous alternating reaction test and the process of new environment and space identification experiments, three replicate experiments were performed for each mouse, and mean values were calculated for final analysis. *n* = 12, 12.

Futhermore, in the Y-maze test, spontaneous alternating reaction test (Figure 3A) showed that walking distance (Figure 3C) of mice have no abvious changes. But, HFD mice had reduced alternations and the percentage of alternation (Figures 3D,E) compared to the STD mice. During the process of new environment and space identification experiments (Figure 3B), HFD mice spent more time escaping to the novel arm for the first time (Figure 3F), and the frequency to the novel arm was significantly less than that of the STD mice, so the exploration time in the novel arm was also significantly lower than that of the control mice (Figures 3G,H). These results are consistent with the results of the water maze, and both proved that HFD mice have impaired spatial recognition memory and that HFD-associated obesity can induce seriously declined cognitive functions.

### Histopathological staining and nissl staining results of hippocampus in HFD-associated obesity mice

**Figure 4:**
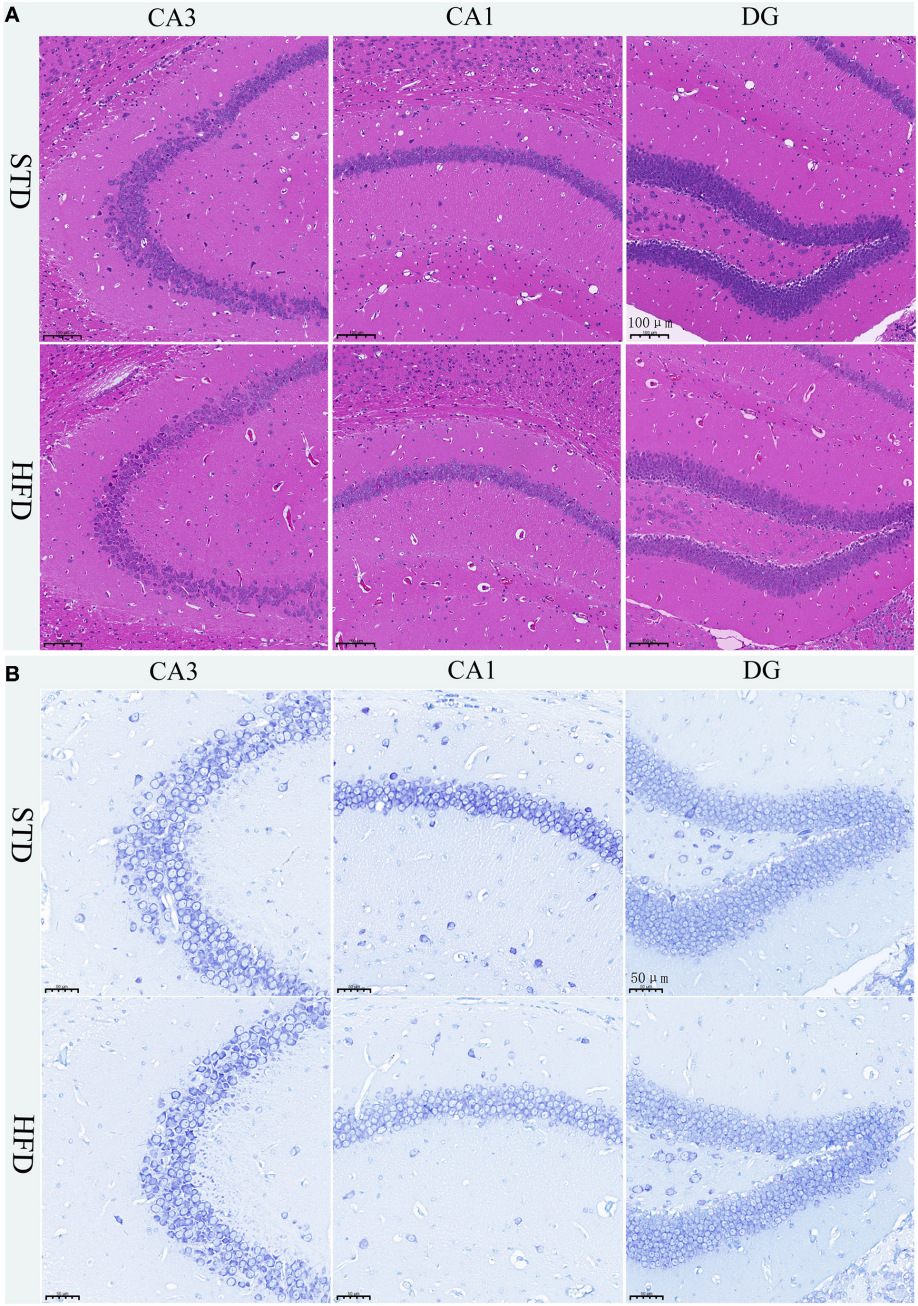
Histopathological staining and Nissl staining of brain tissue in mice. **(A)** HE staining in the hippocampal CA1, CA3, and DG regions for each group. **(B)** Nissl staining in the hippocampal CA1, CA3, and DG regions for each group. Brain tissues from three mice of each group were used for pathological analysis.

The hippocampus is the main brain region that is primarily responsible for learning and memory, and is closely related to memory, stress regulation, and spatial navigation processes. To confirm the changes in neuronal loss and damage to the hippocampus of HFD-induced obesity mice, we used hematoxylin-eosin staining and Nissl staining. The HE stain showed no obvious change in hippocampal neurons in HFD mice compared with controls (Figure 4A). We all know that all kinds of neurons contain Nissl bodies, which are an important part of protein synthesis in neurons. Nissl body will change due to changes in the physiological state. When neurons are stimulated, the Nissl bodies in the cell body will be significantly reduced (Lawal et al., 2021). Therefore, Nissl staining in brain sections was utilized to examine whether the neurons were damaged or not. However, we did not observe any changes in the Nissl bodies of HFD mice when compared to those of the STD mice (Figure 4B). We are also amazed that there were no obvious pathological changes in the hippocampus of HFD-induced obese mice.

### HFD changed circRNA expression map in the hippocampus and changed circrnas probably participating in endoplasmic reticulum stress and synaptic plasticity of neurons

**Figure 5:**
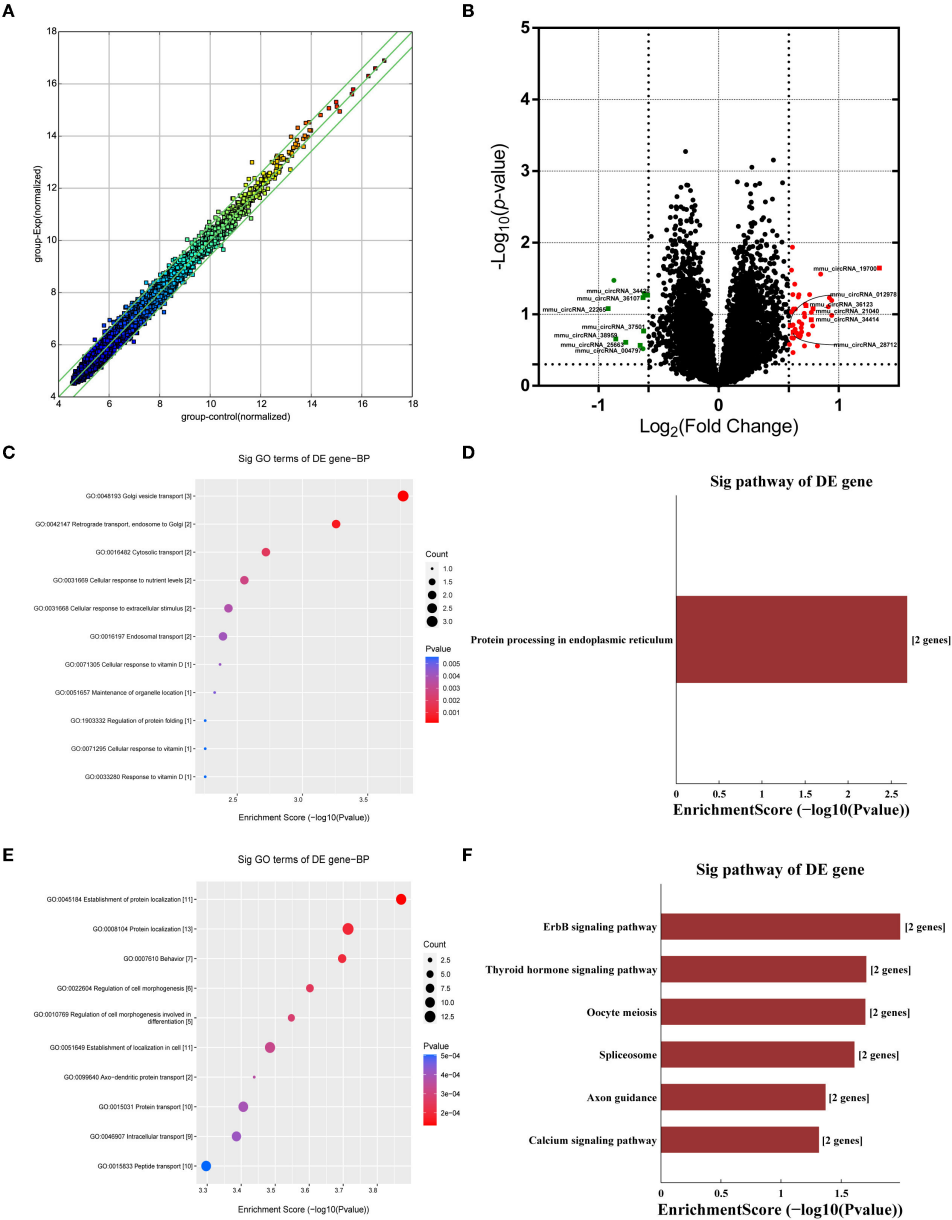
Analysis of circRNA changes in the hippocampus. Five biological replicates of each group were applied in the experiment (*n* = 5, 5). **(A)** The resulting scatter plot of circRNAs. **(B)** Volcano scatter plot of changed circRNAs. Generally, the horizontal axis is Log2 (fold change), The point is further off the center, and the greater the fold difference. The vertical axis is -Log 10 (*P*-value), with points closer to the top of the graph and indicating more significant differences. **(C)** The enrichment score dot plot of BP terms in GO analysis of downregulated circRNAs. **(D)** The KEGG pathway analysis of downregulated circRNAs. **(E)** The enrichment score dot plot of BP terms in GO analysis of upregulated circRNAs. **(F)** The KEGG pathway analysis of upregulated circRNAs.

Many studies have shown that circRNAs are highly enriched in mammalian brain tissue and may be involved in the occurrence and development of diverse biological processes, especially in neurodegenerative diseases (Mehta et al., 2020; Moreno-García et al., 2020; Li et al., 2021). A murine circRNA microarray was used to analyze the circRNA profiles in the hippocampus tissues of HFD-fed mice and the controls. The resulting scatter plot (Figure 5A) displayed the full changes in the hippocampus of HFD mice and control mice. In total, HFD-fed mice had 46 upregulated and 10 downregulated circRNAs (Supplementary Table 1) in tissues compared with the matched littermates, and they were highlighted in the volcano plot, respectively (Figure 5B). Together, obese mice associated with HFD resulted in changes in circRNAs. The differentially expressed circRNAs enriched in HFD mice vs. control mice were further categorized using GO annotation and KEGG pathway analysis. GO annotation was used to identify the potential functional roles of DE-circRNAs through three categories: biological process (BP), molecular function (MF), and cellular component (CC). The result shows that a total of 407 BP terms, 85 MF terms, and 58 CC terms were significantly enriched among the upregulated genes (Supplementary Table 2). Among the downregulated circRNAs, 104 BP terms, 43 MF terms, and 33 CC terms were found by GO analysis (Supplementary Table 3). BP terms of downregulated circRNAs in the HFD mice ranked by enrichment score are shown in Figure 5C, and these processes are summarized as material transport process (GO:0048193, GO:0042147, GO:0016197, and GO:0016482) and cell response to external stimuli, such as cellular response to nutrient levels (GO:0031669, GO:0071295, GO:0033280, and GO:0071305). Furthermore, KEGG pathway analysis showed that only a single significant pathway, that is, “Protein processing in endoplasmic reticulum” (mmu04141) (Figure 5D), was observed in downregulated circRNAs. Intriguingly, BP terms of upregulated circRNAs mainly ranked in “behavior (GO:0007610)” and “axo-dendritic protein transport (GO:0099640)” (Figure 5E), which were related to neuronal function and behavior. And, upregulated circRNAs are mainly involved in Axon guidance (mmu04360), calcium signaling pathway (mmu04020), and ErbB signaling pathway (mmu04012) (Figure 5F). The result hinted that changed circRNA probably participated in endoplasmic reticulum stress and synaptic plasticity of neurons.

**Figure 6:**
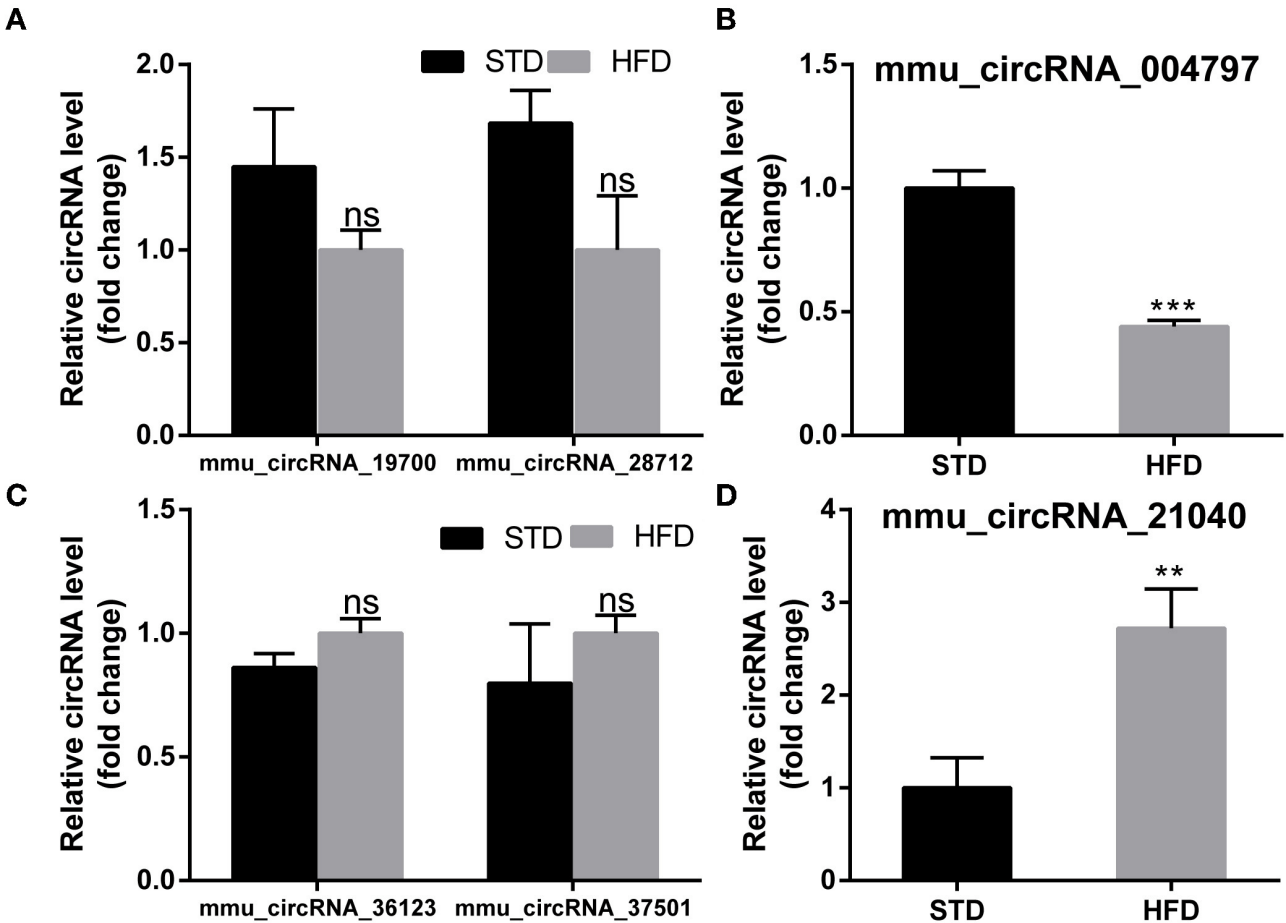
PCR test to identify the expression levels of circRNA candidates in the hippocampus of mice. **(A,B)** PCR Quantification results of down-regulated CircRNAs (mmu-circRNA-19700/28712/004797). **(C,D)** PCR Quantification results of up-regulated circRNAs(mmu-circRNA-36123/37501/21040). The experiments were independently repeated three times in each figure (*n* = 5, 5). **P < 0.01; ***P < 0.001.

**Figure 7:**
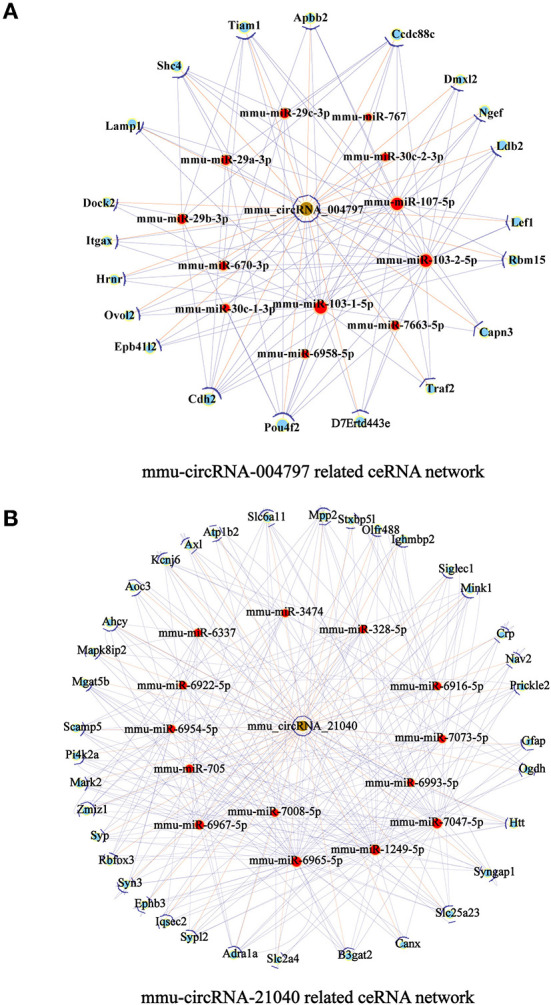
circRNA-miRNA-mRNA interaction network. A circRNA-miRNA-mRNA interaction network was constructed by ceRNA analysis. **(A)** The ceRNA relationship pairs included circRNA-004797 as the core nodes, as well as 12 target miRNAs and 20 target mRNAs. **(B)** The ceRNA relationship pairs included circRNA-21040 as the core nodes, as well as 14 target miRNAs and 36 target mRNAs. Nodes with brown color represent circRNAs, while nodes with light-blue color are protein_coding RNAs. Nodes with red color represent the predicted miRNAs. Edges with T-shape arrows represent directed relationships Edges without arrows represent undirected relationships (ceRNA relationship). ceRNA, competing endogenous RNA; circRNA, circular RNA; miRNA/miR, microRNA.

To further examine the regulatory mechanism underlying DE-circRNAs profiles, ceRNA analysis was performed, and a circRNA-miRNA-mRNA interaction network was constructed. To confirm the ceRNA network, the DE-circRNAs from the circRNA microarray were selected for validation. Seven downregulated circRNAs (mmu_circRNA_28712, mmu_circRNA_19700, mmu_circRNA_21040, mmu_circRNA_36730, mmu_circRNA_36123, mmu_circRNA_34414, and mmu_circRNA_012978) and five upregulated circRNAs (mmu_circRNA_004797, mmu_circRNA_37501, mmu_circRNA_36107, mmu_circRNA_34428, and mmu_circRNA_38959) were selected for validation by RT-qPCR. With RT-qPCR analysis, six circRNAs (Figure 6) were detected. Among them, mmu_circRNA_004797 (Figure 6B) was identified to be significantly downregulated and mmu_circRNA_21040 (Figure 6D) was significantly upregulated in HFD-fed mice, compared with the control mice. Then, the most potential miRNA binding sites predicted by ceRNA analysis for the two validated circRNAs in the ceRNA network are presented in Supplementary Table 5. circRNA-miRNA-mRNA interaction network (Figures 7A,B) of mmu_circRNA_004797 and mmu_circRNA-21040 was constructed separately. In the mmu_circRNA_004797 related network (Figure 7A), 12 target miRNAs (mmu-miR-103-1-5P, mmu-miR-103-2-5P, mmu-miR-107-5P, mmu-miR-30C-1-3P, mmu-miR-30C-2-3P, mmu-miR-29a-3P, mmu-miR-29b-3P, mmu-miR-29c-3P, mmu-miR-6958-5p, mmu-miR-670-3p, mmu-miR-7663-5p, and mmu-miR-767) and 20 genes (Lef1, Epb41l2, Ngef, Dmxl2, Dock2, Ldb2, D7Ertd443e, Lamp1, Pou4f2, Tiam1, Itgax, Capn3, Traf2, Ccdc88c, Cdh2, Shc4, Apbb2, Rbm15, Ovol2, and Hrnr) were predicted. Meanwhile, 14 target miRNAs (mmu-miR-1249-5p, mmu-miR-6965-5p, mmu-miR-7047-5p, mmu-miR-6967-5p, mmu-miR-705, mmu-miR-6922-5p, mmu-miR-7073-5p, mmu-miR-3474, mmu-miR-6916-5p, mmu-miR-6337, mmu-miR-7008-5p, mmu-miR-328-5p, mmu-miR-6993-5p, and mmu-miR-6954-5p) and 36 genes (Aoc3, Mark2, Nav2, Mapk8ip2, Stxbp5l, Mpp2, Ogdh, Syn3, Scamp5, Canx, Crp, Syp, Axl, Olfr488, Ighmbp2, Htt, Prickle2, Sypl2, Ahcy, Syngap1, Mgat5b, B3gat2, Gfap, Rbfox3, Siglec1, Slc25a23, Atp1b2, Kcnj6, Iqsec2, Slc2a4, Ephb3, Zmiz1, Adra1a, Pi4k2a, Mink1, and Slc6a11) were predicted in mmu_circRNA_21040 related network (Figure 7B). It may provide insight into the potential interactions between circRNA candidates and their target genes.

### Activation of ER stress and impairment of synaptic plasticity-related proteins may be related to obesity-associated cognitive decline

**Figure 8:**
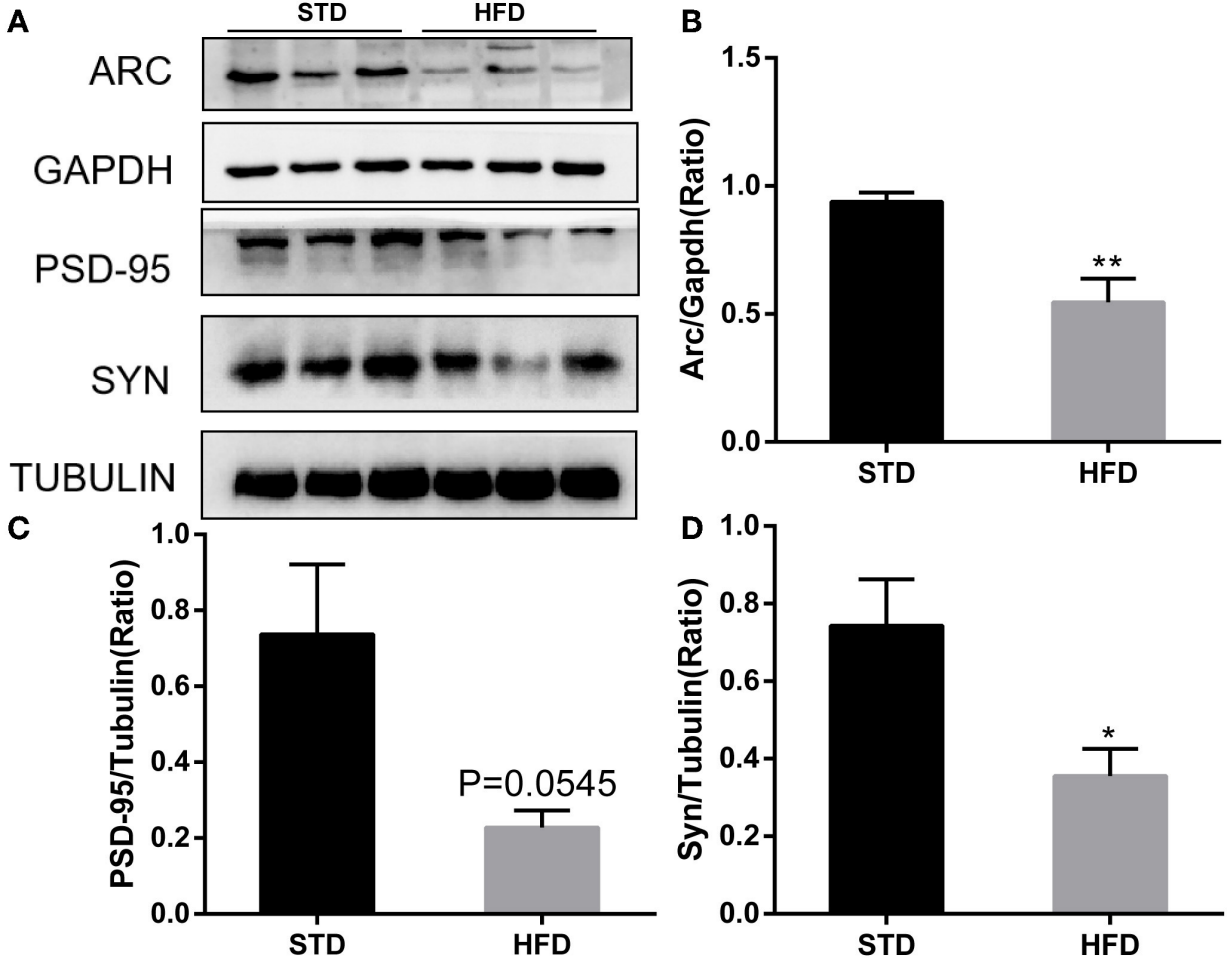
Western blot analyses of ARC, PSD-95, and SYN in hippocampal regions. **(A)** The band of western blot for ARC, PSD-95, and SYN. **(B–D)** Densitometric data of ARC, PSD-95, and SYN expression using ImageJ are presented as the means ± SEM of three independent experiments, *n* = 3, 3. *P < 0.05; **P < 0.01.

**Figure 9:**
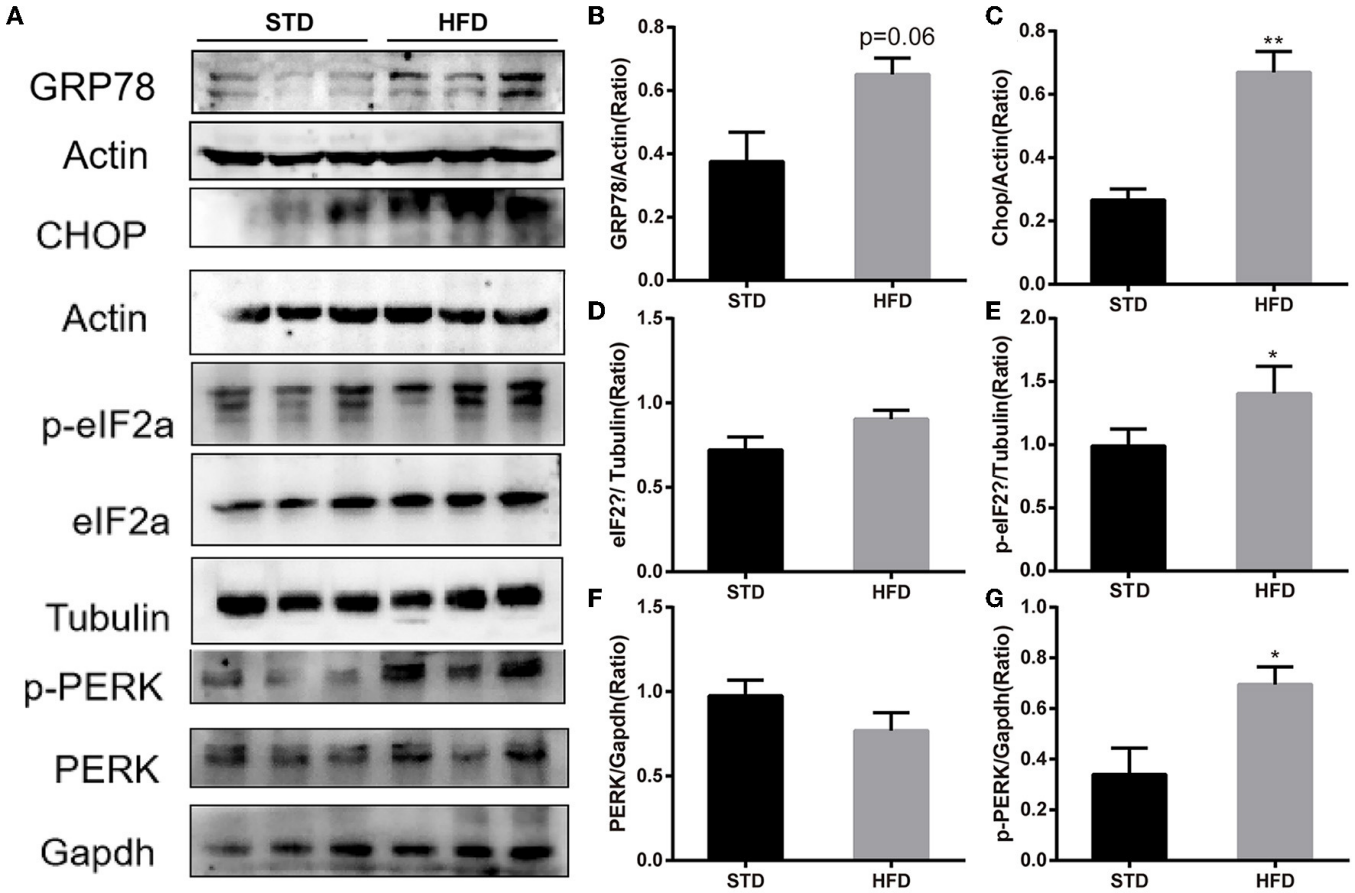
Western blot analyses of GRP78, CHOP, PERK, p-PERK and eIF2α, p-eIF2α in hippocampal. **(A)** The band of western blot for GRP78, CHOP, PERK, p-PERK and eIF2α, p-eIF2α. **(B–G)** Densitometric data of GRP78, CHOP, PERK, p-PERK and eIF2α, p-eIF2α expression using ImageJ are presented as the means ± SEM of 3 independent experiments, *n* = 3,3. *P < 0.05; **P < 0.01.

It has been reported that insulin has important effects on synapses and that HFD may impair hippocampal long-term potentiation. Thus, we examined the deficits of hippocampal synaptic plasticity. For hippocampal synaptic plasticity in HFD mice, we measured the hippocampal expressions of the presynaptic marker synaptophysin (Syn), activity regulated cytoskeleton-associated protein (Arc), and the postsynaptic marker PSD95, which are essential for synaptic plasticity using Western blot analysis. PSD-95 is a membrane-associated scaffolding protein in the excitatory postsynaptic density (PSD) and is a potent regulator of synaptic strength (Ugalde-Triviño and Díaz-Guerra, 2021). As depicted in Figure 8, we found a decrease in the expression of PSD-95 in HFD-fed animals when compared to the control animals. Meanwhile, the expression of synaptophysin, another postsynaptic marker, also decreased obviously. In the HFD group, there was a statistically significant decrease in the expression of activity regulated cytoskeleton-associated protein (Arc) when compared to the control group. Arc is a key molecule for the maintenance of synaptic potentiation and long-term consolidation of memory, and it has been shown to be decreased in neurofibrillary tangle-bearing neurons in the AD brain (Fila et al., 2021). In addition, *in vivo* and *in vitro* studies show that ER stress is associated with high glucose-induced neuron damage and diabetic encephalopathy (Zhu, 2020). As the results show in Figure 9, ER stress was induced under hyperglycemia and dyslipidemia conditions, as the expression of GRP78, CHOP (CCAAT/enhancer binding protein homologous protein) increased, and the significant elevation of P-PERK and P-eIF2α in the hippocampus of HFD-fed mice than in the STD group was observed. In total, the hippocampal expression of proteins which are essential for synaptic plasticity was decreased by HFD, and the levels of ER stress-related proteins were increased by HFD in the hippocampus in our HFD obese mouse model. These results indicated that changes in hippocampal synaptic plasticity and endoplasmic reticulum stress contributed to the cognitive decline induced by HFD.

## Discussion

Currently, HFD-induced obesity and the consequent metabolic dysfunctions are strongly linked to cognitive impairment and dementia. It has been well-documented in various rodent models and indicated that the occurrence and development of cognitive dysfunction is closely related to insulin resistance, oxidative stress, inflammation, and endoplasmic reticulum stress (Kothari et al., 2017; You et al., 2020; Davis et al., 2021). Consistent with previous research results, we found that mice were more susceptible to weight gain, elevated blood glucose, glucose tolerance, and insulin resistance following feeding with HFD, and mice also exhibited cognitive impairment in response to HFD. Additionally, it has been reported that HFD-induced morphology and neuron injury in the hippocampus can be observed by moderate cytoarchitectural abnormalities, widened intercellular spaces, loosely arranged cells, and larger nuclei/cells in CA1 and CA3 of the hippocampus (Wu et al., 2019). However, it was surprising to us that when fed an HFD for 16 weeks, mice exhibited an obese phenotype and cognitive decline but no significant pathological changes in hippocampal neurons. We speculate that obesity induced by HFD is a chronic developmental process and the damage to hippocampal neurons in brain tissue is non-acute and requires a certain degree of cumulative effect.

With the development of genome-wide analysis, RNA sequencing technology, and microarray analysis, increasing evidence indicates that circRNAs play an important role in various diseases, and CeRNA networks involving circRNAs, miRNAs, and mRNAs have been detected in many diseases, including cancers and AD-associated pathophysiology (Wang et al., 2018; Zhang Y. et al., 2019; Zhang et al., 2022). Although, it is well-established that circRNAs play an essential role in regulating neurodegenerative diseases, such as AD (Lo et al., 2020) and PD (Hanan et al., 2020), the mechanisms that respond to circRNAs changes in obesity-associated cognitive decline are not well-explored. Therefore, it is particularly important to identify possible DEcircRNA-miRNA-mRNA interaction networks in obesity-associated cognitive decline. Expectedly, we examined the levels of circRNAs in the hippocampi of mice fed with a standard or high-fat diet using a circRNA Arraystar analysis. Our results showed that HFD-induced obesity mice have differentially expressed circRNA maps with 46 upregulated and 10 downregulated circRNAs compared with the mice fed with the standard diet. Furthermore, we preliminarily screened some differentially expressed circRNA to verify their expression by real-time quantitative PCR, in order to find the characteristic circular RNA molecules involved in obesity-associated cognitive dysfunction and their possible regulatory mechanisms. The results showed that among the DE-circRNA candidates, the level of mmu-circRNA-21040 significantly increased, while the level of mmu-circRNA-004797 significantly decreased. Afterward, we used bioinformatics analysis to predict its associated DEcircRNA-miRNA-mRNA interaction network. circRNA-miRNA-mRNA interaction network (Figures 7A,B) of the mmu_circRNA_004797 and mmu_circRNA-21040 was constructed separately. Each circRNA and its potential complementary binding miRNAs were illustrated by the network, and specific interactions, such as mmu_circRNA_21040/mmu_miR_6965_5p/Syn3 and mmu_circRNA_21040/mmu_miR_29c_3p/Cdh2, were predicted in the ceRNA network. Among the predicted genes, Syn3, Syp, Sypl2, Syngap1, and Cdh2 are associated with synapses and nervous system development. The results provide a reference for the clinical diagnosis and treatment of obesity-associated cognitive dysfunction, and it needs to be validated in the future.

It has been accepted that the hippocampus is the main functional area responsible for learning and memory; hippocampus dysfunction is considered to be a key and central mechanism that underlies cognitive impairment (Gannon et al., 2022). Of course, neuronal synaptic plasticity has long been considered an important component and the neural basis of learning and memory (Neves et al., 2008), and it has been reported that HFD can damage the synaptic plasticity in the hippocampal neurons of obese mice (O'Brien et al., 2017). In this study, the results of GO analysis and KEGG pathway analysis indicated that DE-circRNAs may be involved in cell responses to external environmental stimuli, neuronal development, and protein processing in the endoplasmic reticulum. Thus, combined with the result of GO analysis and KEGG pathway analysis of DE-circRNAs, we detected the expression of neuronal synaptic plasticity markers in the hippocampus. In accordance with previous studies, the expression levels of neuronal synaptic plasticity-related proteins (Arc, Syn, and PSD-95 significantly decreased compared with the mice in the normal diet group. Additionally, disturbance in protein homeostasis is characteristic of many diseases, including diabetes and neurological complications. The endoplasmic reticulum (ER) plays a major role in protein synthesis and in the folding and processing of secreted and transmembrane proteins (Cho et al., 2020). Many metabolic stimuli (high blood sugar and high-fat diet) can lead to the accumulation of unfolded or misfolded proteins in the lumen of the endoplasmic reticulum, a condition known as “ER stress” (Zhu, 2020). Increasing evidence indicated that ER stress is involved in the pathogenesis of metabolic pathologies, and the number of diseases associated with ER stress has also increased. *In vitro* and *in vivo* studies have shown that endoplasmic reticulum stress is associated with high glucose-induced neuronal damage and diabetic encephalopathy (Peng et al., 2022). We further detected the levels of ER stress, such as GPR78, PERK, and eIF2α. The results showed that the expression of GPR78 and phosphorylation of PERK and eIF2α increased in HFD mice, indicating that high-fat diet-induced activation of endoplasmic reticulum stress. These results may provide a reference that circRNAs may be involved in high-fat diet-induced obesity-related cognitive dysfunction by affecting the endoplasmic reticulum stress and synaptic plasticity of hippocampal neurons, and it requires further experiments to verify.

## Conclusion

In this study, we found that mice were more susceptible to obesity when fed with a high-fat diet, mainly manifested as significant weight gain, increased blood glucose, glucose tolerance, insulin resistance, and metabolism disorders, which were consistent with previous reports. Interestingly, we found that HFD causes obesity-associated cognitive impairment in mice. In addition, HFD-induced obesity mice showed changes in cognitive function earlier than the onset of pathological changes in the brain tissue. In addition, impaired synaptic plasticity and activation of endoplasmic reticulum stress in hippocampal neurons of mice with obesity-associated cognitive dysfunction were observed.

Furthermore, based on the results of hippocampus circRNA analysis and the expression of brain cognition-related molecules, we found that obese mice induced by HFD exhibited a changed expression map of circRNA. Verified by PCR test, the expression of circRNA-004797 decreased and the level of circRNA-21040 increased significantly. Changes in circRNAs can be a reference before the histopathological changes, and the result can provide proof that circRNA may be a biomarker for obesity-associated cognitive impairment. Overall, our study suggested that circRNAs play vital roles in obesity-associated cognitive impairment. However, further investigation of obesity-associated cognitive decline in mice after being fed a long-term HFD is required. We have set out to investigate the molecular functions and regulatory mechanisms of mmu-circRNA-004797 and mmu-circRNA-21040 in obesity-associated cognitive dysfunction in the next study.

## Data availability statement

The Microarray data were uploaded to the Gene Expression Omnibus database704 (https://www.ncbi.nlm.nih.gov/geo/). The GEO accession number is GEO: GSE208344.

## Ethics statement

The animal study was reviewed and approved by Ethics Committee of Xi'an International Medical Center Hospital.

## Author contributions

JY, BC, and YN contributed to the experimental design and writing of the manuscript. TL, XS, and MZ contributed to animal experiment data acquisition. PC, HZ, and SL contributed to data analysis and result interpretation. All authors have read and approved the final manuscript.

## Funding

This work was supported by the National Natural Science Foundation of China (Grant No. 81870172), the Shaanxi Provincial Key Research and Development Project (Grant No. 2018ZDXM-SF-068), and the Annual Project of Xi'an International Medical Center Hospital (Grant No. 2020QN007), Shaanxi Province.

## Conflict of interest

The authors declare that the research was conducted in the absence of any commercial or financial relationships that could be construed as a potential conflict of interest.

## Publisher's note

All claims expressed in this article are solely those of the authors and do not necessarily represent those of their affiliated organizations, or those of the publisher, the editors and the reviewers. Any product that may be evaluated in this article, or claim that may be made by its manufacturer, is not guaranteed or endorsed by the publisher.
