# Supplementary material for: Obese mice induced by high-fat diet have differential expression of circular RNAs involved in endoplasmic reticulum stress and neuronal synaptic plasticity of hippocampus leading to obesity-associated cognitive impairment
Source: Front Mol Neurosci. 2022 Oct 3;15:1000482. doi: 10.3389/fnmol.2022.1000482 (PMC9574125; doi:10.3389/fnmol.2022.1000482)
Supplement: Supplementary file 1 [file Data_Sheet_1.docx]

Supplementary Material

# Supplementary Tables

## 1.1 Supplementary Table1

Summary of change trends of circRNAs in hippocampus of HFD-fed mice compared with controls in relative integral levels

|  | **circRNA** | **P-value** | **FC** | **GeneSymbol** |
| --- | --- | --- | --- | --- |
| **Down-regulated** | mmu_circRNA_004797 | 0.272837861 | 1.5737 | Tbc1d14 |
|  | mmu_circRNA_19867 | 0.033700772 | 1.8327 | Khdrbs2 |
|  | mmu_circRNA_22265 | 0.083512728 | 1.8933 | Trappc10 |
|  | mmu_circRNA_25663 | 0.246176865 | 1.7105 | Foxn3 |
|  | mmu_circRNA_28916 | 0.051008331 | 1.5375 | Ttll8 |
|  | mmu_circRNA_37501 | 0.171741177 | 1.5437 | Ptprf |
|  | mmu_circRNA_36107 | 0.058767532 | 1.5479 | Sec24d |
|  | mmu_circRNA_25647 | 0.30432947 | 1.5476 | Eml5 |
|  | mmu_circRNA_34428 | 0.054066954 | 1.5084 | Pdia3 |
|  | mmu_circRNA_38959 | 0.221753774 | 1.8120 | Alb |
| **up-regulated** | mmu_circRNA_015487 | 0.171647869 | 1.5479 | Tead2 |
|  | mmu_circRNA_29641 | 0.058944715 | 1.8966 | Iqcb1 |
|  | mmu_circRNA_24245 | 0.064149524 | 1.9216 | Cdc27 |
|  | mmu_circRNA_26984 | 0.053265418 | 1.6912 | Dhx29 |
|  | mmu_circRNA_43813 | 0.093248764 | 1.5220 | Zfp426 |
|  | mmu_circRNA_45592 | 0.200143582 | 1.5829 | XLOC_027202 |
|  | mmu_circRNA_43571 | 0.184220672 | 1.5723 | Klhdc4 |
|  | mmu_circRNA_012978 | 0.143631342 | 1.5130 | Tsc2 |
|  | mmu_circRNA_39338 | 0.053571302 | 1.5893 | Tmem120b |
|  | mmu_circRNA_38070 | 0.197056613 | 1.6039 | Cfap69 |
|  | mmu_circRNA_41580 | 0.057367824 | 1.5843 |  |
|  | mmu_circRNA_29088 | 0.207292716 | 1.5985 | Tfcp2 |
|  | mmu_circRNA_32776 | 0.011577458 | 1.5314 | Vti1a |
|  | mmu_circRNA_006065 | 0.276912517 | 1.7688 | Fam46c |
|  | mmu_circRNA_29347 | 0.141155621 | 1.5357 | Spag6 |
|  | mmu_circRNA_32332 | 0.176798286 | 1.6148 | Fxn |
|  | mmu_circRNA_30352 | 0.084601852 | 1.5402 | Rab11fip3 |
|  | mmu_circRNA_31187 | 0.276993175 | 1.6413 | Svil |
|  | mmu_circRNA_41226 | 0.126686717 | 1.5905 | 4732471J01Rik |
|  | mmu_circRNA_32706 | 0.024174129 | 1.5221 | Cnnm2 |
|  | mmu_circRNA_29625 | 0.027442893 | 1.8026 | Pdia5 |
|  | mmu_circRNA_24800 | 0.216514026 | 1.5404 | Grhl1 |
|  | mmu_circRNA_25839 | 0.078358459 | 1.8841 | Mirg |
|  | mmu_circRNA_013053 | 0.191414493 | 1.6794 | Gmcl1 |
|  | mmu_circRNA_19677 | 0.161908841 | 1.5542 | Ncoa2 |
|  | mmu_circRNA_19469 | 0.072519592 | 1.6480 | Trim21 |
|  | mmu_circRNA_28882 | 0.103731296 | 1.9218 | 1810041L15Rik |
|  | mmu_circRNA_38377 | 0.094384253 | 1.7073 | Gtf3c2 |
|  | mmu_circRNA_28712 | 0.178560722 | 1.5386 | Ptk2 |
|  | mmu_circRNA_33569 | 0.192008782 | 1.5978 | Fmnl2 |
|  | mmu_circRNA_19555 | 0.108579008 | 1.6421 | XLOC_027457 |
|  | mmu_circRNA_41279 | 0.037969949 | 1.5507 | U2af1l4 |
|  | mmu_circRNA_36800 | 0.136412255 | 1.6161 | Fktn |
|  | mmu_circRNA_34712 | 0.342773147 | 1.5363 | Sec23b |
|  | mmu_circRNA_29724 | 0.179516106 | 1.5625 | Lsamp |
|  | mmu_circRNA_21040 | 0.084101644 | 1.7244 | Cacna1e |
|  | mmu_circRNA_28050 | 0.145521919 | 1.7211 | Rap2a |
|  | mmu_circRNA_36730 | 0.097374171 | 1.6385 | Ncbp1 |
|  | mmu_circRNA_34414 | 0.119733616 | 1.7077 | Map1a |
|  | mmu_circRNA_001946 | 0.084563935 | 1.5568 | Cdr1 |
|  | mmu_circRNA_26909 | 0.263326255 | 1.5039 | Cwc27 |
|  | mmu_circRNA_26931 | 0.213658399 | 1.5269 | Ipo11 |
|  | mmu_circRNA_36123 | 0.075836964 | 1.6561 | Camk2d |
|  | mmu_circRNA_25129 | 0.053008528 | 1.5347 |  |
|  | mmu_circRNA_19700 | 0.022651758 | 2.5294 | Stau2 |
|  | mmu_circRNA_21410 | 0.154344235 | 1.6113 | Ahctf1 |

## 1.2 Supplementary Table2

The down-regulated circRNAs identified in HFD-fed mice vs control were categorized using GO annotation.

| **Biologic process** | | | | | | | |
| --- | --- | --- | --- | --- | --- | --- | --- |
| **GO.ID** | **Term** | **Count** | **Fold.Enrichment** | **Pvalue** | **Enrichment.Score** | **Gene.Ratio** | **GENES** |
| **GO:0048193** | **Golgi vesicle transport** | **3** | **26.11343284** | **0.0001694961** | **3.7708404021** | **0.3** | **SEC24D//TRAPPC10//TBC1D14** |
| **GO:0042147** | **retrograde transport, endosome to Golgi** | **2** | **56.21204819** | **0.0005524880** | **3.2576771540** | **0.2** | **TRAPPC10//TBC1D14** |
| **GO:0016482** | **cytosolic transport** | **2** | **30.10064516** | **0.0019060321** | **2.7198697837** | **0.2** | **TRAPPC10//TBC1D14** |
| **GO:0031669** | **cellular response to nutrient levels** | **2** | **24.81702128** | **0.0027861097** | **2.5550017809** | **0.2** | **ALB//PDIA3** |
| **GO:0031668** | **cellular response to extracellular stimulus** | **2** | **21.50046083** | **0.0036900344** | **2.4329695867** | **0.2** | **ALB//PDIA3** |
| **GO:0016197** | **endosomal transport** | **2** | **20.46315789** | **0.0040642879** | **2.3910155389** | **0.2** | **TRAPPC10//TBC1D14** |
| **GO:0071305** | **cellular response to vitamin D** | **1** | **233.28** | **0.0042792584** | **2.3686314873** | **0.1** | **PDIA3** |
| **GO:0051657** | **maintenance of organelle location** | **1** | **212.0727273** | **0.0047062765** | **2.3273225628** | **0.1** | **ALB** |
| **GO:0033280** | **response to vitamin D** | **1** | **179.4461538** | **0.0055598182** | **2.2549394079** | **0.1** | **PDIA3** |
| **GO:0071295** | **cellular response to vitamin** | **1** | **179.4461538** | **0.0055598182** | **2.2549394079** | **0.1** | **PDIA3** |
| **GO:1903332** | **regulation of protein folding** | **1** | **179.4461538** | **0.0055598182** | **2.2549394079** | **0.1** | **PDIA3** |
| **GO:0045188** | **regulation of circadian sleep/wake cycle, non-REM sleep** | **1** | **166.6285714** | **0.0059863420** | **2.2228384762** | **0.1** | **ALB** |
| **GO:0045938** | **positive regulation of circadian sleep/wake cycle, sleep** | **1** | **166.6285714** | **0.0059863420** | **2.2228384762** | **0.1** | **ALB** |
| **GO:0090110** | **COPII-coated vesicle cargo loading** | **1** | **166.6285714** | **0.0059863420** | **2.2228384762** | **0.1** | **SEC24D** |
| **GO:0071496** | **cellular response to external stimulus** | **2** | **16.66285714** | **0.0060620265** | **2.2173821681** | **0.2** | **ALB//PDIA3** |
| **GO:0042748** | **circadian sleep/wake cycle, non-REM sleep** | **1** | **155.52** | **0.0064127011** | **2.1929590015** | **0.1** | **ALB** |
| **GO:1900121** | **negative regulation of receptor binding** | **1** | **155.52** | **0.0064127011** | **2.1929590015** | **0.1** | **PTPRF** |
| **GO:0051386** | **regulation of neurotrophin TRK receptor signaling pathway** | **1** | **145.8** | **0.0068388956** | **2.1650140235** | **0.1** | **PTPRF** |
| **GO:0097094** | **craniofacial suture morphogenesis** | **1** | **145.8** | **0.0068388956** | **2.1650140235** | **0.1** | **FOXN3** |
| **GO:0035459** | **vesicle cargo loading** | **1** | **137.2235294** | **0.0072649256** | **2.1387688274** | **0.1** | **SEC24D** |
| **GO:0098760** | **response to interleukin-7** | **1** | **137.2235294** | **0.0072649256** | **2.1387688274** | **0.1** | **PDIA3** |
| **GO:0098761** | **cellular response to interleukin-7** | **1** | **137.2235294** | **0.0072649256** | **2.1387688274** | **0.1** | **PDIA3** |
| **GO:0031667** | **response to nutrient levels** | **2** | **13.48439306** | **0.0091241038** | **2.0398097815** | **0.2** | **ALB//PDIA3** |
| **GO:0031670** | **cellular response to nutrient** | **1** | **97.2** | **0.0102425332** | **1.9895926214** | **0.1** | **PDIA3** |
| **GO:0090114** | **COPII-coated vesicle budding** | **1** | **93.312** | **0.0106672489** | **1.9719475730** | **0.1** | **SEC24D** |
| **GO:0009991** | **response to extracellular stimulus** | **2** | **12.34285714** | **0.0108130179** | **1.9660530770** | **0.2** | **ALB//PDIA3** |
| **GO:0006891** | **intra-Golgi vesicle-mediated transport** | **1** | **86.4** | **0.0115161882** | **1.9386912457** | **0.1** | **TRAPPC10** |
| **GO:0042059** | **negative regulation of epidermal growth factor receptor signaling pathway** | **1** | **86.4** | **0.0115161882** | **1.9386912457** | **0.1** | **PTPRF** |
| **GO:0042753** | **positive regulation of circadian rhythm** | **1** | **86.4** | **0.0115161882** | **1.9386912457** | **0.1** | **ALB** |
| **GO:1900120** | **regulation of receptor binding** | **1** | **86.4** | **0.0115161882** | **1.9386912457** | **0.1** | **PTPRF** |
| **GO:0033273** | **response to vitamin** | **1** | **80.44137931** | **0.0123644719** | **1.9078244281** | **0.1** | **PDIA3** |
| **GO:0045187** | **regulation of circadian sleep/wake cycle, sleep** | **1** | **80.44137931** | **0.0123644719** | **1.9078244281** | **0.1** | **ALB** |
| **GO:1901185** | **negative regulation of ERBB signaling pathway** | **1** | **80.44137931** | **0.0123644719** | **1.9078244281** | **0.1** | **PTPRF** |
| **GO:0050802** | **circadian sleep/wake cycle, sleep** | **1** | **77.76** | **0.0127883680** | **1.8931848749** | **0.1** | **ALB** |
| **GO:0018200** | **peptidyl-glutamic acid modification** | **1** | **75.2516129** | **0.0132121004** | **1.8790281363** | **0.1** | **TTLL8** |
| **GO:0048011** | **neurotrophin TRK receptor signaling pathway** | **1** | **75.2516129** | **0.0132121004** | **1.8790281363** | **0.1** | **PTPRF** |
| **GO:0042749** | **regulation of circadian sleep/wake cycle** | **1** | **72.9** | **0.0136356690** | **1.8653235493** | **0.1** | **ALB** |
| **GO:0099560** | **synaptic membrane adhesion** | **1** | **72.9** | **0.0136356690** | **1.8653235493** | **0.1** | **PTPRF** |
| **GO:0022410** | **circadian sleep/wake cycle process** | **1** | **70.69090909** | **0.0140590740** | **1.8520432823** | **0.1** | **ALB** |
| **GO:0048194** | **Golgi vesicle budding** | **1** | **68.61176471** | **0.0144823155** | **1.8391619966** | **0.1** | **SEC24D** |
| **GO:0042745** | **circadian sleep/wake cycle** | **1** | **64.8** | **0.0153283078** | **1.8145057869** | **0.1** | **ALB** |
| **GO:0048679** | **regulation of axon regeneration** | **1** | **64.8** | **0.0153283078** | **1.8145057869** | **0.1** | **PTPRF** |
| **GO:2000785** | **regulation of autophagosome assembly** | **1** | **64.8** | **0.0153283078** | **1.8145057869** | **0.1** | **TBC1D14** |
| **GO:0035335** | **peptidyl-tyrosine dephosphorylation** | **1** | **61.38947368** | **0.0161736465** | **1.7911920530** | **0.1** | **PTPRF** |
| **GO:0046627** | **negative regulation of insulin receptor signaling pathway** | **1** | **61.38947368** | **0.0161736465** | **1.7911920530** | **0.1** | **PTPRF** |
| **GO:0030431** | **sleep** | **1** | **58.32** | **0.0170183320** | **1.7690830081** | **0.1** | **ALB** |
| **GO:0038179** | **neurotrophin signaling pathway** | **1** | **58.32** | **0.0170183320** | **1.7690830081** | **0.1** | **PTPRF** |
| **GO:0048520** | **positive regulation of behavior** | **1** | **58.32** | **0.0170183320** | **1.7690830081** | **0.1** | **ALB** |
| **GO:0070570** | **regulation of neuron projection regeneration** | **1** | **58.32** | **0.0170183320** | **1.7690830081** | **0.1** | **PTPRF** |
| **GO:1900077** | **negative regulation of cellular response to insulin stimulus** | **1** | **55.54285714** | **0.0178623647** | **1.7480610469** | **0.1** | **PTPRF** |
| **GO:0046907** | **intracellular transport** | **3** | **4.988168211** | **0.0189312886** | **1.7228198248** | **0.3** | **TBC1D14//SEC24D//TRAPPC10** |
| **GO:0044088** | **regulation of vacuole organization** | **1** | **51.84** | **0.0191271909** | **1.7183488076** | **0.1** | **TBC1D14** |
| **GO:0051646** | **mitochondrion localization** | **1** | **49.63404255** | **0.0199695937** | **1.6996307711** | **0.1** | **ALB** |
| **GO:0051641** | **cellular localization** | **4** | **3.401822822** | **0.0223189823** | **1.6513256116** | **0.4** | **TBC1D14//SEC24D//TRAPPC10//ALB** |
| **GO:0001960** | **negative regulation of cytokine-mediated signaling pathway** | **1** | **44.01509434** | **0.0224928968** | **1.6479546099** | **0.1** | **PTPRF** |
| **GO:0031103** | **axon regeneration** | **1** | **44.01509434** | **0.0224928968** | **1.6479546099** | **0.1** | **PTPRF** |
| **GO:0042981** | **regulation of apoptotic process** | **3** | **4.613315755** | **0.0233124851** | **1.6324114278** | **0.3** | **PTPRF//ALB//PDIA3** |
| **GO:0050775** | **positive regulation of dendrite morphogenesis** | **1** | **42.41454545** | **0.0233326975** | **1.6320350493** | **0.1** | **PTPRF** |
| **GO:0060761** | **negative regulation of response to cytokine stimulus** | **1** | **40.92631579** | **0.0241718488** | **1.6166901303** | **0.1** | **PTPRF** |
| **GO:0043067** | **regulation of programmed cell death** | **3** | **4.54146658** | **0.0243019312** | **1.6143592125** | **0.3** | **PTPRF//ALB//PDIA3** |
| **GO:0016192** | **vesicle-mediated transport** | **3** | **4.471821086** | **0.0253148610** | **1.5966244533** | **0.3** | **SEC24D//TRAPPC10//TBC1D14** |
| **GO:2001238** | **positive regulation of extrinsic apoptotic signaling pathway** | **1** | **38.88** | **0.0254293592** | **1.5946645837** | **0.1** | **PDIA3** |
| **GO:0007584** | **response to nutrient** | **1** | **38.24262295** | **0.0258482051** | **1.5875696091** | **0.1** | **PDIA3** |
| **GO:0006900** | **vesicle budding from membrane** | **1** | **37.62580645** | **0.0262668890** | **1.5805913616** | **0.1** | **SEC24D** |
| **GO:0031102** | **neuron projection regeneration** | **1** | **37.62580645** | **0.0262668890** | **1.5805913616** | **0.1** | **PTPRF** |
| **GO:0048512** | **circadian behavior** | **1** | **37.62580645** | **0.0262668890** | **1.5805913616** | **0.1** | **ALB** |
| **GO:0007622** | **rhythmic behavior** | **1** | **36.45** | **0.0271037709** | **1.5669702819** | **0.1** | **ALB** |
| **GO:0045454** | **cell redox homeostasis** | **1** | **35.88923077** | **0.0275219691** | **1.5603204972** | **0.1** | **PDIA3** |
| **GO:0042058** | **regulation of epidermal growth factor receptor signaling pathway** | **1** | **33.80869565** | **0.0291931444** | **1.5347191243** | **0.1** | **PTPRF** |
| **GO:0048678** | **response to axon injury** | **1** | **33.80869565** | **0.0291931444** | **1.5347191243** | **0.1** | **PTPRF** |
| **GO:0043065** | **positive regulation of apoptotic process** | **2** | **7.233488372** | **0.0296488767** | **1.5279917558** | **0.2** | **PTPRF//PDIA3** |
| **GO:0043068** | **positive regulation of programmed cell death** | **2** | **7.177846154** | **0.0300762303** | **1.5217765984** | **0.2** | **PTPRF//PDIA3** |
| **GO:1904888** | **cranial skeletal system development** | **1** | **32.4** | **0.0304448292** | **1.5164864577** | **0.1** | **FOXN3** |
| **GO:0000381** | **regulation of alternative mRNA splicing, via spliceosome** | **1** | **31.95616438** | **0.0308617346** | **1.5105796677** | **0.1** | **KHDRBS2** |
| **GO:1901184** | **regulation of ERBB signaling pathway** | **1** | **31.52432432** | **0.0312784787** | **1.5047543787** | **0.1** | **PTPRF** |
| **GO:0010941** | **regulation of cell death** | **3** | **4.119128899** | **0.0314040770** | **1.5030139659** | **0.3** | **PTPRF//ALB//PDIA3** |
| **GO:0010507** | **negative regulation of autophagy** | **1** | **31.104** | **0.0316950614** | **1.4990084026** | **0.1** | **TBC1D14** |
| **GO:0046626** | **regulation of insulin receptor signaling pathway** | **1** | **31.104** | **0.0316950614** | **1.4990084026** | **0.1** | **PTPRF** |
| **GO:0043525** | **positive regulation of neuron apoptotic process** | **1** | **28.8** | **0.0341911738** | **1.4660859888** | **0.1** | **PTPRF** |
| **GO:0000380** | **alternative mRNA splicing, via spliceosome** | **1** | **27.44470588** | **0.0358520300** | **1.4454862490** | **0.1** | **KHDRBS2** |
| **GO:0010942** | **positive regulation of cell death** | **2** | **6.525314685** | **0.0358595039** | **1.4453957234** | **0.2** | **PTPRF//PDIA3** |
| **GO:0033554** | **cellular response to stress** | **3** | **3.892324805** | **0.0363772299** | **1.4391703750** | **0.3** | **ALB//PTPRF//PDIA3** |
| **GO:1900076** | **regulation of cellular response to insulin stimulus** | **1** | **25.92** | **0.0379244852** | **1.4210803057** | **0.1** | **PTPRF** |
| **GO:0000045** | **autophagosome assembly** | **1** | **25.35652174** | **0.0387523441** | **1.4117020227** | **0.1** | **TBC1D14** |
| **GO:0090630** | **activation of GTPase activity** | **1** | **25.35652174** | **0.0387523441** | **1.4117020227** | **0.1** | **TBC1D14** |
| **GO:0006915** | **apoptotic process** | **3** | **3.740459647** | **0.0403027976** | **1.3946648059** | **0.3** | **PTPRF//ALB//PDIA3** |
| **GO:1905037** | **autophagosome organization** | **1** | **24.04948454** | **0.0408191870** | **1.3891356497** | **0.1** | **TBC1D14** |
| **GO:0050795** | **regulation of behavior** | **1** | **23.328** | **0.0420573720** | **1.3761578678** | **0.1** | **ALB** |
| **GO:0012501** | **programmed cell death** | **3** | **3.650704225** | **0.0428889190** | **1.3676549001** | **0.3** | **PTPRF//ALB//PDIA3** |
| **GO:0007173** | **epidermal growth factor receptor signaling pathway** | **1** | **22.64854369** | **0.0432941184** | **1.3635710994** | **0.1** | **PTPRF** |
| **GO:0060349** | **bone morphogenesis** | **1** | **22.43076923** | **0.0437060477** | **1.3594584642** | **0.1** | **FOXN3** |
| **GO:0032091** | **negative regulation of protein binding** | **1** | **21.6** | **0.0453521693** | **1.3434019350** | **0.1** | **PTPRF** |
| **GO:0070925** | **organelle assembly** | **2** | **5.717647059** | **0.0456458148** | **1.3405990366** | **0.2** | **TTLL8//TBC1D14** |
| **GO:0001959** | **regulation of cytokine-mediated signaling pathway** | **1** | **21.40183486** | **0.0457633010** | **1.3394826566** | **0.1** | **PTPRF** |
| **GO:0051668** | **localization within membrane** | **1** | **21.40183486** | **0.0457633010** | **1.3394826566** | **0.1** | **SEC24D** |
| **GO:1900006** | **positive regulation of dendrite development** | **1** | **21.40183486** | **0.0457633010** | **1.3394826566** | **0.1** | **PTPRF** |
| **GO:0007156** | **homophilic cell adhesion via plasma membrane adhesion molecules** | **1** | **21.20727273** | **0.0461742733** | **1.3355999305** | **0.1** | **PTPRF** |
| **GO:0016241** | **regulation of macroautophagy** | **1** | **21.01621622** | **0.0465850864** | **1.3317530948** | **0.1** | **TBC1D14** |
| **GO:0038127** | **ERBB signaling pathway** | **1** | **20.82857143** | **0.0469957402** | **1.3279415059** | **0.1** | **PTPRF** |
| **GO:0006888** | **endoplasmic reticulum to Golgi vesicle-mediated transport** | **1** | **20.28521739** | **0.0482267466** | **1.3167120347** | **0.1** | **SEC24D** |
| **GO:0051649** | **establishment of localization in cell** | **3** | **3.481791045** | **0.0483819209** | **1.3153168926** | **0.3** | **TBC1D14//SEC24D//TRAPPC10** |
| **GO:0048024** | **regulation of mRNA splicing, via spliceosome** | **1** | **20.11034483** | **0.0486367639** | **1.3130353286** | **0.1** | **KHDRBS2** |
| **GO:0048814** | **regulation of dendrite morphogenesis** | **1** | **19.93846154** | **0.0490466223** | **1.3093908957** | **0.1** | **PTPRF** |
| **GO:0060759** | **regulation of response to cytokine stimulus** | **1** | **19.60336134** | **0.0498658623** | **1.3021966663** | **0.1** | **PTPRF** |
| **Cellular component** | | | | | | | |
| **GO.ID** | **Term** | **Count** | **Fold.Enrichment** | **Pvalue** | **Enrichment.Score** | **Gene.Ratio** | **GENES** |
| **GO:0031982** | **vesicle** | **6** | **6.690273119** | **7.8962E-05** | **4.102581595** | **0.6** | **PDIA3//PTPRF//SEC24D//TBC1D14//ALB//TRAPPC10** |
| **GO:0031410** | **cytoplasmic vesicle** | **5** | **6.000773595** | **0.000704629** | **3.152039395** | **0.5** | **PDIA3//PTPRF//SEC24D//TBC1D14//TRAPPC10** |
| **GO:0097708** | **intracellular vesicle** | **5** | **5.979188078** | **0.000716497** | **3.144785802** | **0.5** | **PDIA3//PTPRF//SEC24D//TBC1D14//TRAPPC10** |
| **GO:0043229** | **intracellular organelle** | **10** | **1.881853469** | **0.00179226** | **2.746598921** | **1** | **PDIA3//KHDRBS2//FOXN3//TBC1D14//PTPRF//ALB//TRAPPC10//TTLL8//EML5//SEC24D** |
| **GO:0043226** | **organelle** | **10** | **1.829193523** | **0.002380712** | **2.623293184** | **1** | **PDIA3//KHDRBS2//FOXN3//TBC1D14//PTPRF//ALB//TRAPPC10//TTLL8//EML5//SEC24D** |
| **GO:0012505** | **endomembrane system** | **6** | **3.553728684** | **0.002606264** | **2.583981656** | **0.6** | **PDIA3//PTPRF//ALB//TBC1D14//TRAPPC10//SEC24D** |
| **GO:0043209** | **myelin sheath** | **2** | **21.85070423** | **0.003575226** | **2.446696503** | **0.2** | **ALB//PDIA3** |
| **GO:0005768** | **endosome** | **3** | **7.782943144** | **0.005589293** | **2.252643091** | **0.3** | **TBC1D14//TRAPPC10//PTPRF** |
| **GO:0030127** | **COPII vesicle coat** | **1** | **166.2214286** | **0.006000968** | **2.221778683** | **0.1** | **SEC24D** |
| **GO:0030008** | **TRAPP complex** | **1** | **155.14** | **0.006428366** | **2.191899413** | **0.1** | **TRAPPC10** |
| **GO:0042824** | **MHC class I peptide loading complex** | **1** | **155.14** | **0.006428366** | **2.191899413** | **0.1** | **PDIA3** |
| **GO:0005622** | **intracellular** | **10** | **1.647854412** | **0.006764993** | **2.169732662** | **1** | **PDIA3//KHDRBS2//FOXN3//TBC1D14//ALB//TTLL8//EML5//PTPRF//TRAPPC10//SEC24D** |
| **GO:0012507** | **ER to Golgi transport vesicle membrane** | **1** | **110.8142857** | **0.008989281** | **2.046275036** | **0.1** | **SEC24D** |
| **GO:0070971** | **endoplasmic reticulum exit site** | **1** | **72.721875** | **0.013668868** | **1.864267445** | **0.1** | **SEC24D** |
| **GO:0005874** | **microtubule** | **2** | **10.82372093** | **0.013896013** | **1.857109774** | **0.2** | **TTLL8//EML5** |
| **GO:0005790** | **smooth endoplasmic reticulum** | **1** | **64.64166667** | **0.015365599** | **1.813450502** | **0.1** | **PDIA3** |
| **GO:0005794** | **Golgi apparatus** | **3** | **4.824671735** | **0.020694676** | **1.684141367** | **0.3** | **TRAPPC10//TBC1D14//ALB** |
| **GO:0030120** | **vesicle coat** | **1** | **42.31090909** | **0.023389253** | **1.630983652** | **0.1** | **SEC24D** |
| **GO:0030134** | **COPII-coated ER to Golgi transport vesicle** | **1** | **42.31090909** | **0.023389253** | **1.630983652** | **0.1** | **SEC24D** |
| **GO:0030660** | **Golgi-associated vesicle membrane** | **1** | **38.14918033** | **0.025910784** | **1.586519438** | **0.1** | **SEC24D** |
| **GO:0099023** | **vesicle tethering complex** | **1** | **34.22205882** | **0.028845165** | **1.539926976** | **0.1** | **TRAPPC10** |
| **GO:0005783** | **endoplasmic reticulum** | **3** | **4.21576087** | **0.029558301** | **1.529320531** | **0.3** | **PDIA3//SEC24D//ALB** |
| **GO:0099061** | **integral component of postsynaptic density membrane** | **1** | **32.77605634** | **0.030100319** | **1.521428901** | **0.1** | **PTPRF** |
| **GO:0005776** | **autophagosome** | **1** | **31.87808219** | **0.030936277** | **1.509531948** | **0.1** | **TBC1D14** |
| **GO:0043231** | **intracellular membrane-bounded organelle** | **8** | **1.742493448** | **0.031278506** | **1.504753997** | **0.8** | **PDIA3//KHDRBS2//FOXN3//TBC1D14//ALB//TRAPPC10//SEC24D//PTPRF** |
| **GO:0060076** | **excitatory synapse** | **1** | **30.22207792** | **0.032606249** | **1.486699164** | **0.1** | **PTPRF** |
| **GO:0099146** | **intrinsic component of postsynaptic density membrane** | **1** | **30.22207792** | **0.032606249** | **1.486699164** | **0.1** | **PTPRF** |
| **GO:0030662** | **coated vesicle membrane** | **1** | **29.83461538** | **0.033023337** | **1.481179049** | **0.1** | **SEC24D** |
| **GO:0099513** | **polymeric cytoskeletal fiber** | **2** | **6.745217391** | **0.033737458** | **1.471887638** | **0.2** | **TTLL8//EML5** |
| **GO:0005758** | **mitochondrial intermembrane space** | **1** | **28.0373494** | **0.03510635** | **1.454614326** | **0.1** | **PDIA3** |
| **GO:0005788** | **endoplasmic reticulum lumen** | **1** | **27.37764706** | **0.035938423** | **1.444440979** | **0.1** | **PDIA3** |
| **GO:0070062** | **extracellular exosome** | **1** | **25.29456522** | **0.038845599** | **1.41065818** | **0.1** | **ALB** |
| **GO:0031970** | **organelle envelope lumen** | **1** | **24.240625** | **0.040503297** | **1.392509623** | **0.1** | **PDIA3** |
| **GO:0030117** | **membrane coat** | **1** | **23.99072165** | **0.040917319** | **1.388092826** | **0.1** | **SEC24D** |
| **GO:0048475** | **coated membrane** | **1** | **23.99072165** | **0.040917319** | **1.388092826** | **0.1** | **SEC24D** |
| **GO:0098839** | **postsynaptic density membrane** | **1** | **23.99072165** | **0.040917319** | **1.388092826** | **0.1** | **PTPRF** |
| **GO:0005737** | **cytoplasm** | **8** | **1.67086699** | **0.041253615** | **1.38453799** | **0.8** | **PDIA3//PTPRF//TBC1D14//ALB//TRAPPC10//TTLL8//SEC24D//EML5** |
| **GO:1903561** | **extracellular vesicle** | **1** | **23.271** | **0.042158422** | **1.375115656** | **0.1** | **ALB** |
| **GO:0099060** | **integral component of postsynaptic specialization membrane** | **1** | **22.16285714** | **0.044223714** | **1.354344788** | **0.1** | **PTPRF** |
| **GO:0043230** | **extracellular organelle** | **1** | **21.15545455** | **0.046284997** | **1.334559756** | **0.1** | **ALB** |
| **GO:0098948** | **intrinsic component of postsynaptic specialization membrane** | **1** | **20.96486486** | **0.046696774** | **1.330713124** | **0.1** | **PTPRF** |
| **GO:0005604** | **basement membrane** | **1** | **20.41315789** | **0.047931143** | **1.319382218** | **0.1** | **ALB** |
| **GO:0043227** | **membrane-bounded organelle** | **8** | **1.624219159** | **0.049601612** | **1.304504208** | **0.8** | **PDIA3//KHDRBS2//FOXN3//TBC1D14//PTPRF//ALB//TRAPPC10//SEC24D** |
| Molecular function | | | | | | | |
| GO.ID | Term | Count | Fold.Enrichment | Pvalue | Enrichment.Score | Gene.Ratio | GENES |
| **GO:0016671** | **oxidoreductase activity, acting on a sulfur group of donors, disulfide as acceptor** | **1** | **206.0818182** | **0.004842819** | **2.314901772** | **0.1** | **PDIA3** |
| **GO:0015037** | **peptide disulfide oxidoreductase activity** | **1** | **188.9083333** | **0.005282027** | **2.277199403** | **0.1** | **PDIA3** |
| **GO:0042301** | **phosphate ion binding** | **1** | **174.3769231** | **0.00572106** | **2.242523485** | **0.1** | **PTPRF** |
| **GO:0015643** | **toxic substance binding** | **1** | **161.9214286** | **0.006159919** | **2.210424988** | **0.1** | **ALB** |
| **GO:0003756** | **protein disulfide isomerase activity** | **1** | **133.3470588** | **0.00747545** | **2.12636264** | **0.1** | **PDIA3** |
| **GO:0016864** | **intramolecular oxidoreductase activity, transposing S-S bonds** | **1** | **133.3470588** | **0.00747545** | **2.12636264** | **0.1** | **PDIA3** |
| **GO:0042288** | **MHC class I protein binding** | **1** | **119.3105263** | **0.0083516** | **2.078230303** | **0.1** | **PDIA3** |
| **GO:0016881** | **acid-amino acid ligase activity** | **1** | **103.0409091** | **0.00966452** | **2.014819713** | **0.1** | **TTLL8** |
| **GO:0019825** | **oxygen binding** | **1** | **87.18846154** | **0.011412646** | **1.942613655** | **0.1** | **ALB** |
| **GO:0005158** | **insulin receptor binding** | **1** | **80.96071429** | **0.012285667** | **1.910601256** | **0.1** | **PTPRF** |
| **GO:0008143** | **poly(A) binding** | **1** | **80.96071429** | **0.012285667** | **1.910601256** | **0.1** | **KHDRBS2** |
| **GO:0008266** | **poly(U) RNA binding** | **1** | **80.96071429** | **0.012285667** | **1.910601256** | **0.1** | **KHDRBS2** |
| **GO:0008187** | **poly-pyrimidine tract binding** | **1** | **70.840625** | **0.014029628** | **1.852953842** | **0.1** | **KHDRBS2** |
| **GO:0042287** | **MHC protein binding** | **1** | **62.96944444** | **0.015770817** | **1.802145801** | **0.1** | **PDIA3** |
| **GO:0070717** | **poly-purine tract binding** | **1** | **62.96944444** | **0.015770817** | **1.802145801** | **0.1** | **KHDRBS2** |
| **GO:0043394** | **proteoglycan binding** | **1** | **59.65526316** | **0.016640374** | **1.778836926** | **0.1** | **PTPRF** |
| **GO:0015036** | **disulfide oxidoreductase activity** | **1** | **55.2902439** | **0.017943412** | **1.746094975** | **0.1** | **PDIA3** |
| **GO:0042169** | **SH2 domain binding** | **1** | **51.52045455** | **0.019244896** | **1.715684435** | **0.1** | **KHDRBS2** |
| **GO:0016879** | **ligase activity, forming carbon-nitrogen bonds** | **1** | **46.26326531** | **0.021410587** | **1.669371433** | **0.1** | **TTLL8** |
| **GO:0016860** | **intramolecular oxidoreductase activity** | **1** | **45.338** | **0.021843208** | **1.66068358** | **0.1** | **PDIA3** |
| **GO:0005504** | **fatty acid binding** | **1** | **43.59423077** | **0.022707934** | **1.643822372** | **0.1** | **ALB** |
| **GO:0030170** | **pyridoxal phosphate binding** | **1** | **43.59423077** | **0.022707934** | **1.643822372** | **0.1** | **ALB** |
| **GO:0070279** | **vitamin B6 binding** | **1** | **42.77169811** | **0.023140039** | **1.635635907** | **0.1** | **ALB** |
| **GO:0016667** | **oxidoreductase activity, acting on a sulfur group of donors** | **1** | **40.48035714** | **0.024435323** | **1.611981915** | **0.1** | **PDIA3** |
| **GO:0030971** | **receptor tyrosine kinase binding** | **1** | **31.05342466** | **0.031746111** | **1.498309468** | **0.1** | **PTPRF** |
| **GO:0008270** | **zinc ion binding** | **2** | **6.63806735** | **0.034746363** | **1.459090648** | **0.2** | **ALB//SEC24D** |
| **GO:0033293** | **monocarboxylic acid binding** | **1** | **27.64512195** | **0.035596533** | **1.448592305** | **0.1** | **ALB** |
| **GO:0003727** | **single-stranded RNA binding** | **1** | **24.37526882** | **0.040283886** | **1.394868646** | **0.1** | **KHDRBS2** |
| **GO:0019901** | **protein kinase binding** | **2** | **6.069344043** | **0.040946089** | **1.387787572** | **0.2** | **PTPRF//TBC1D14** |
| **GO:0004725** | **protein tyrosine phosphatase activity** | **1** | **23.13163265** | **0.042407714** | **1.372555133** | **0.1** | **PTPRF** |
| **GO:0051087** | **chaperone binding** | **1** | **22.2245098** | **0.044103731** | **1.355524671** | **0.1** | **ALB** |
| **GO:1990782** | **protein tyrosine kinase binding** | **1** | **21.58952381** | **0.045373968** | **1.343193236** | **0.1** | **PTPRF** |
| **GO:0019900** | **kinase binding** | **2** | **5.449278846** | **0.049793884** | **1.302823998** | **0.2** | **TBC1D14//PTPRF** |

## 1.3 Supplementary Table3

The UP-regulated circRNAs identified in HFD-fed mice vs control were categorized using GO annotation.

| **Biologic process** | | | | | | | | |
| --- | --- | --- | --- | --- | --- | --- | --- | --- |
| **GO.ID** | **Term** | **Count** | | **Fold.Enrichment** | **Pvalue** | **Enrichment.Score** | **Gene.Ratio** | **GENES** |
| **GO:0045184** | **establishment of protein localization** | **11** | **3.621184539** | | **0.000134796** | **3.870321685** | **0.282051282** | **NCBP1//TSC2//IPO11//VTI1A//SEC23B//AHCTF1//CACNA1E//RAB11FIP3//STAU2//MAP1A//RAP2A** |
| **GO:0008104** | **protein localization** | **13** | **2.995377504** | | **0.00019311** | **3.714195972** | **0.333333333** | **NCBP1//TSC2//IPO11//VTI1A//SEC23B//AHCTF1//TRIM21//RAP2A//CACNA1E//RAB11FIP3//STAU2//MAP1A//CAMK2D** |
| **GO:0007610** | **behavior** | **7** | **5.650576145** | | **0.000200988** | **3.696829579** | **0.179487179** | **CACNA1E//MAP1A//FXN//TSC2//LSAMP//CFAP69//NCOA2** |
| **GO:0022604** | **regulation of cell morphogenesis** | **6** | **6.670860738** | | **0.000249998** | **3.602062919** | **0.153846154** | **PTK2//FMNL2//FXN//RAP2A//TSC2//STAU2** |
| **GO:0010769** | **regulation of cell morphogenesis involved in differentiation** | **5** | **8.520710059** | | **0.000283448** | **3.547526558** | **0.128205128** | **FXN//RAP2A//PTK2//TSC2//STAU2** |
| **GO:0051649** | **establishment of localization in cell** | **11** | **3.27347876** | | **0.000327576** | **3.484688352** | **0.282051282** | **NCBP1//TSC2//IPO11//VTI1A//SEC23B//PTK2//CAMK2D//RAB11FIP3//CACNA1E//STAU2//MAP1A** |
| **GO:0099640** | **axo-dendritic protein transport** | **2** | **70.37104072** | | **0.000364555** | **3.438236918** | **0.051282051** | **STAU2//MAP1A** |
| **GO:0015031** | **protein transport** | **10** | **3.491849656** | | **0.000392547** | **3.406108507** | **0.256410256** | **NCBP1//TSC2//IPO11//VTI1A//SEC23B//CACNA1E//RAB11FIP3//STAU2//MAP1A//AHCTF1** |
| **GO:0046907** | **intracellular transport** | **9** | **3.83705247** | | **0.000411181** | **3.38596719** | **0.230769231** | **NCBP1//TSC2//IPO11//VTI1A//SEC23B//PTK2//RAB11FIP3//STAU2//MAP1A** |
| **GO:0015833** | **peptide transport** | **10** | **3.383223112** | | **0.000504804** | **3.296877328** | **0.256410256** | **NCBP1//TSC2//IPO11//VTI1A//SEC23B//AHCTF1//CACNA1E//RAB11FIP3//STAU2//MAP1A** |
| **GO:0010763** | **positive regulation of fibroblast migration** | **2** | **59.81538462** | | **0.000507693** | **3.294398502** | **0.051282051** | **PTK2//TSC2** |
| **GO:0065008** | **regulation of biological quality** | **16** | **2.331983806** | | **0.000507988** | **3.294146185** | **0.41025641** | **CACNA1E//CAMK2D//FXN//PTK2//FMNL2//CNNM2//TRIM21//PDIA5//MAP1A//IQCB1//TSC2//NCOA2//SVIL//STAU2//GRHL1//RAB11FIP3** |
| **GO:0034613** | **cellular protein localization** | **10** | **3.347251517** | | **0.000549354** | **3.260147785** | **0.256410256** | **NCBP1//TSC2//IPO11//VTI1A//SEC23B//RAB11FIP3//RAP2A//STAU2//MAP1A//CAMK2D** |
| **GO:0042886** | **amide transport** | **10** | **3.334190893** | | **0.000566576** | **3.24674201** | **0.256410256** | **NCBP1//TSC2//IPO11//VTI1A//SEC23B//AHCTF1//CACNA1E//RAB11FIP3//STAU2//MAP1A** |
| **GO:0070727** | **cellular macromolecule localization** | **10** | **3.326773338** | | **0.000576618** | **3.23911173** | **0.256410256** | **NCBP1//TSC2//IPO11//VTI1A//SEC23B//RAP2A//RAB11FIP3//STAU2//MAP1A//CAMK2D** |
| **GO:0048667** | **cell morphogenesis involved in neuron differentiation** | **6** | **5.547021757** | | **0.000665194** | **3.177051575** | **0.153846154** | **MAP1A//PTK2//FXN//RAP2A//TSC2//STAU2** |
| **GO:0033036** | **macromolecule localization** | **13** | **2.623481781** | | **0.000714096** | **3.146243423** | **0.333333333** | **NCBP1//TSC2//IPO11//VTI1A//SEC23B//AHCTF1//TRIM21//RAP2A//CACNA1E//RAB11FIP3//STAU2//MAP1A//CAMK2D** |
| **GO:0006886** | **intracellular protein transport** | **7** | **4.492571806** | | **0.000797742** | **3.098137597** | **0.179487179** | **NCBP1//TSC2//IPO11//VTI1A//STAU2//MAP1A//SEC23B** |
| **GO:0007626** | **locomotory behavior** | **4** | **9.273703041** | | **0.000886828** | **3.052160676** | **0.102564103** | **FXN//LSAMP//NCOA2//CACNA1E** |
| **GO:0022607** | **cellular component assembly** | **12** | **2.695398481** | | **0.000968078** | **3.01408981** | **0.307692308** | **NCBP1//DHX29//PTK2//CFAP69//FXN//RAP2A//SVIL//TMEM120B//AHCTF1//STAU2//IQCB1//TEAD2** |
| **GO:0048812** | **neuron projection morphogenesis** | **6** | **5.141723606** | | **0.000987073** | **3.005650582** | **0.153846154** | **MAP1A//PTK2//FXN//RAP2A//TSC2//STAU2** |
| **GO:0120039** | **plasma membrane bounded cell projection morphogenesis** | **6** | **5.040622299** | | **0.001093593** | **2.961144366** | **0.153846154** | **MAP1A//PTK2//FXN//RAP2A//TSC2//STAU2** |
| **GO:0048858** | **cell projection morphogenesis** | **6** | **5.005471516** | | **0.001133712** | **2.945497432** | **0.153846154** | **MAP1A//PTK2//FXN//RAP2A//TSC2//STAU2** |
| **GO:0043276** | **anoikis** | **2** | **38.59057072** | | **0.001228169** | **2.910741922** | **0.051282051** | **PTK2//TSC2** |
| **GO:0051641** | **cellular localization** | **12** | **2.616786786** | | **0.001258715** | **2.900072526** | **0.307692308** | **NCBP1//TSC2//IPO11//VTI1A//SEC23B//PTK2//CAMK2D//RAB11FIP3//RAP2A//CACNA1E//STAU2//MAP1A** |
| **GO:0032990** | **cell part morphogenesis** | **6** | **4.84989605** | | **0.001333094** | **2.875139136** | **0.153846154** | **MAP1A//PTK2//FXN//RAP2A//TSC2//STAU2** |
| **GO:0016358** | **dendrite development** | **4** | **7.819004525** | | **0.001663117** | **2.779077295** | **0.102564103** | **RAP2A//TSC2//STAU2//MAP1A** |
| **GO:0016043** | **cellular component organization** | **19** | **1.881609781** | | **0.00181413** | **2.741331711** | **0.487179487** | **PTK2//MAP1A//NCBP1//DHX29//GRHL1//FXN//SVIL//FMNL2//CDC27//CFAP69//TSC2//VTI1A//IQCB1//RAP2A//STAU2//TMEM120B//AHCTF1//TEAD2//SEC23B** |
| **GO:0006996** | **organelle organization** | **14** | **2.255360583** | | **0.001883413** | **2.725054328** | **0.358974359** | **PTK2//MAP1A//FXN//SVIL//FMNL2//CDC27//CFAP69//VTI1A//RAP2A//STAU2//DHX29//AHCTF1//IQCB1//SEC23B** |
| **GO:0006928** | **movement of cell or subcellular component** | **10** | **2.846995936** | | **0.001912819** | **2.718326049** | **0.256410256** | **PTK2//FKTN//CFAP69//TSC2//CAMK2D//FMNL2//CACNA1E//RAP2A//STAU2//MAP1A** |
| **GO:0010613** | **positive regulation of cardiac muscle hypertrophy** | **2** | **30.67455621** | | **0.001940704** | **2.712040593** | **0.051282051** | **CAMK2D//PTK2** |
| **GO:0051179** | **localization** | **19** | **1.870461336** | | **0.001955769** | **2.70868253** | **0.487179487** | **PTK2//FKTN//CAMK2D//NCBP1//TSC2//IPO11//VTI1A//CNNM2//CACNA1E//FXN//SEC23B//CFAP69//AHCTF1//RAB11FIP3//FMNL2//RAP2A//TRIM21//STAU2//MAP1A** |
| **GO:0071705** | **nitrogen compound transport** | **10** | **2.83619652** | | **0.00196811** | **2.705950703** | **0.256410256** | **NCBP1//TSC2//IPO11//VTI1A//SEC23B//AHCTF1//CACNA1E//RAB11FIP3//STAU2//MAP1A** |
| **GO:0014742** | **positive regulation of muscle hypertrophy** | **2** | **29.90769231** | | **0.002040695** | **2.690221979** | **0.051282051** | **CAMK2D//PTK2** |
| **GO:0044085** | **cellular component biogenesis** | **12** | **2.471710108** | | **0.002069404** | **2.684154677** | **0.307692308** | **NCBP1//DHX29//PTK2//CFAP69//FXN//RAP2A//SVIL//TMEM120B//AHCTF1//STAU2//IQCB1//TEAD2** |
| **GO:0000902** | **cell morphogenesis** | **7** | **3.782363977** | | **0.002156519** | **2.666246684** | **0.179487179** | **MAP1A//PTK2//FMNL2//FXN//RAP2A//TSC2//STAU2** |
| **GO:0000904** | **cell morphogenesis involved in differentiation** | **6** | **4.408996409** | | **0.002161506** | **2.665243635** | **0.153846154** | **MAP1A//PTK2//FXN//RAP2A//TSC2//STAU2** |
| **GO:0010762** | **regulation of fibroblast migration** | **2** | **28.48351648** | | **0.00224787** | **2.64822885** | **0.051282051** | **PTK2//TSC2** |
| **GO:0050657** | **nucleic acid transport** | **3** | **11.42969133** | | **0.002288117** | **2.640521692** | **0.076923077** | **NCBP1//AHCTF1//STAU2** |
| **GO:0050658** | **RNA transport** | **3** | **11.42969133** | | **0.002288117** | **2.640521692** | **0.076923077** | **NCBP1//AHCTF1//STAU2** |
| **GO:0051236** | **establishment of RNA localization** | **3** | **11.21538462** | | **0.002414338** | **2.617201864** | **0.076923077** | **NCBP1//AHCTF1//STAU2** |
| **GO:0032989** | **cellular component morphogenesis** | **6** | **4.298111469** | | **0.00245576** | **2.609814163** | **0.153846154** | **MAP1A//PTK2//FXN//RAP2A//TSC2//STAU2** |
| **GO:0046621** | **negative regulation of organ growth** | **2** | **27.18881119** | | **0.002464586** | **2.608256098** | **0.051282051** | **PTK2//FXN** |
| **GO:0045494** | **photoreceptor cell maintenance** | **2** | **26.58461538** | | **0.002576502** | **2.588969585** | **0.051282051** | **MAP1A//IQCB1** |
| **GO:0071840** | **cellular component organization or biogenesis** | **19** | **1.824518073** | | **0.002670412** | **2.573421692** | **0.487179487** | **PTK2//MAP1A//NCBP1//DHX29//GRHL1//FXN//SVIL//FMNL2//CDC27//CFAP69//TSC2//VTI1A//IQCB1//RAP2A//STAU2//TMEM120B//AHCTF1//TEAD2//SEC23B** |
| **GO:0006403** | **RNA localization** | **3** | **9.859678783** | | **0.003472679** | **2.459335328** | **0.076923077** | **NCBP1//AHCTF1//STAU2** |
| **GO:0010975** | **regulation of neuron projection development** | **5** | **4.800592666** | | **0.003633414** | **2.439685151** | **0.128205128** | **TSC2//FXN//RAP2A//PTK2//STAU2** |
| **GO:0007018** | **microtubule-based movement** | **4** | **6.214585415** | | **0.003807832** | **2.419322255** | **0.102564103** | **CFAP69//CACNA1E//STAU2//MAP1A** |
| **GO:0001895** | **retina homeostasis** | **2** | **21.36263736** | | **0.003961741** | **2.40211396** | **0.051282051** | **MAP1A//IQCB1** |
| **GO:0043933** | **protein-containing complex subunit organization** | **8** | **3.030545136** | | **0.004030733** | **2.394615924** | **0.205128205** | **NCBP1//DHX29//MAP1A//PTK2//SVIL//TMEM120B//AHCTF1//TEAD2** |
| **GO:0120036** | **plasma membrane bounded cell projection organization** | **8** | **3.026711429** | | **0.004061922** | **2.391268405** | **0.205128205** | **CFAP69//MAP1A//TSC2//PTK2//RAP2A//FXN//STAU2//IQCB1** |
| **GO:0015931** | **nucleobase-containing compound transport** | **3** | **9.297728179** | | **0.004093056** | **2.387952336** | **0.076923077** | **NCBP1//AHCTF1//STAU2** |
| **GO:0050879** | **multicellular organismal movement** | **2** | **20.98785425** | | **0.004101486** | **2.387058775** | **0.051282051** | **MAP1A//VTI1A** |
| **GO:0050881** | **musculoskeletal movement** | **2** | **20.98785425** | | **0.004101486** | **2.387058775** | **0.051282051** | **MAP1A//VTI1A** |
| **GO:0010761** | **fibroblast migration** | **2** | **20.62599469** | | **0.004243501** | **2.372275742** | **0.051282051** | **PTK2//TSC2** |
| **GO:0042592** | **homeostatic process** | **9** | **2.745224179** | | **0.004299181** | **2.366614246** | **0.230769231** | **CAMK2D//FXN//CNNM2//PTK2//CACNA1E//PDIA5//MAP1A//IQCB1//GRHL1** |
| **GO:0050773** | **regulation of dendrite development** | **3** | **9.10894182** | | **0.004334294** | **2.363081665** | **0.076923077** | **RAP2A//TSC2//STAU2** |
| **GO:0030307** | **positive regulation of cell growth** | **3** | **9.062937063** | | **0.004395926** | **2.356949608** | **0.076923077** | **FXN//PTK2//NCBP1** |
| **GO:0007254** | **JNK cascade** | **3** | **8.972307692** | | **0.004520789** | **2.344785802** | **0.076923077** | **RAP2A//FKTN//PTK2** |
| **GO:0019725** | **cellular homeostasis** | **6** | **3.750180854** | | **0.00480587** | **2.318227971** | **0.153846154** | **FXN//CAMK2D//PTK2//PDIA5//CACNA1E//MAP1A** |
| **GO:0014911** | **positive regulation of smooth muscle cell migration** | **2** | **19.29528536** | | **0.0048341** | **2.31568433** | **0.051282051** | **CAMK2D//PTK2** |
| **GO:0030030** | **cell projection organization** | **8** | **2.939330939** | | **0.004850773** | **2.314189015** | **0.205128205** | **CFAP69//MAP1A//TSC2//PTK2//RAP2A//FXN//STAU2//IQCB1** |
| **GO:0051234** | **establishment of localization** | **15** | **1.962876327** | | **0.004922682** | **2.307798181** | **0.384615385** | **CAMK2D//NCBP1//TSC2//IPO11//VTI1A//CNNM2//CACNA1E//FXN//SEC23B//PTK2//AHCTF1//RAB11FIP3//RAP2A//STAU2//MAP1A** |
| **GO:0035265** | **organ growth** | **3** | **8.668896321** | | **0.004974721** | **2.30323125** | **0.076923077** | **PTK2//FXN//CAMK2D** |
| **GO:0060999** | **positive regulation of dendritic spine development** | **2** | **18.98901099** | | **0.004987348** | **2.302130313** | **0.051282051** | **STAU2//TSC2** |
| **GO:0050770** | **regulation of axonogenesis** | **3** | **8.627218935** | | **0.005041734** | **2.29742005** | **0.076923077** | **FXN//PTK2//TSC2** |
| **GO:0051726** | **regulation of cell cycle** | **6** | **3.703738985** | | **0.005104533** | **2.29204401** | **0.153846154** | **CDC27//CAMK2D//RAB11FIP3//AHCTF1//TRIM21//TSC2** |
| **GO:0098840** | **protein transport along microtubule** | **2** | **18.40473373** | | **0.005300508** | **2.275682474** | **0.051282051** | **STAU2//MAP1A** |
| **GO:0099118** | **microtubule-based protein transport** | **2** | **18.40473373** | | **0.005300508** | **2.275682474** | **0.051282051** | **STAU2//MAP1A** |
| **GO:0006914** | **autophagy** | **4** | **5.629683258** | | **0.005399065** | **2.267681427** | **0.102564103** | **PTK2//TRIM21//TSC2//VTI1A** |
| **GO:0061919** | **process utilizing autophagic mechanism** | **4** | **5.629683258** | | **0.005399065** | **2.267681427** | **0.102564103** | **VTI1A//PTK2//TRIM21//TSC2** |
| **GO:0048870** | **cell motility** | **8** | **2.877468893** | | **0.005511878** | **2.258700379** | **0.205128205** | **PTK2//FKTN//CFAP69//TSC2//CAMK2D//FMNL2//CACNA1E//RAP2A** |
| **GO:0051674** | **localization of cell** | **8** | **2.877468893** | | **0.005511878** | **2.258700379** | **0.205128205** | **PTK2//FKTN//CFAP69//TSC2//CAMK2D//FMNL2//CACNA1E//RAP2A** |
| **GO:0050771** | **negative regulation of axonogenesis** | **2** | **17.59276018** | | **0.005786799** | **2.237561613** | **0.051282051** | **PTK2//TSC2** |
| **GO:0034394** | **protein localization to cell surface** | **2** | **17.33779264** | | **0.005953279** | **2.225243738** | **0.051282051** | **MAP1A//TSC2** |
| **GO:0010611** | **regulation of cardiac muscle hypertrophy** | **2** | **16.61538462** | | **0.006465768** | **2.189379851** | **0.051282051** | **CAMK2D//PTK2** |
| **GO:0007010** | **cytoskeleton organization** | **7** | **3.065210046** | | **0.00686731** | **2.163213371** | **0.179487179** | **PTK2//MAP1A//CFAP69//SVIL//FMNL2//RAP2A//STAU2** |
| **GO:0071702** | **organic substance transport** | **10** | **2.384032866** | | **0.006926037** | **2.159515212** | **0.256410256** | **NCBP1//TSC2//IPO11//VTI1A//SEC23B//AHCTF1//CACNA1E//RAB11FIP3//STAU2//MAP1A** |
| **GO:0010507** | **negative regulation of autophagy** | **2** | **15.95076923** | | **0.006997675** | **2.15504624** | **0.051282051** | **TSC2//PTK2** |
| **GO:0014743** | **regulation of muscle hypertrophy** | **2** | **15.95076923** | | **0.006997675** | **2.15504624** | **0.051282051** | **CAMK2D//PTK2** |
| **GO:0046777** | **protein autophosphorylation** | **3** | **7.4458985** | | **0.007566173** | **2.121123761** | **0.076923077** | **RAP2A//PTK2//CAMK2D** |
| **GO:0030154** | **cell differentiation** | **14** | **1.942959129** | | **0.007647098** | **2.116503331** | **0.358974359** | **PTK2//TEAD2//FKTN//CFAP69//MAP1A//TSC2//TMEM120B//GRHL1//FXN//IQCB1//RAP2A//STAU2//CAMK2D//GMCL1** |
| **GO:0065003** | **protein-containing complex assembly** | **7** | **2.997191785** | | **0.007738478** | **2.111344421** | **0.179487179** | **NCBP1//DHX29//PTK2//SVIL//TMEM120B//AHCTF1//TEAD2** |
| **GO:0008088** | **axo-dendritic transport** | **2** | **14.95384615** | | **0.007926801** | **2.100902072** | **0.051282051** | **STAU2//MAP1A** |
| **GO:0032465** | **regulation of cytokinesis** | **2** | **14.95384615** | | **0.007926801** | **2.100902072** | **0.051282051** | **RAB11FIP3//AHCTF1** |
| **GO:1901880** | **negative regulation of protein depolymerization** | **2** | **14.95384615** | | **0.007926801** | **2.100902072** | **0.051282051** | **MAP1A//SVIL** |
| **GO:0051129** | **negative regulation of cellular component organization** | **5** | **3.956043956** | | **0.008158936** | **2.088366487** | **0.128205128** | **MAP1A//TSC2//PTK2//SVIL//FXN** |
| **GO:0007409** | **axonogenesis** | **4** | **4.963932333** | | **0.008359839** | **2.077802077** | **0.102564103** | **PTK2//FXN//TSC2//MAP1A** |
| **GO:0031330** | **negative regulation of cellular catabolic process** | **3** | **7.177846154** | | **0.00836306** | **2.07763477** | **0.076923077** | **PTK2//TSC2//MAP1A** |
| **GO:0000165** | **MAPK cascade** | **5** | **3.894230769** | | **0.008701646** | **2.060398612** | **0.128205128** | **PTK2//TSC2//RAP2A//FKTN//CAMK2D** |
| **GO:0014910** | **regulation of smooth muscle cell migration** | **2** | **14.07420814** | | **0.008908413** | **2.050199672** | **0.051282051** | **CAMK2D//PTK2** |
| **GO:0051403** | **stress-activated MAPK cascade** | **3** | **6.982340617** | | **0.009015871** | **2.044992332** | **0.076923077** | **PTK2//RAP2A//FKTN** |
| **GO:0010646** | **regulation of cell communication** | **12** | **2.067352003** | | **0.009043492** | **2.043663846** | **0.307692308** | **PTK2//CAMK2D//TSC2//TRIM21//RAP2A//FKTN//MAP1A//CACNA1E//RAB11FIP3//FXN//STAU2//NCOA2** |
| **GO:0023014** | **signal transduction by protein phosphorylation** | **5** | **3.844176389** | | **0.009172773** | **2.037499362** | **0.128205128** | **PTK2//TSC2//RAP2A//FKTN//CAMK2D** |
| **GO:0023051** | **regulation of signaling** | **12** | **2.059049384** | | **0.009334272** | **2.029919533** | **0.307692308** | **PTK2//TSC2//CAMK2D//TRIM21//RAP2A//FKTN//MAP1A//CACNA1E//RAB11FIP3//FXN//STAU2//NCOA2** |
| **GO:1901992** | **positive regulation of mitotic cell cycle phase transition** | **2** | **13.59440559** | | **0.009522202** | **2.021262626** | **0.051282051** | **CAMK2D//CDC27** |
| **GO:0048869** | **cellular developmental process** | **14** | **1.895462618** | | **0.009536224** | **2.020623553** | **0.358974359** | **PTK2//TEAD2//FKTN//CFAP69//MAP1A//TSC2//GMCL1//TMEM120B//GRHL1//FXN//IQCB1//RAP2A//STAU2//CAMK2D** |
| **GO:0006810** | **transport** | **14** | **1.889901568** | | **0.00978712** | **2.009345097** | **0.358974359** | **CAMK2D//NCBP1//TSC2//IPO11//VTI1A//CACNA1E//FXN//CNNM2//SEC23B//PTK2//AHCTF1//RAB11FIP3//STAU2//MAP1A** |
| **GO:0022603** | **regulation of anatomical structure morphogenesis** | **6** | **3.22744881** | | **0.009807729** | **2.008431526** | **0.153846154** | **PTK2//FMNL2//FXN//RAP2A//TSC2//STAU2** |
| **GO:0030029** | **actin filament-based process** | **5** | **3.771461829** | | **0.009912361** | **2.00382289** | **0.128205128** | **SVIL//FMNL2//RAP2A//STAU2//CAMK2D** |
| **GO:0045664** | **regulation of neuron differentiation** | **5** | **3.771461829** | | **0.009912361** | **2.00382289** | **0.128205128** | **TSC2//FXN//RAP2A//PTK2//STAU2** |
| **GO:0043242** | **negative regulation of protein-containing complex disassembly** | **2** | **13.29230769** | | **0.009941618** | **2.002542907** | **0.051282051** | **MAP1A//SVIL** |
| **GO:0120031** | **plasma membrane bounded cell projection assembly** | **4** | **4.709872804** | | **0.010007104** | **1.999691598** | **0.102564103** | **CFAP69//RAP2A//STAU2//IQCB1** |
| **GO:0031175** | **neuron projection development** | **6** | **3.210127976** | | **0.010056236** | **1.997564543** | **0.153846154** | **MAP1A//TSC2//PTK2//FXN//RAP2A//STAU2** |
| **GO:0120035** | **regulation of plasma membrane bounded cell projection organization** | **5** | **3.747831116** | | **0.01016782** | **1.992772165** | **0.128205128** | **TSC2//FXN//RAP2A//PTK2//STAU2** |
| **GO:0014909** | **smooth muscle cell migration** | **2** | **13.00334448** | | **0.010369149** | **1.984256884** | **0.051282051** | **CAMK2D//PTK2** |
| **GO:0031098** | **stress-activated protein kinase signaling cascade** | **3** | **6.597285068** | | **0.010513417** | **1.978256104** | **0.076923077** | **PTK2//RAP2A//FKTN** |
| **GO:0031344** | **regulation of cell projection organization** | **5** | **3.706033743** | | **0.010639085** | **1.973095733** | **0.128205128** | **TSC2//FXN//RAP2A//PTK2//STAU2** |
| **GO:0040011** | **locomotion** | **8** | **2.568561873** | | **0.01072368** | **1.969656151** | **0.205128205** | **PTK2//FKTN//CFAP69//TSC2//CAMK2D//FMNL2//CACNA1E//RAP2A** |
| **GO:1901879** | **regulation of protein depolymerization** | **2** | **12.72667758** | | **0.010804737** | **1.96638579** | **0.051282051** | **MAP1A//SVIL** |
| **GO:0061564** | **axon development** | **4** | **4.583554377** | | **0.010975988** | **1.959556392** | **0.102564103** | **MAP1A//PTK2//FXN//TSC2** |
| **GO:0050807** | **regulation of synapse organization** | **3** | **6.4782005** | | **0.011042836** | **1.956919379** | **0.076923077** | **PTK2//STAU2//TSC2** |
| **GO:0016049** | **cell growth** | **4** | **4.57479041** | | **0.011047439** | **1.956738402** | **0.102564103** | **PTK2//FXN//NCBP1//CAMK2D** |
| **GO:0030031** | **cell projection assembly** | **4** | **4.57479041** | | **0.011047439** | **1.956738402** | **0.102564103** | **CFAP69//RAP2A//STAU2//IQCB1** |
| **GO:0010506** | **regulation of autophagy** | **3** | **6.45489762** | | **0.011150548** | **1.952703771** | **0.076923077** | **PTK2//TRIM21//TSC2** |
| **GO:0060998** | **regulation of dendritic spine development** | **2** | **12.46153846** | | **0.011248327** | **1.948912051** | **0.051282051** | **TSC2//STAU2** |
| **GO:0001570** | **vasculogenesis** | **2** | **12.33306899** | | **0.011473106** | **1.940318998** | **0.051282051** | **PTK2//TEAD2** |
| **GO:0014066** | **regulation of phosphatidylinositol 3-kinase signaling** | **2** | **12.08391608** | | **0.011928595** | **1.923410704** | **0.051282051** | **TSC2//PTK2** |
| **GO:0043502** | **regulation of muscle adaptation** | **2** | **12.08391608** | | **0.011928595** | **1.923410704** | **0.051282051** | **CAMK2D//PTK2** |
| **GO:0010771** | **negative regulation of cell morphogenesis involved in differentiation** | **2** | **11.96307692** | | **0.012159292** | **1.915091711** | **0.051282051** | **PTK2//TSC2** |
| **GO:0050803** | **regulation of synapse structure or activity** | **3** | **6.209209476** | | **0.012375852** | **1.907424909** | **0.076923077** | **PTK2//STAU2//TSC2** |
| **GO:0035418** | **protein localization to synapse** | **2** | **11.72850679** | | **0.012626556** | **1.89871508** | **0.051282051** | **STAU2//MAP1A** |
| **GO:1901989** | **positive regulation of cell cycle phase transition** | **2** | **11.72850679** | | **0.012626556** | **1.89871508** | **0.051282051** | **CAMK2D//CDC27** |
| **GO:0003300** | **cardiac muscle hypertrophy** | **2** | **11.61463779** | | **0.01286311** | **1.890654017** | **0.051282051** | **CAMK2D//PTK2** |
| **GO:0033365** | **protein localization to organelle** | **5** | **3.526850508** | | **0.012977754** | **1.886800461** | **0.128205128** | **TSC2//IPO11//VTI1A//RAB11FIP3//MAP1A** |
| **GO:0006413** | **translational initiation** | **2** | **11.50295858** | | **0.013101602** | **1.882675589** | **0.051282051** | **DHX29//NCBP1** |
| **GO:0014812** | **muscle cell migration** | **2** | **11.28592163** | | **0.013584375** | **1.866960323** | **0.051282051** | **CAMK2D//PTK2** |
| **GO:0106027** | **neuron projection organization** | **2** | **11.28592163** | | **0.013584375** | **1.866960323** | **0.051282051** | **STAU2//MAP1A** |
| **GO:0014897** | **striated muscle hypertrophy** | **2** | **11.18044572** | | **0.013828643** | **1.85922044** | **0.051282051** | **CAMK2D//PTK2** |
| **GO:0008306** | **associative learning** | **2** | **11.07692308** | | **0.014074822** | **1.851557096** | **0.051282051** | **CACNA1E//MAP1A** |
| **GO:0048661** | **positive regulation of smooth muscle cell proliferation** | **2** | **11.07692308** | | **0.014074822** | **1.851557096** | **0.051282051** | **CAMK2D//PTK2** |
| **GO:0014896** | **muscle hypertrophy** | **2** | **10.97529993** | | **0.014322905** | **1.843968874** | **0.051282051** | **CAMK2D//PTK2** |
| **GO:0051028** | **mRNA transport** | **2** | **10.97529993** | | **0.014322905** | **1.843968874** | **0.051282051** | **NCBP1//AHCTF1** |
| **GO:0051668** | **localization within membrane** | **2** | **10.97529993** | | **0.014322905** | **1.843968874** | **0.051282051** | **TSC2//SEC23B** |
| **GO:1900006** | **positive regulation of dendrite development** | **2** | **10.97529993** | | **0.014322905** | **1.843968874** | **0.051282051** | **TSC2//STAU2** |
| **GO:0006913** | **nucleocytoplasmic transport** | **3** | **5.826173826** | | **0.01466866** | **1.833609566** | **0.076923077** | **NCBP1//TSC2//IPO11** |
| **GO:0051169** | **nuclear transport** | **3** | **5.826173826** | | **0.01466866** | **1.833609566** | **0.076923077** | **NCBP1//TSC2//IPO11** |
| **GO:0045595** | **regulation of cell differentiation** | **8** | **2.427818757** | | **0.014755863** | **1.831035381** | **0.205128205** | **TSC2//GRHL1//PTK2//FXN//IQCB1//RAP2A//STAU2//TEAD2** |
| **GO:0001843** | **neural tube closure** | **2** | **10.77754678** | | **0.01482476** | **1.829012315** | **0.051282051** | **TEAD2//TSC2** |
| **GO:0007017** | **microtubule-based process** | **5** | **3.410227173** | | **0.014829678** | **1.828868292** | **0.128205128** | **PTK2//MAP1A//CFAP69//CACNA1E//STAU2** |
| **GO:0009895** | **negative regulation of catabolic process** | **3** | **5.769972792** | | **0.015051283** | **1.822426484** | **0.076923077** | **PTK2//TSC2//MAP1A** |
| **GO:0060606** | **tube closure** | **2** | **10.68131868** | | **0.015078518** | **1.821641331** | **0.051282051** | **TEAD2//TSC2** |
| **GO:0048878** | **chemical homeostasis** | **6** | **2.934524184** | | **0.015173228** | **1.818922007** | **0.153846154** | **CAMK2D//FXN//CNNM2//PTK2//CACNA1E//GRHL1** |
| **GO:0045927** | **positive regulation of growth** | **3** | **5.75147929** | | **0.015180079** | **1.818725971** | **0.076923077** | **PTK2//FXN//NCBP1** |
| **GO:0090066** | **regulation of anatomical structure size** | **4** | **4.118098769** | | **0.015722958** | **1.803465753** | **0.102564103** | **FXN//TSC2//SVIL//PTK2** |
| **GO:0006888** | **endoplasmic reticulum to Golgi vesicle-mediated transport** | **2** | **10.40267559** | | **0.015851034** | **1.799942411** | **0.051282051** | **SEC23B//VTI1A** |
| **GO:2000147** | **positive regulation of cell motility** | **4** | **4.076005766** | | **0.016268037** | **1.788664854** | **0.102564103** | **PTK2//TSC2//CAMK2D//CFAP69** |
| **GO:0010564** | **regulation of cell cycle process** | **4** | **4.069073783** | | **0.016360033** | **1.786215826** | **0.102564103** | **CDC27//CAMK2D//RAB11FIP3//AHCTF1** |
| **GO:0048814** | **regulation of dendrite morphogenesis** | **2** | **10.22485207** | | **0.016375346** | **1.785809523** | **0.051282051** | **STAU2//RAP2A** |
| **GO:0002934** | **desmosome organization** | **1** | **59.81538462** | | **0.016596071** | **1.779994719** | **0.025641026** | **GRHL1** |
| **GO:0046598** | **positive regulation of viral entry into host cell** | **1** | **59.81538462** | | **0.016596071** | **1.779994719** | **0.025641026** | **TRIM21** |
| **GO:0061517** | **macrophage proliferation** | **1** | **59.81538462** | | **0.016596071** | **1.779994719** | **0.025641026** | **PTK2** |
| **GO:0075294** | **positive regulation by symbiont of entry into host** | **1** | **59.81538462** | | **0.016596071** | **1.779994719** | **0.025641026** | **TRIM21** |
| **GO:1990535** | **neuron projection maintenance** | **1** | **59.81538462** | | **0.016596071** | **1.779994719** | **0.025641026** | **MAP1A** |
| **GO:0014020** | **primary neural tube formation** | **2** | **10.13820078** | | **0.016640272** | **1.778839568** | **0.051282051** | **TEAD2//TSC2** |
| **GO:0030317** | **flagellated sperm motility** | **2** | **10.13820078** | | **0.016640272** | **1.778839568** | **0.051282051** | **CFAP69//CACNA1E** |
| **GO:0050769** | **positive regulation of neurogenesis** | **4** | **4.041580042** | | **0.016731316** | **1.776469906** | **0.102564103** | **TSC2//FXN//PTK2//STAU2** |
| **GO:0046620** | **regulation of organ growth** | **2** | **9.886840432** | | **0.017446057** | **1.758302718** | **0.051282051** | **PTK2//FXN** |
| **GO:0097722** | **sperm motility** | **2** | **9.886840432** | | **0.017446057** | **1.758302718** | **0.051282051** | **CFAP69//CACNA1E** |
| **GO:0040017** | **positive regulation of locomotion** | **4** | **3.967853042** | | **0.017779712** | **1.750075283** | **0.102564103** | **PTK2//TSC2//CAMK2D//CFAP69** |
| **GO:0048666** | **neuron development** | **6** | **2.832614899** | | **0.017782637** | **1.750003838** | **0.153846154** | **MAP1A//TSC2//PTK2//FXN//RAP2A//STAU2** |
| **GO:0051261** | **protein depolymerization** | **2** | **9.726078799** | | **0.017992351** | **1.744912081** | **0.051282051** | **MAP1A//SVIL** |
| **GO:0030644** | **cellular chloride ion homeostasis** | **1** | **54.37762238** | | **0.018240841** | **1.738955139** | **0.025641026** | **PTK2** |
| **GO:0031442** | **positive regulation of mRNA 3'-end processing** | **1** | **54.37762238** | | **0.018240841** | **1.738955139** | **0.025641026** | **NCBP1** |
| **GO:0045924** | **regulation of female receptivity** | **1** | **54.37762238** | | **0.018240841** | **1.738955139** | **0.025641026** | **NCOA2** |
| **GO:2000322** | **regulation of glucocorticoid receptor signaling pathway** | **1** | **54.37762238** | | **0.018240841** | **1.738955139** | **0.025641026** | **NCOA2** |
| **GO:0043500** | **muscle adaptation** | **2** | **9.64764268** | | **0.01826821** | **1.738303998** | **0.051282051** | **CAMK2D//PTK2** |
| **GO:0051963** | **regulation of synapse assembly** | **2** | **9.64764268** | | **0.01826821** | **1.738303998** | **0.051282051** | **PTK2//STAU2** |
| **GO:0051272** | **positive regulation of cellular component movement** | **4** | **3.935222672** | | **0.018269619** | **1.73827052** | **0.102564103** | **PTK2//TSC2//CAMK2D//CFAP69** |
| **GO:1902414** | **protein localization to cell junction** | **2** | **9.570461538** | | **0.018545869** | **1.73175282** | **0.051282051** | **STAU2//MAP1A** |
| **GO:0060996** | **dendritic spine development** | **2** | **9.419745609** | | **0.019106558** | **1.718817552** | **0.051282051** | **TSC2//STAU2** |
| **GO:0044057** | **regulation of system process** | **4** | **3.877820721** | | **0.019172675** | **1.717317285** | **0.102564103** | **CACNA1E//CAMK2D//PTK2//RAB11FIP3** |
| **GO:0043244** | **regulation of protein-containing complex disassembly** | **2** | **9.346153846** | | **0.019389575** | **1.712431704** | **0.051282051** | **MAP1A//SVIL** |
| **GO:0014065** | **phosphatidylinositol 3-kinase signaling** | **2** | **9.273703041** | | **0.019674366** | **1.706099243** | **0.051282051** | **TSC2//PTK2** |
| **GO:0060294** | **cilium movement involved in cell motility** | **2** | **9.273703041** | | **0.019674366** | **1.706099243** | **0.051282051** | **CFAP69//CACNA1E** |
| **GO:0051128** | **regulation of cellular component organization** | **9** | **2.156804734** | | **0.019696588** | **1.705609006** | **0.230769231** | **MAP1A//CDC27//TSC2//PTK2//FXN//NCBP1//STAU2//RAP2A//SVIL** |
| **GO:0042789** | **mRNA transcription by RNA polymerase II** | **1** | **49.84615385** | | **0.019882931** | **1.701519596** | **0.025641026** | **TFCP2** |
| **GO:0045842** | **positive regulation of mitotic metaphase/anaphase transition** | **1** | **49.84615385** | | **0.019882931** | **1.701519596** | **0.025641026** | **CDC27** |
| **GO:0098789** | **pre-mRNA cleavage required for polyadenylation** | **1** | **49.84615385** | | **0.019882931** | **1.701519596** | **0.025641026** | **NCBP1** |
| **GO:0098935** | **dendritic transport** | **1** | **49.84615385** | | **0.019882931** | **1.701519596** | **0.025641026** | **STAU2** |
| **GO:1901970** | **positive regulation of mitotic sister chromatid separation** | **1** | **49.84615385** | | **0.019882931** | **1.701519596** | **0.025641026** | **CDC27** |
| **GO:1902093** | **positive regulation of flagellated sperm motility** | **1** | **49.84615385** | | **0.019882931** | **1.701519596** | **0.025641026** | **CFAP69** |
| **GO:1902514** | **regulation of calcium ion transmembrane transport via high voltage-gated calcium channel** | **1** | **49.84615385** | | **0.019882931** | **1.701519596** | **0.025641026** | **CAMK2D** |
| **GO:1905214** | **regulation of RNA binding** | **1** | **49.84615385** | | **0.019882931** | **1.701519596** | **0.025641026** | **NCBP1** |
| **GO:2000155** | **positive regulation of cilium-dependent cell motility** | **1** | **49.84615385** | | **0.019882931** | **1.701519596** | **0.025641026** | **CFAP69** |
| **GO:0048640** | **negative regulation of developmental growth** | **2** | **9.132119789** | | **0.020249244** | **1.693591186** | **0.051282051** | **FXN//PTK2** |
| **GO:0034622** | **cellular protein-containing complex assembly** | **5** | **3.144867751** | | **0.020347705** | **1.69148456** | **0.128205128** | **NCBP1//DHX29//PTK2//SVIL//AHCTF1** |
| **GO:0099175** | **regulation of postsynapse organization** | **2** | **9.062937063** | | **0.020539318** | **1.687413989** | **0.051282051** | **STAU2//TSC2** |
| **GO:0001841** | **neural tube formation** | **2** | **8.994794679** | | **0.020831139** | **1.681286977** | **0.051282051** | **TEAD2//TSC2** |
| **GO:0001731** | **formation of translation preinitiation complex** | **1** | **46.01183432** | | **0.021522344** | **1.667110422** | **0.025641026** | **DHX29** |
| **GO:0003353** | **positive regulation of cilium movement** | **1** | **46.01183432** | | **0.021522344** | **1.667110422** | **0.025641026** | **CFAP69** |
| **GO:0032486** | **Rap protein signal transduction** | **1** | **46.01183432** | | **0.021522344** | **1.667110422** | **0.025641026** | **RAP2A** |
| **GO:0048548** | **regulation of pinocytosis** | **1** | **46.01183432** | | **0.021522344** | **1.667110422** | **0.025641026** | **TSC2** |
| **GO:0055119** | **relaxation of cardiac muscle** | **1** | **46.01183432** | | **0.021522344** | **1.667110422** | **0.025641026** | **CAMK2D** |
| **GO:0061577** | **calcium ion transmembrane transport via high voltage-gated calcium channel** | **1** | **46.01183432** | | **0.021522344** | **1.667110422** | **0.025641026** | **CAMK2D** |
| **GO:0090085** | **regulation of protein deubiquitination** | **1** | **46.01183432** | | **0.021522344** | **1.667110422** | **0.025641026** | **TRIM21** |
| **GO:0098787** | **mRNA cleavage involved in mRNA processing** | **1** | **46.01183432** | | **0.021522344** | **1.667110422** | **0.025641026** | **NCBP1** |
| **GO:0099532** | **synaptic vesicle endosomal processing** | **1** | **46.01183432** | | **0.021522344** | **1.667110422** | **0.025641026** | **VTI1A** |
| **GO:1902101** | **positive regulation of metaphase/anaphase transition of cell cycle** | **1** | **46.01183432** | | **0.021522344** | **1.667110422** | **0.025641026** | **CDC27** |
| **GO:2000650** | **negative regulation of sodium ion transmembrane transporter activity** | **1** | **46.01183432** | | **0.021522344** | **1.667110422** | **0.025641026** | **CAMK2D** |
| **GO:0001539** | **cilium or flagellum-dependent cell motility** | **2** | **8.79638009** | | **0.021717029** | **1.663199579** | **0.051282051** | **CFAP69//CACNA1E** |
| **GO:0060285** | **cilium-dependent cell motility** | **2** | **8.79638009** | | **0.021717029** | **1.663199579** | **0.051282051** | **CFAP69//CACNA1E** |
| **GO:0045787** | **positive regulation of cell cycle** | **3** | **5.012462398** | | **0.02178863** | **1.661770068** | **0.076923077** | **CAMK2D//CDC27//TRIM21** |
| **GO:0044271** | **cellular nitrogen compound biosynthetic process** | **13** | **1.776153495** | | **0.021843251** | **1.660682728** | **0.333333333** | **NCOA2//DHX29//GRHL1//TEAD2//ZFP426//GMCL1//TFCP2//GTF3C2//NCBP1//FXN//TRIM21//TSC2//CAMK2D** |
| **GO:0050767** | **regulation of neurogenesis** | **5** | **3.061176285** | | **0.022570735** | **1.646454296** | **0.128205128** | **TSC2//FXN//RAP2A//PTK2//STAU2** |
| **GO:1990778** | **protein localization to cell periphery** | **3** | **4.943420216** | | **0.022588099** | **1.646120311** | **0.076923077** | **TSC2//RAP2A//CAMK2D** |
| **GO:0048699** | **generation of neurons** | **7** | **2.428698911** | | **0.022646281** | **1.645003112** | **0.179487179** | **PTK2//FKTN//MAP1A//TSC2//FXN//RAP2A//STAU2** |
| **GO:0006999** | **nuclear pore organization** | **1** | **42.72527473** | | **0.023159086** | **1.635278585** | **0.025641026** | **AHCTF1** |
| **GO:0010644** | **cell communication by electrical coupling** | **1** | **42.72527473** | | **0.023159086** | **1.635278585** | **0.025641026** | **CAMK2D** |
| **GO:0042048** | **olfactory behavior** | **1** | **42.72527473** | | **0.023159086** | **1.635278585** | **0.025641026** | **CFAP69** |
| **GO:0042921** | **glucocorticoid receptor signaling pathway** | **1** | **42.72527473** | | **0.023159086** | **1.635278585** | **0.025641026** | **NCOA2** |
| **GO:0045792** | **negative regulation of cell size** | **1** | **42.72527473** | | **0.023159086** | **1.635278585** | **0.025641026** | **TSC2** |
| **GO:0060180** | **female mating behavior** | **1** | **42.72527473** | | **0.023159086** | **1.635278585** | **0.025641026** | **NCOA2** |
| **GO:0090110** | **COPII-coated vesicle cargo loading** | **1** | **42.72527473** | | **0.023159086** | **1.635278585** | **0.025641026** | **SEC23B** |
| **GO:0090161** | **Golgi ribbon formation** | **1** | **42.72527473** | | **0.023159086** | **1.635278585** | **0.025641026** | **VTI1A** |
| **GO:1902306** | **negative regulation of sodium ion transmembrane transport** | **1** | **42.72527473** | | **0.023159086** | **1.635278585** | **0.025641026** | **CAMK2D** |
| **GO:1905820** | **positive regulation of chromosome separation** | **1** | **42.72527473** | | **0.023159086** | **1.635278585** | **0.025641026** | **CDC27** |
| **GO:2000145** | **regulation of cell motility** | **5** | **3.033234514** | | **0.023375995** | **1.63122989** | **0.128205128** | **PTK2//TSC2//CAMK2D//RAP2A//CFAP69** |
| **GO:0032502** | **developmental process** | **17** | **1.576773978** | | **0.023516968** | **1.628618663** | **0.435897436** | **PTK2//TEAD2//FKTN//TSC2//GRHL1//AHCTF1//GMCL1//CFAP69//MAP1A//SVIL//FMNL2//FXN//TMEM120B//IQCB1//STAU2//RAP2A//CAMK2D** |
| **GO:0010976** | **positive regulation of neuron projection development** | **3** | **4.82382134** | | **0.024067394** | **1.618570924** | **0.076923077** | **FXN//TSC2//STAU2** |
| **GO:0010508** | **positive regulation of autophagy** | **2** | **8.307692308** | | **0.02415475** | **1.61699746** | **0.051282051** | **TSC2//TRIM21** |
| **GO:0043624** | **cellular protein complex disassembly** | **2** | **8.307692308** | | **0.02415475** | **1.61699746** | **0.051282051** | **MAP1A//SVIL** |
| **GO:0032880** | **regulation of protein localization** | **5** | **3.005798222** | | **0.024199891** | **1.61618659** | **0.128205128** | **CACNA1E//RAB11FIP3//MAP1A//CAMK2D//TRIM21** |
| **GO:0051962** | **positive regulation of nervous system development** | **4** | **3.587129512** | | **0.024696189** | **1.607370067** | **0.102564103** | **TSC2//FXN//STAU2//PTK2** |
| **GO:0010960** | **magnesium ion homeostasis** | **1** | **39.87692308** | | **0.02479316** | **1.605668122** | **0.025641026** | **CNNM2** |
| **GO:0015693** | **magnesium ion transport** | **1** | **39.87692308** | | **0.02479316** | **1.605668122** | **0.025641026** | **CNNM2** |
| **GO:0030007** | **cellular potassium ion homeostasis** | **1** | **39.87692308** | | **0.02479316** | **1.605668122** | **0.025641026** | **CAMK2D** |
| **GO:0031958** | **corticosteroid receptor signaling pathway** | **1** | **39.87692308** | | **0.02479316** | **1.605668122** | **0.025641026** | **NCOA2** |
| **GO:0060049** | **regulation of protein glycosylation** | **1** | **39.87692308** | | **0.02479316** | **1.605668122** | **0.025641026** | **FKTN** |
| **GO:0060396** | **growth hormone receptor signaling pathway** | **1** | **39.87692308** | | **0.02479316** | **1.605668122** | **0.025641026** | **PTK2** |
| **GO:0071378** | **cellular response to growth hormone stimulus** | **1** | **39.87692308** | | **0.02479316** | **1.605668122** | **0.025641026** | **PTK2** |
| **GO:2000811** | **negative regulation of anoikis** | **1** | **39.87692308** | | **0.02479316** | **1.605668122** | **0.025641026** | **PTK2** |
| **GO:0034645** | **cellular macromolecule biosynthetic process** | **13** | **1.745454545** | | **0.024989419** | **1.602243841** | **0.333333333** | **NCOA2//DHX29//GRHL1//TEAD2//ZFP426//GMCL1//TFCP2//GTF3C2//NCBP1//FKTN//TRIM21//TSC2//CAMK2D** |
| **GO:0048523** | **negative regulation of cellular process** | **14** | **1.691748252** | | **0.025046151** | **1.601259009** | **0.358974359** | **NCOA2//TSC2//MAP1A//FKTN//PTK2//CAMK2D//RAP2A//FXN//IQCB1//SVIL//TEAD2//RAB11FIP3//TRIM21//STAU2** |
| **GO:0048856** | **anatomical structure development** | **16** | **1.601214914** | | **0.025386738** | **1.595393092** | **0.41025641** | **PTK2//TEAD2//FKTN//TSC2//GRHL1//AHCTF1//GMCL1//CFAP69//MAP1A//SVIL//FMNL2//FXN//IQCB1//STAU2//RAP2A//CAMK2D** |
| **GO:0010720** | **positive regulation of cell development** | **4** | **3.539371871** | | **0.025783244** | **1.588662444** | **0.102564103** | **TSC2//FXN//PTK2//STAU2** |
| **GO:0044260** | **cellular macromolecule metabolic process** | **19** | **1.497552125** | | **0.025832277** | **1.587837307** | **0.487179487** | **NCOA2//PTK2//NCBP1//TRIM21//CWC27//DHX29//TSC2//RAP2A//GRHL1//TEAD2//ZFP426//GMCL1//TFCP2//GTF3C2//CAMK2D//FKTN//CDC27//FXN//MAP1A** |
| **GO:0014067** | **negative regulation of phosphatidylinositol 3-kinase signaling** | **1** | **37.38461538** | | **0.02642457** | **1.577992072** | **0.025641026** | **TSC2** |
| **GO:0035641** | **locomotory exploration behavior** | **1** | **37.38461538** | | **0.02642457** | **1.577992072** | **0.025641026** | **LSAMP** |
| **GO:1901077** | **regulation of relaxation of muscle** | **1** | **37.38461538** | | **0.02642457** | **1.577992072** | **0.025641026** | **CAMK2D** |
| **GO:1902187** | **negative regulation of viral release from host cell** | **1** | **37.38461538** | | **0.02642457** | **1.577992072** | **0.025641026** | **TRIM21** |
| **GO:1904262** | **negative regulation of TORC1 signaling** | **1** | **37.38461538** | | **0.02642457** | **1.577992072** | **0.025641026** | **TSC2** |
| **GO:1905516** | **positive regulation of fertilization** | **1** | **37.38461538** | | **0.02642457** | **1.577992072** | **0.025641026** | **CFAP69** |
| **GO:0048519** | **negative regulation of biological process** | **15** | **1.631328671** | | **0.02690778** | **1.570122137** | **0.384615385** | **NCOA2//NCBP1//TSC2//MAP1A//FKTN//PTK2//CAMK2D//RAP2A//TRIM21//FXN//IQCB1//SVIL//TEAD2//RAB11FIP3//STAU2** |
| **GO:0008360** | **regulation of cell shape** | **2** | **7.768231768** | | **0.027352115** | **1.56300909** | **0.051282051** | **PTK2//FMNL2** |
| **GO:0009059** | **macromolecule biosynthetic process** | **13** | **1.723404255** | | **0.027533765** | **1.560134405** | **0.333333333** | **NCOA2//DHX29//GRHL1//TEAD2//ZFP426//GMCL1//TFCP2//GTF3C2//NCBP1//FKTN//TRIM21//TSC2//CAMK2D** |
| **GO:0001838** | **embryonic epithelial tube formation** | **2** | **7.718114144** | | **0.027680818** | **1.557821077** | **0.051282051** | **TEAD2//TSC2** |
| **GO:0040012** | **regulation of locomotion** | **5** | **2.895226748** | | **0.027887299** | **1.554593545** | **0.128205128** | **PTK2//TSC2//CAMK2D//RAP2A//CFAP69** |
| **GO:0031145** | **anaphase-promoting complex-dependent catabolic process** | **1** | **35.18552036** | | **0.028053321** | **1.55201572** | **0.025641026** | **CDC27** |
| **GO:0032570** | **response to progesterone** | **1** | **35.18552036** | | **0.028053321** | **1.55201572** | **0.025641026** | **NCOA2** |
| **GO:0035269** | **protein O-linked mannosylation** | **1** | **35.18552036** | | **0.028053321** | **1.55201572** | **0.025641026** | **FKTN** |
| **GO:0035459** | **vesicle cargo loading** | **1** | **35.18552036** | | **0.028053321** | **1.55201572** | **0.025641026** | **SEC23B** |
| **GO:0040015** | **negative regulation of multicellular organism growth** | **1** | **35.18552036** | | **0.028053321** | **1.55201572** | **0.025641026** | **FXN** |
| **GO:0048368** | **lateral mesoderm development** | **1** | **35.18552036** | | **0.028053321** | **1.55201572** | **0.025641026** | **TEAD2** |
| **GO:0051014** | **actin filament severing** | **1** | **35.18552036** | | **0.028053321** | **1.55201572** | **0.025641026** | **SVIL** |
| **GO:1900452** | **regulation of long-term synaptic depression** | **1** | **35.18552036** | | **0.028053321** | **1.55201572** | **0.025641026** | **STAU2** |
| **GO:0031331** | **positive regulation of cellular catabolic process** | **3** | **4.531468531** | | **0.028265186** | **1.548748153** | **0.076923077** | **TRIM21//TSC2//PTK2** |
| **GO:2000058** | **regulation of ubiquitin-dependent protein catabolic process** | **2** | **7.52394775** | | **0.029011634** | **1.537427811** | **0.051282051** | **MAP1A//PTK2** |
| **GO:0000910** | **cytokinesis** | **2** | **7.476923077** | | **0.029348309** | **1.532416923** | **0.051282051** | **RAB11FIP3//AHCTF1** |
| **GO:0051302** | **regulation of cell division** | **2** | **7.476923077** | | **0.029348309** | **1.532416923** | **0.051282051** | **RAB11FIP3//AHCTF1** |
| **GO:0030036** | **actin cytoskeleton organization** | **4** | **3.393780687** | | **0.029482361** | **1.530437748** | **0.102564103** | **SVIL//FMNL2//RAP2A//STAU2** |
| **GO:0006896** | **Golgi to vacuole transport** | **1** | **33.23076923** | | **0.029679417** | **1.527544638** | **0.025641026** | **VTI1A** |
| **GO:0009299** | **mRNA transcription** | **1** | **33.23076923** | | **0.029679417** | **1.527544638** | **0.025641026** | **TFCP2** |
| **GO:0016540** | **protein autoprocessing** | **1** | **33.23076923** | | **0.029679417** | **1.527544638** | **0.025641026** | **FXN** |
| **GO:0030033** | **microvillus assembly** | **1** | **33.23076923** | | **0.029679417** | **1.527544638** | **0.025641026** | **RAP2A** |
| **GO:0060416** | **response to growth hormone** | **1** | **33.23076923** | | **0.029679417** | **1.527544638** | **0.025641026** | **PTK2** |
| **GO:1901317** | **regulation of flagellated sperm motility** | **1** | **33.23076923** | | **0.029679417** | **1.527544638** | **0.025641026** | **CFAP69** |
| **GO:0072175** | **epithelial tube formation** | **2** | **7.384615385** | | **0.030026381** | **1.522497008** | **0.051282051** | **TEAD2//TSC2** |
| **GO:0045935** | **positive regulation of nucleobase-containing compound metabolic process** | **7** | **2.288020177** | | **0.03020853** | **1.519870405** | **0.179487179** | **NCBP1//TFCP2//TEAD2//NCOA2//GRHL1//TSC2//CAMK2D** |
| **GO:1902904** | **negative regulation of supramolecular fiber organization** | **2** | **7.339310996** | | **0.030367767** | **1.517587139** | **0.051282051** | **MAP1A//SVIL** |
| **GO:0022008** | **neurogenesis** | **7** | **2.281785789** | | **0.030603348** | **1.514231059** | **0.179487179** | **PTK2//FKTN//MAP1A//TSC2//FXN//RAP2A//STAU2** |
| **GO:0010970** | **transport along microtubule** | **2** | **7.294559099** | | **0.030710712** | **1.512710116** | **0.051282051** | **STAU2//MAP1A** |
| **GO:0048015** | **phosphatidylinositol-mediated signaling** | **2** | **7.294559099** | | **0.030710712** | **1.512710116** | **0.051282051** | **TSC2//PTK2** |
| **GO:0006606** | **protein import into nucleus** | **2** | **7.25034965** | | **0.031055209** | **1.507865538** | **0.051282051** | **IPO11//TSC2** |
| **GO:0048660** | **regulation of smooth muscle cell proliferation** | **2** | **7.25034965** | | **0.031055209** | **1.507865538** | **0.051282051** | **PTK2//CAMK2D** |
| **GO:0043408** | **regulation of MAPK cascade** | **4** | **3.336981011** | | **0.031101481** | **1.507218926** | **0.102564103** | **TSC2//RAP2A//FKTN//CAMK2D** |
| **GO:0010766** | **negative regulation of sodium ion transport** | **1** | **31.48178138** | | **0.031302862** | **1.504415957** | **0.025641026** | **CAMK2D** |
| **GO:0010880** | **regulation of release of sequestered calcium ion into cytosol by sarcoplasmic reticulum** | **1** | **31.48178138** | | **0.031302862** | **1.504415957** | **0.025641026** | **CAMK2D** |
| **GO:0033145** | **positive regulation of intracellular steroid hormone receptor signaling pathway** | **1** | **31.48178138** | | **0.031302862** | **1.504415957** | **0.025641026** | **NCOA2** |
| **GO:0035020** | **regulation of Rac protein signal transduction** | **1** | **31.48178138** | | **0.031302862** | **1.504415957** | **0.025641026** | **CAMK2D** |
| **GO:0035372** | **protein localization to microtubule** | **1** | **31.48178138** | | **0.031302862** | **1.504415957** | **0.025641026** | **MAP1A** |
| **GO:0050884** | **neuromuscular process controlling posture** | **1** | **31.48178138** | | **0.031302862** | **1.504415957** | **0.025641026** | **FXN** |
| **GO:0051349** | **positive regulation of lyase activity** | **1** | **31.48178138** | | **0.031302862** | **1.504415957** | **0.025641026** | **FXN** |
| **GO:0090201** | **negative regulation of release of cytochrome c from mitochondria** | **1** | **31.48178138** | | **0.031302862** | **1.504415957** | **0.025641026** | **FXN** |
| **GO:0051896** | **regulation of protein kinase B signaling** | **2** | **7.206672845** | | **0.031401254** | **1.503053009** | **0.051282051** | **PTK2//TSC2** |
| **GO:0032535** | **regulation of cellular component size** | **3** | **4.344943192** | | **0.031460848** | **1.502229571** | **0.076923077** | **FXN//TSC2//SVIL** |
| **GO:0009987** | **cellular process** | **34** | **1.188338832** | | **0.031717033** | **1.498707447** | **0.871794872** | **CAMK2D//NCOA2//PTK2//NCBP1//TRIM21//MAP1A//U2AF1L4//CWC27//TEAD2//DHX29//FKTN//TSC2//RAP2A//GRHL1//FXN//ZFP426//GMCL1//TFCP2//GTF3C2//IPO11//VTI1A//SEC23B//SVIL//FMNL2//RAB11FIP3//AHCTF1//CDC27//LSAMP//CACNA1E//CFAP69//IQCB1//STAU2//TMEM120B//PDIA5** |
| **GO:0048017** | **inositol lipid-mediated signaling** | **2** | **7.163519116** | | **0.03174884** | **1.498272144** | **0.051282051** | **TSC2//PTK2** |
| **GO:0051170** | **import into nucleus** | **2** | **7.078743741** | | **0.032448611** | **1.488803893** | **0.051282051** | **TSC2//IPO11** |
| **GO:0051270** | **regulation of cellular component movement** | **5** | **2.776944504** | | **0.032588476** | **1.486935946** | **0.128205128** | **PTK2//TSC2//CAMK2D//RAP2A//CFAP69** |
| **GO:0048659** | **smooth muscle cell proliferation** | **2** | **7.037104072** | | **0.032800785** | **1.484115767** | **0.051282051** | **PTK2//CAMK2D** |
| **GO:0010639** | **negative regulation of organelle organization** | **3** | **4.272527473** | | **0.032830076** | **1.483728111** | **0.076923077** | **MAP1A//SVIL//FXN** |
| **GO:0006379** | **mRNA cleavage** | **1** | **29.90769231** | | **0.03292366** | **1.482491891** | **0.025641026** | **NCBP1** |
| **GO:0030002** | **cellular anion homeostasis** | **1** | **29.90769231** | | **0.03292366** | **1.482491891** | **0.025641026** | **PTK2** |
| **GO:0030320** | **cellular monovalent inorganic anion homeostasis** | **1** | **29.90769231** | | **0.03292366** | **1.482491891** | **0.025641026** | **PTK2** |
| **GO:0060252** | **positive regulation of glial cell proliferation** | **1** | **29.90769231** | | **0.03292366** | **1.482491891** | **0.025641026** | **PTK2** |
| **GO:0045931** | **positive regulation of mitotic cell cycle** | **2** | **6.995951417** | | **0.033154477** | **1.479457825** | **0.051282051** | **CAMK2D//CDC27** |
| **GO:0051494** | **negative regulation of cytoskeleton organization** | **2** | **6.995951417** | | **0.033154477** | **1.479457825** | **0.051282051** | **MAP1A//SVIL** |
| **GO:0022618** | **ribonucleoprotein complex assembly** | **2** | **6.955277281** | | **0.03350968** | **1.474829714** | **0.051282051** | **NCBP1//DHX29** |
| **GO:2000112** | **regulation of cellular macromolecule biosynthetic process** | **11** | **1.79723909** | | **0.033601847** | **1.473636854** | **0.282051282** | **NCOA2//GRHL1//TEAD2//ZFP426//GMCL1//TFCP2//NCBP1//TRIM21//TSC2//FKTN//CAMK2D** |
| **GO:0043409** | **negative regulation of MAPK cascade** | **2** | **6.915073366** | | **0.033866391** | **1.470231085** | **0.051282051** | **TSC2//FKTN** |
| **GO:0072594** | **establishment of protein localization to organelle** | **3** | **4.212351029** | | **0.034028395** | **1.468158538** | **0.076923077** | **TSC2//IPO11//VTI1A** |
| **GO:0031329** | **regulation of cellular catabolic process** | **4** | **3.233264033** | | **0.034350876** | **1.464062181** | **0.102564103** | **PTK2//TRIM21//TSC2//MAP1A** |
| **GO:0034330** | **cell junction organization** | **4** | **3.233264033** | | **0.034350876** | **1.464062181** | **0.102564103** | **GRHL1//PTK2//STAU2//TSC2** |
| **GO:0007288** | **sperm axoneme assembly** | **1** | **28.48351648** | | **0.034541816** | **1.461654834** | **0.025641026** | **CFAP69** |
| **GO:0010039** | **response to iron ion** | **1** | **28.48351648** | | **0.034541816** | **1.461654834** | **0.025641026** | **FXN** |
| **GO:0014808** | **release of sequestered calcium ion into cytosol by sarcoplasmic reticulum** | **1** | **28.48351648** | | **0.034541816** | **1.461654834** | **0.025641026** | **CAMK2D** |
| **GO:0045475** | **locomotor rhythm** | **1** | **28.48351648** | | **0.034541816** | **1.461654834** | **0.025641026** | **NCOA2** |
| **GO:0055064** | **chloride ion homeostasis** | **1** | **28.48351648** | | **0.034541816** | **1.461654834** | **0.025641026** | **PTK2** |
| **GO:0062033** | **positive regulation of mitotic sister chromatid segregation** | **1** | **28.48351648** | | **0.034541816** | **1.461654834** | **0.025641026** | **CDC27** |
| **GO:2000209** | **regulation of anoikis** | **1** | **28.48351648** | | **0.034541816** | **1.461654834** | **0.025641026** | **PTK2** |
| **GO:0007612** | **learning** | **2** | **6.836043956** | | **0.034584307** | **1.461120919** | **0.051282051** | **MAP1A//CACNA1E** |
| **GO:0010977** | **negative regulation of neuron projection development** | **2** | **6.797202797** | | **0.034945502** | **1.456608715** | **0.051282051** | **PTK2//TSC2** |
| **GO:0035148** | **tube formation** | **2** | **6.758800522** | | **0.03530818** | **1.452124663** | **0.051282051** | **TEAD2//TSC2** |
| **GO:0051960** | **regulation of nervous system development** | **5** | **2.711486157** | | **0.035589381** | **1.44867956** | **0.128205128** | **TSC2//FXN//RAP2A//PTK2//STAU2** |
| **GO:0071826** | **ribonucleoprotein complex subunit organization** | **2** | **6.683283197** | | **0.036037964** | **1.443239747** | **0.051282051** | **NCBP1//DHX29** |
| **GO:0000413** | **protein peptidyl-prolyl isomerization** | **1** | **27.18881119** | | **0.036157334** | **1.441803604** | **0.025641026** | **CWC27** |
| **GO:0006783** | **heme biosynthetic process** | **1** | **27.18881119** | | **0.036157334** | **1.441803604** | **0.025641026** | **FXN** |
| **GO:0007635** | **chemosensory behavior** | **1** | **27.18881119** | | **0.036157334** | **1.441803604** | **0.025641026** | **CFAP69** |
| **GO:0010666** | **positive regulation of cardiac muscle cell apoptotic process** | **1** | **27.18881119** | | **0.036157334** | **1.441803604** | **0.025641026** | **CAMK2D** |
| **GO:0016226** | **iron-sulfur cluster assembly** | **1** | **27.18881119** | | **0.036157334** | **1.441803604** | **0.025641026** | **FXN** |
| **GO:0031163** | **metallo-sulfur cluster assembly** | **1** | **27.18881119** | | **0.036157334** | **1.441803604** | **0.025641026** | **FXN** |
| **GO:0035268** | **protein mannosylation** | **1** | **27.18881119** | | **0.036157334** | **1.441803604** | **0.025641026** | **FKTN** |
| **GO:0048339** | **paraxial mesoderm development** | **1** | **27.18881119** | | **0.036157334** | **1.441803604** | **0.025641026** | **TEAD2** |
| **GO:1903514** | **release of sequestered calcium ion into cytosol by endoplasmic reticulum** | **1** | **27.18881119** | | **0.036157334** | **1.441803604** | **0.025641026** | **CAMK2D** |
| **GO:2000010** | **positive regulation of protein localization to cell surface** | **1** | **27.18881119** | | **0.036157334** | **1.441803604** | **0.025641026** | **MAP1A** |
| **GO:0007264** | **small GTPase mediated signal transduction** | **3** | **4.10631931** | | **0.036284323** | **1.440280976** | **0.076923077** | **RAP2A//CAMK2D//TSC2** |
| **GO:0003341** | **cilium movement** | **2** | **6.646153846** | | **0.036405059** | **1.438838261** | **0.051282051** | **CFAP69//CACNA1E** |
| **GO:0001558** | **regulation of cell growth** | **3** | **4.096944152** | | **0.036493184** | **1.437788245** | **0.076923077** | **PTK2//FXN//NCBP1** |
| **GO:0010556** | **regulation of macromolecule biosynthetic process** | **11** | **1.773024066** | | **0.036702513** | **1.435304204** | **0.282051282** | **NCOA2//GRHL1//TEAD2//ZFP426//GMCL1//TFCP2//NCBP1//TRIM21//TSC2//FKTN//CAMK2D** |
| **GO:0016477** | **cell migration** | **6** | **2.39102137** | | **0.036998692** | **1.431813624** | **0.153846154** | **PTK2//FKTN//TSC2//CAMK2D//RAP2A//FMNL2** |
| **GO:0048813** | **dendrite morphogenesis** | **2** | **6.573119189** | | **0.037143625** | **1.430115718** | **0.051282051** | **RAP2A//STAU2** |
| **GO:0016331** | **morphogenesis of embryonic epithelium** | **2** | **6.537200504** | | **0.037515085** | **1.42579407** | **0.051282051** | **TEAD2//TSC2** |
| **GO:0060284** | **regulation of cell development** | **5** | **2.667947574** | | **0.037765552** | **1.422904161** | **0.128205128** | **TSC2//FXN//RAP2A//PTK2//STAU2** |
| **GO:0006907** | **pinocytosis** | **1** | **26.00668896** | | **0.037770217** | **1.422850519** | **0.025641026** | **TSC2** |
| **GO:0010663** | **positive regulation of striated muscle cell apoptotic process** | **1** | **26.00668896** | | **0.037770217** | **1.422850519** | **0.025641026** | **CAMK2D** |
| **GO:0046931** | **pore complex assembly** | **1** | **26.00668896** | | **0.037770217** | **1.422850519** | **0.025641026** | **AHCTF1** |
| **GO:0051291** | **protein heterooligomerization** | **1** | **26.00668896** | | **0.037770217** | **1.422850519** | **0.025641026** | **TMEM120B** |
| **GO:0097502** | **mannosylation** | **1** | **26.00668896** | | **0.037770217** | **1.422850519** | **0.025641026** | **FKTN** |
| **GO:0101023** | **vascular endothelial cell proliferation** | **1** | **26.00668896** | | **0.037770217** | **1.422850519** | **0.025641026** | **TSC2** |
| **GO:1905562** | **regulation of vascular endothelial cell proliferation** | **1** | **26.00668896** | | **0.037770217** | **1.422850519** | **0.025641026** | **TSC2** |
| **GO:0018130** | **heterocycle biosynthetic process** | **11** | **1.760688335** | | **0.038394775** | **1.415727868** | **0.282051282** | **NCOA2//GRHL1//TEAD2//ZFP426//GMCL1//TFCP2//GTF3C2//FXN//TRIM21//TSC2//CAMK2D** |
| **GO:0098771** | **inorganic ion homeostasis** | **4** | **3.119446395** | | **0.03841204** | **1.415532629** | **0.102564103** | **FXN//CAMK2D//CNNM2//PTK2** |
| **GO:0046328** | **regulation of JNK cascade** | **2** | **6.431761787** | | **0.038638105** | **1.412984178** | **0.051282051** | **FKTN//RAP2A** |
| **GO:0050793** | **regulation of developmental process** | **9** | **1.913752085** | | **0.039189465** | **1.406830662** | **0.230769231** | **PTK2//FMNL2//TSC2//FXN//GRHL1//IQCB1//RAP2A//STAU2//TEAD2** |
| **GO:0019438** | **aromatic compound biosynthetic process** | **11** | **1.754584615** | | **0.039261802** | **1.406029776** | **0.282051282** | **NCOA2//GRHL1//TEAD2//ZFP426//GMCL1//TFCP2//GTF3C2//FXN//TRIM21//TSC2//CAMK2D** |
| **GO:0008090** | **retrograde axonal transport** | **1** | **24.92307692** | | **0.039380471** | **1.404719096** | **0.025641026** | **MAP1A** |
| **GO:0030903** | **notochord development** | **1** | **24.92307692** | | **0.039380471** | **1.404719096** | **0.025641026** | **TEAD2** |
| **GO:0031954** | **positive regulation of protein autophosphorylation** | **1** | **24.92307692** | | **0.039380471** | **1.404719096** | **0.025641026** | **RAP2A** |
| **GO:0010770** | **positive regulation of cell morphogenesis involved in differentiation** | **2** | **6.363338789** | | **0.039393931** | **1.404570681** | **0.051282051** | **FXN//STAU2** |
| **GO:0043170** | **macromolecule metabolic process** | **21** | **1.389209331** | | **0.03976493** | **1.400499776** | **0.538461538** | **NCOA2//PTK2//NCBP1//TRIM21//U2AF1L4//CWC27//DHX29//TSC2//RAP2A//GRHL1//TEAD2//ZFP426//GMCL1//TFCP2//GTF3C2//CAMK2D//FKTN//STAU2//FXN//CDC27//MAP1A** |
| **GO:0001764** | **neuron migration** | **2** | **6.32967033** | | **0.039773971** | **1.400401051** | **0.051282051** | **PTK2//FKTN** |
| **GO:0007416** | **synapse assembly** | **2** | **6.263391059** | | **0.040538276** | **1.392134722** | **0.051282051** | **PTK2//STAU2** |
| **GO:0002183** | **cytoplasmic translational initiation** | **1** | **23.92615385** | | **0.040988099** | **1.387342226** | **0.025641026** | **DHX29** |
| **GO:0010759** | **positive regulation of macrophage chemotaxis** | **1** | **23.92615385** | | **0.040988099** | **1.387342226** | **0.025641026** | **PTK2** |
| **GO:0032897** | **negative regulation of viral transcription** | **1** | **23.92615385** | | **0.040988099** | **1.387342226** | **0.025641026** | **TRIM21** |
| **GO:0048266** | **behavioral response to pain** | **1** | **23.92615385** | | **0.040988099** | **1.387342226** | **0.025641026** | **CACNA1E** |
| **GO:0060295** | **regulation of cilium movement involved in cell motility** | **1** | **23.92615385** | | **0.040988099** | **1.387342226** | **0.025641026** | **CFAP69** |
| **GO:0090114** | **COPII-coated vesicle budding** | **1** | **23.92615385** | | **0.040988099** | **1.387342226** | **0.025641026** | **SEC23B** |
| **GO:1902019** | **regulation of cilium-dependent cell motility** | **1** | **23.92615385** | | **0.040988099** | **1.387342226** | **0.025641026** | **CFAP69** |
| **GO:0030182** | **neuron differentiation** | **6** | **2.328957221** | | **0.041267179** | **1.384395215** | **0.153846154** | **MAP1A//TSC2//PTK2//FXN//RAP2A//STAU2** |
| **GO:0031325** | **positive regulation of cellular metabolic process** | **10** | **1.809845223** | | **0.041409606** | **1.3828989** | **0.256410256** | **PTK2//RAP2A//TRIM21//TSC2//NCBP1//TFCP2//TEAD2//NCOA2//GRHL1//CAMK2D** |
| **GO:0009893** | **positive regulation of metabolic process** | **11** | **1.739738844** | | **0.041456227** | **1.382410227** | **0.282051282** | **PTK2//RAP2A//TRIM21//TSC2//NCBP1//TFCP2//TEAD2//NCOA2//GRHL1//CAMK2D//FXN** |
| **GO:0021915** | **neural tube development** | **2** | **6.166534496** | | **0.041695218** | **1.379913754** | **0.051282051** | **TEAD2//TSC2** |
| **GO:0030705** | **cytoskeleton-dependent intracellular transport** | **2** | **6.166534496** | | **0.041695218** | **1.379913754** | **0.051282051** | **STAU2//MAP1A** |
| **GO:0009896** | **positive regulation of catabolic process** | **3** | **3.875726865** | | **0.041917938** | **1.37760009** | **0.076923077** | **TRIM21//TSC2//PTK2** |
| **GO:0099111** | **microtubule-based transport** | **2** | **6.134911243** | | **0.042083638** | **1.37588672** | **0.051282051** | **STAU2//MAP1A** |
| **GO:0043491** | **protein kinase B signaling** | **2** | **6.103610675** | | **0.042473437** | **1.371882596** | **0.051282051** | **PTK2//TSC2** |
| **GO:0031440** | **regulation of mRNA 3'-end processing** | **1** | **23.00591716** | | **0.042593105** | **1.370660698** | **0.025641026** | **NCBP1** |
| **GO:0098901** | **regulation of cardiac muscle cell action potential** | **1** | **23.00591716** | | **0.042593105** | **1.370660698** | **0.025641026** | **CAMK2D** |
| **GO:0070838** | **divalent metal ion transport** | **3** | **3.842530061** | | **0.042821952** | **1.368333543** | **0.076923077** | **CAMK2D//CACNA1E//CNNM2** |
| **GO:0072511** | **divalent inorganic cation transport** | **3** | **3.818003273** | | **0.043506444** | **1.361446417** | **0.076923077** | **CAMK2D//CACNA1E//CNNM2** |
| **GO:0048522** | **positive regulation of cellular process** | **15** | **1.537934126** | | **0.043849925** | **1.358031144** | **0.384615385** | **PTK2//RAP2A//FXN//TRIM21//TSC2//CAMK2D//NCBP1//CDC27//TFCP2//TEAD2//NCOA2//GRHL1//STAU2//CFAP69//MAP1A** |
| **GO:0045666** | **positive regulation of neuron differentiation** | **3** | **3.801825293** | | **0.043965851** | **1.356884512** | **0.076923077** | **TSC2//FXN//STAU2** |
| **GO:0099173** | **postsynapse organization** | **2** | **5.981538462** | | **0.044046299** | **1.356090575** | **0.051282051** | **STAU2//TSC2** |
| **GO:0032879** | **regulation of localization** | **9** | **1.87313313** | | **0.044104373** | **1.355518348** | **0.230769231** | **CAMK2D//PTK2//TSC2//RAP2A//TRIM21//CACNA1E//RAB11FIP3//CFAP69//MAP1A** |
| **GO:0006779** | **porphyrin-containing compound biosynthetic process** | **1** | **22.15384615** | | **0.044195494** | **1.354622006** | **0.025641026** | **FXN** |
| **GO:0006891** | **intra-Golgi vesicle-mediated transport** | **1** | **22.15384615** | | **0.044195494** | **1.354622006** | **0.025641026** | **VTI1A** |
| **GO:0010971** | **positive regulation of G2/M transition of mitotic cell cycle** | **1** | **22.15384615** | | **0.044195494** | **1.354622006** | **0.025641026** | **CAMK2D** |
| **GO:0032528** | **microvillus organization** | **1** | **22.15384615** | | **0.044195494** | **1.354622006** | **0.025641026** | **RAP2A** |
| **GO:0033014** | **tetrapyrrole biosynthetic process** | **1** | **22.15384615** | | **0.044195494** | **1.354622006** | **0.025641026** | **FXN** |
| **GO:0061003** | **positive regulation of dendritic spine morphogenesis** | **1** | **22.15384615** | | **0.044195494** | **1.354622006** | **0.025641026** | **STAU2** |
| **GO:0070050** | **neuron cellular homeostasis** | **1** | **22.15384615** | | **0.044195494** | **1.354622006** | **0.025641026** | **MAP1A** |
| **GO:1905809** | **negative regulation of synapse organization** | **1** | **22.15384615** | | **0.044195494** | **1.354622006** | **0.025641026** | **PTK2** |
| **GO:0007399** | **nervous system development** | **8** | **1.974919839** | | **0.044249304** | **1.35409356** | **0.205128205** | **PTK2//FKTN//TEAD2//TSC2//MAP1A//FXN//RAP2A//STAU2** |
| **GO:0044249** | **cellular biosynthetic process** | **14** | **1.57231578** | | **0.04454099** | **1.351240135** | **0.358974359** | **NCOA2//DHX29//GRHL1//TEAD2//ZFP426//GMCL1//TFCP2//GTF3C2//NCBP1//FKTN//FXN//TRIM21//TSC2//CAMK2D** |
| **GO:0031346** | **positive regulation of cell projection organization** | **3** | **3.754103637** | | **0.045358819** | **1.343338261** | **0.076923077** | **TSC2//FXN//STAU2** |
| **GO:0050808** | **synapse organization** | **3** | **3.74626626** | | **0.045593126** | **1.341100634** | **0.076923077** | **PTK2//STAU2//TSC2** |
| **GO:0032469** | **endoplasmic reticulum calcium ion homeostasis** | **1** | **21.36263736** | | **0.04579527** | **1.339179377** | **0.025641026** | **CAMK2D** |
| **GO:0051016** | **barbed-end actin filament capping** | **1** | **21.36263736** | | **0.04579527** | **1.339179377** | **0.025641026** | **SVIL** |
| **GO:0061436** | **establishment of skin barrier** | **1** | **21.36263736** | | **0.04579527** | **1.339179377** | **0.025641026** | **GRHL1** |
| **GO:0070979** | **protein K11-linked ubiquitination** | **1** | **21.36263736** | | **0.04579527** | **1.339179377** | **0.025641026** | **CDC27** |
| **GO:0031345** | **negative regulation of cell projection organization** | **2** | **5.779264214** | | **0.046850605** | **1.329284793** | **0.051282051** | **PTK2//TSC2** |
| **GO:0044248** | **cellular catabolic process** | **7** | **2.081052149** | | **0.046949963** | **1.328364747** | **0.179487179** | **NCBP1//VTI1A//PTK2//TRIM21//TSC2//CDC27//MAP1A** |
| **GO:0001666** | **response to hypoxia** | **2** | **5.75147929** | | **0.047256517** | **1.325538293** | **0.051282051** | **CAMK2D//TSC2** |
| **GO:0045292** | **mRNA cis splicing, via spliceosome** | **1** | **20.62599469** | | **0.047392437** | **1.324290962** | **0.025641026** | **NCBP1** |
| **GO:0070296** | **sarcoplasmic reticulum calcium ion transport** | **1** | **20.62599469** | | **0.047392437** | **1.324290962** | **0.025641026** | **CAMK2D** |
| **GO:0031326** | **regulation of cellular biosynthetic process** | **11** | **1.702378346** | | **0.047557043** | **1.32278516** | **0.282051282** | **NCOA2//GRHL1//TEAD2//ZFP426//GMCL1//TFCP2//NCBP1//TRIM21//TSC2//FKTN//CAMK2D** |
| **GO:0048468** | **cell development** | **8** | **1.944425343** | | **0.047829356** | **1.32030547** | **0.205128205** | **CFAP69//MAP1A//TSC2//PTK2//FXN//RAP2A//STAU2//CAMK2D** |
| **GO:1901362** | **organic cyclic compound biosynthetic process** | **11** | **1.69623416** | | **0.048645664** | **1.312955862** | **0.282051282** | **NCOA2//GRHL1//TEAD2//ZFP426//GMCL1//TFCP2//GTF3C2//FXN//TRIM21//TSC2//CAMK2D** |
| **GO:0048731** | **system development** | **13** | **1.594095941** | | **0.04880961** | **1.311494666** | **0.333333333** | **PTK2//TEAD2//FKTN//TSC2//MAP1A//SVIL//AHCTF1//GRHL1//FXN//IQCB1//STAU2//RAP2A//CAMK2D** |
| **GO:0080154** | **regulation of fertilization** | **1** | **19.93846154** | | **0.048986998** | **1.30991917** | **0.025641026** | **CFAP69** |
| **GO:1902751** | **positive regulation of cell cycle G2/M phase transition** | **1** | **19.93846154** | | **0.048986998** | **1.30991917** | **0.025641026** | **CAMK2D** |
| **GO:0008361** | **regulation of cell size** | **2** | **5.616468039** | | **0.049305591** | **1.307103827** | **0.051282051** | **FXN//TSC2** |
| **GO:0032984** | **protein-containing complex disassembly** | **2** | **5.590222861** | | **0.049719273** | **1.303475227** | **0.051282051** | **MAP1A//SVIL** |
| **GO:1902531** | **regulation of intracellular signal transduction** | **6** | **2.224998808** | | **0.049726629** | **1.303410983** | **0.153846154** | **TSC2//PTK2//CAMK2D//TRIM21//RAP2A//FKTN** |
| **GO:0050801** | **ion homeostasis** | **4** | **2.865407646** | | **0.04982908** | **1.302517132** | **0.102564103** | **CAMK2D//FXN//CNNM2//PTK2** |
| **Cellular component** | | | | | | | | |
| **GO.ID** | **Term** | **Count** | **Fold.Enrichment** | | **Pvalue** | **Enrichment.Score** | **Gene.Ratio** | **GENES** |
| **GO:0005829** | **cytosol** | **16** | **2.661577062** | | **0.000105473** | **3.976857002** | **0.41025641** | **DHX29//TSC2//FXN//CAMK2D//PTK2//MAP1A//GRHL1//TRIM21//TFCP2//TEAD2//SVIL//LSAMP//NCBP1//FMNL2//RAP2A//IPO11** |
| **GO:0043229** | **intracellular organelle** | **32** | **1.544084898** | | **0.000160084** | **3.79565152** | **0.820512821** | **AHCTF1//PTK2//CAMK2D//NCOA2//GRHL1//TRIM21//TFCP2//TEAD2//CDC27//TSC2//SVIL//ZFP426//GMCL1//FKTN//STAU2//TMEM120B//NCBP1//CWC27//IPO11//RAB11FIP3//IQCB1//GTF3C2//U2AF1L4//FXN//DHX29//RAP2A//VTI1A//SEC23B//PDIA5//MAP1A//CNNM2//CFAP69** |
| **GO:0043025** | **neuronal cell body** | **7** | **5.793129201** | | **0.000172474** | **3.763276632** | **0.179487179** | **CACNA1E//CAMK2D//MAP1A//NCOA2//TSC2//STAU2//VTI1A** |
| **GO:0005634** | **nucleus** | **23** | **1.921310805** | | **0.00023515** | **3.6286543** | **0.58974359** | **GMCL1//IPO11//TMEM120B//AHCTF1//NCOA2//GRHL1//TRIM21//TFCP2//RAB11FIP3//TEAD2//CDC27//IQCB1//NCBP1//CWC27//GTF3C2//U2AF1L4//PTK2//STAU2//CAMK2D//TSC2//SVIL//ZFP426//FKTN** |
| **GO:0036477** | **somatodendritic compartment** | **8** | **4.603219346** | | **0.000271443** | **3.566320729** | **0.205128205** | **MAP1A//NCOA2//TSC2//STAU2//CAMK2D//VTI1A//PTK2//CACNA1E** |
| **GO:0005622** | **intracellular** | **34** | **1.436591025** | | **0.000285255** | **3.544766154** | **0.871794872** | **AHCTF1//PTK2//CAMK2D//NCOA2//GRHL1//TRIM21//TFCP2//TEAD2//CDC27//TSC2//SVIL//ZFP426//GMCL1//FKTN//STAU2//TMEM120B//NCBP1//CWC27//IPO11//RAB11FIP3//IQCB1//GTF3C2//U2AF1L4//FXN//MAP1A//CFAP69//DHX29//SEC23B//FMNL2//RAP2A//VTI1A//PDIA5//LSAMP//CNNM2** |
| **GO:0043231** | **intracellular membrane-bounded organelle** | **29** | **1.61962532** | | **0.000289973** | **3.53764262** | **0.743589744** | **CAMK2D//PTK2//NCOA2//GRHL1//TRIM21//TFCP2//TEAD2//CDC27//TSC2//SVIL//AHCTF1//ZFP426//GMCL1//FKTN//STAU2//TMEM120B//NCBP1//CWC27//IPO11//RAB11FIP3//IQCB1//GTF3C2//U2AF1L4//FXN//DHX29//VTI1A//SEC23B//PDIA5//CNNM2** |
| **GO:0043226** | **organelle** | **32** | **1.500876737** | | **0.000320719** | **3.49387532** | **0.820512821** | **AHCTF1//PTK2//MAP1A//IQCB1//CAMK2D//NCOA2//GRHL1//TRIM21//TFCP2//TEAD2//CDC27//TSC2//SVIL//ZFP426//GMCL1//FKTN//STAU2//TMEM120B//NCBP1//CWC27//IPO11//RAB11FIP3//GTF3C2//U2AF1L4//FXN//DHX29//RAP2A//VTI1A//SEC23B//PDIA5//CFAP69//CNNM2** |
| **GO:0044297** | **cell body** | **7** | **5.124964606** | | **0.000363874** | **3.439049353** | **0.179487179** | **CAMK2D//MAP1A//NCOA2//TSC2//STAU2//VTI1A//CACNA1E** |
| **GO:0043227** | **membrane-bounded organelle** | **30** | **1.561749191** | | **0.000379841** | **3.420398069** | **0.769230769** | **CAMK2D//PTK2//NCOA2//GRHL1//TRIM21//TFCP2//TEAD2//CDC27//TSC2//SVIL//AHCTF1//ZFP426//GMCL1//FKTN//STAU2//TMEM120B//NCBP1//CWC27//IPO11//RAB11FIP3//IQCB1//GTF3C2//U2AF1L4//FXN//DHX29//RAP2A//VTI1A//SEC23B//PDIA5//CNNM2** |
| **GO:0032991** | **protein-containing complex** | **19** | **2.090568661** | | **0.000457537** | **3.339573479** | **0.487179487** | **GTF3C2//AHCTF1//TFCP2//TEAD2//CDC27//U2AF1L4//NCBP1//MAP1A//CACNA1E//CAMK2D//DHX29//TRIM21//SEC23B//VTI1A//TSC2//CWC27//STAU2//FXN//NCOA2** |
| **GO:0043194** | **axon initial segment** | **2** | **59.66923077** | | **0.00051016** | **3.292293773** | **0.051282051** | **CAMK2D//MAP1A** |
| **GO:0012507** | **ER to Golgi transport vesicle membrane** | **2** | **56.82783883** | | **0.000563264** | **3.249287867** | **0.051282051** | **SEC23B//VTI1A** |
| **GO:0005635** | **nuclear envelope** | **5** | **7.08660698** | | **0.000656523** | **3.18275026** | **0.128205128** | **TMEM120B//AHCTF1//STAU2//GMCL1//IPO11** |
| **GO:0031981** | **nuclear lumen** | **15** | **2.141240339** | | **0.002053857** | **2.687429733** | **0.384615385** | **NCOA2//GRHL1//TRIM21//TFCP2//RAB11FIP3//TEAD2//CDC27//IQCB1//NCBP1//CWC27//GTF3C2//IPO11//PTK2//AHCTF1//GMCL1** |
| **GO:0005654** | **nucleoplasm** | **13** | **2.314831394** | | **0.002310371** | **2.636318217** | **0.333333333** | **PTK2//NCOA2//GRHL1//TRIM21//TFCP2//RAB11FIP3//TEAD2//CDC27//IQCB1//NCBP1//CWC27//GTF3C2//IPO11** |
| **GO:0097730** | **non-motile cilium** | **3** | **11.18798077** | | **0.002431048** | **2.614206545** | **0.076923077** | **MAP1A//IQCB1//CFAP69** |
| **GO:0005856** | **cytoskeleton** | **10** | **2.748467562** | | **0.002487148** | **2.604298291** | **0.256410256** | **PTK2//RAB11FIP3//CDC27//IQCB1//MAP1A//NCOA2//STAU2//SVIL//CFAP69//TSC2** |
| **GO:0055038** | **recycling endosome membrane** | **2** | **25.39116203** | | **0.002820853** | **2.549619522** | **0.051282051** | **RAB11FIP3//RAP2A** |
| **GO:0043005** | **neuron projection** | **8** | **3.11182429** | | **0.003428211** | **2.464932487** | **0.205128205** | **MAP1A//IQCB1//STAU2//NCOA2//TSC2//CAMK2D//PTK2//VTI1A** |
| **GO:0015630** | **microtubule cytoskeleton** | **7** | **3.472025066** | | **0.003484495** | **2.457860152** | **0.179487179** | **RAB11FIP3//CDC27//IQCB1//MAP1A//NCOA2//STAU2//PTK2** |
| **GO:0030134** | **COPII-coated ER to Golgi transport vesicle** | **2** | **21.6979021** | | **0.003842505** | **2.415385605** | **0.051282051** | **VTI1A//SEC23B** |
| **GO:0030660** | **Golgi-associated vesicle membrane** | **2** | **19.56368222** | | **0.004705339** | **2.32740911** | **0.051282051** | **VTI1A//SEC23B** |
| **GO:0014704** | **intercalated disc** | **2** | **19.24813896** | | **0.00485706** | **2.313626529** | **0.051282051** | **CAMK2D//PTK2** |
| **GO:0070013** | **intracellular organelle lumen** | **15** | **1.948277017** | | **0.005295094** | **2.276126353** | **0.384615385** | **NCOA2//GRHL1//TRIM21//TFCP2//RAB11FIP3//TEAD2//CDC27//IQCB1//NCBP1//CWC27//GTF3C2//IPO11//PTK2//AHCTF1//GMCL1** |
| **GO:0031974** | **membrane-enclosed lumen** | **15** | **1.947853017** | | **0.005306339** | **2.275205001** | **0.384615385** | **NCOA2//GRHL1//TRIM21//TFCP2//RAB11FIP3//TEAD2//CDC27//IQCB1//NCBP1//CWC27//GTF3C2//IPO11//PTK2//AHCTF1//GMCL1** |
| **GO:0043233** | **organelle lumen** | **15** | **1.947853017** | | **0.005306339** | **2.275205001** | **0.384615385** | **NCOA2//GRHL1//TRIM21//TFCP2//RAB11FIP3//TEAD2//CDC27//IQCB1//NCBP1//CWC27//GTF3C2//IPO11//PTK2//AHCTF1//GMCL1** |
| **GO:0005874** | **microtubule** | **4** | **5.550626118** | | **0.005673229** | **2.246169674** | **0.102564103** | **CDC27//MAP1A//NCOA2//STAU2** |
| **GO:0005737** | **cytoplasm** | **27** | **1.445942587** | | **0.00573374** | **2.241562018** | **0.692307692** | **FXN//DHX29//NCBP1//TSC2//RAB11FIP3//RAP2A//VTI1A//FKTN//SEC23B//STAU2//PDIA5//NCOA2//CAMK2D//PTK2//MAP1A//GRHL1//TRIM21//TFCP2//TEAD2//SVIL//LSAMP//FMNL2//IPO11//CFAP69//CDC27//AHCTF1//IQCB1** |
| **GO:0043198** | **dendritic shaft** | **2** | **17.29542921** | | **0.005981447** | **2.223193719** | **0.051282051** | **MAP1A//STAU2** |
| **GO:0030425** | **dendrite** | **5** | **4.219889022** | | **0.006250848** | **2.204061096** | **0.128205128** | **PTK2//NCOA2//MAP1A//STAU2//TSC2** |
| **GO:0097447** | **dendritic tree** | **5** | **4.202058505** | | **0.00636173** | **2.19642478** | **0.128205128** | **MAP1A//NCOA2//TSC2//STAU2//PTK2** |
| **GO:0010008** | **endosome membrane** | **3** | **7.885801423** | | **0.0064632** | **2.189552393** | **0.076923077** | **VTI1A//RAB11FIP3//RAP2A** |
| **GO:0045171** | **intercellular bridge** | **2** | **16.57478632** | | **0.006496311** | **2.187333163** | **0.051282051** | **RAB11FIP3//IQCB1** |
| **GO:0098794** | **postsynapse** | **5** | **4.070206737** | | **0.007258555** | **2.139149843** | **0.128205128** | **MAP1A//NCOA2//TSC2//PTK2//CACNA1E** |
| **GO:0030662** | **coated vesicle membrane** | **2** | **15.29980276** | | **0.007584344** | **2.120081962** | **0.051282051** | **VTI1A//SEC23B** |
| **GO:0042995** | **cell projection** | **10** | **2.337220163** | | **0.007949249** | **2.09967389** | **0.256410256** | **MAP1A//IQCB1//CFAP69//PTK2//STAU2//NCOA2//TSC2//CAMK2D//VTI1A//SVIL** |
| **GO:0001750** | **photoreceptor outer segment** | **2** | **14.91730769** | | **0.007964082** | **2.098864257** | **0.051282051** | **MAP1A//IQCB1** |
| **GO:0031965** | **nuclear membrane** | **3** | **7.27673546** | | **0.008056484** | **2.093854462** | **0.076923077** | **TMEM120B//AHCTF1//STAU2** |
| **GO:0044291** | **cell-cell contact zone** | **2** | **14.039819** | | **0.008950197** | **2.048167395** | **0.051282051** | **CAMK2D//PTK2** |
| **GO:0044304** | **main axon** | **2** | **14.039819** | | **0.008950197** | **2.048167395** | **0.051282051** | **CAMK2D//MAP1A** |
| **GO:0012505** | **endomembrane system** | **13** | **1.974293713** | | **0.009302115** | **2.031418299** | **0.333333333** | **GMCL1//IPO11//TMEM120B//AHCTF1//RAB11FIP3//RAP2A//FKTN//SEC23B//STAU2//PDIA5//VTI1A//NCOA2//CAMK2D** |
| **GO:0016363** | **nuclear matrix** | **2** | **13.56118881** | | **0.009566792** | **2.019233669** | **0.051282051** | **AHCTF1//GMCL1** |
| **GO:0045202** | **synapse** | **7** | **2.878598314** | | **0.009571881** | **2.01900271** | **0.179487179** | **VTI1A//MAP1A//NCOA2//TSC2//CAMK2D//PTK2//CACNA1E** |
| **GO:1990904** | **ribonucleoprotein complex** | **5** | **3.669694389** | | **0.011069835** | **1.955858846** | **0.128205128** | **U2AF1L4//DHX29//CWC27//STAU2//NCBP1** |
| **GO:0120025** | **plasma membrane bounded cell projection** | **9** | **2.310770555** | | **0.012968132** | **1.887122585** | **0.230769231** | **MAP1A//IQCB1//CFAP69//PTK2//STAU2//NCOA2//TSC2//CAMK2D//VTI1A** |
| **GO:0099080** | **supramolecular complex** | **6** | **2.934552333** | | **0.015172364** | **1.818946746** | **0.153846154** | **AHCTF1//MAP1A//NCOA2//STAU2//CDC27//SVIL** |
| **GO:0034399** | **nuclear periphery** | **2** | **10.5609258** | | **0.015404982** | **1.812338796** | **0.051282051** | **AHCTF1//GMCL1** |
| **GO:0097733** | **photoreceptor cell cilium** | **2** | **10.28779841** | | **0.016186563** | **1.790845357** | **0.051282051** | **MAP1A//IQCB1** |
| **GO:0005845** | **mRNA cap binding complex** | **1** | **59.66923077** | | **0.016636423** | **1.778940048** | **0.025641026** | **NCBP1** |
| **GO:0031080** | **nuclear pore outer ring** | **1** | **59.66923077** | | **0.016636423** | **1.778940048** | **0.025641026** | **AHCTF1** |
| **GO:0034518** | **RNA cap binding complex** | **1** | **59.66923077** | | **0.016636423** | **1.778940048** | **0.025641026** | **NCBP1** |
| **GO:0090576** | **RNA polymerase III transcription regulator complex** | **1** | **59.66923077** | | **0.016636423** | **1.778940048** | **0.025641026** | **GTF3C2** |
| **GO:0099512** | **supramolecular fiber** | **5** | **3.271339406** | | **0.017459666** | **1.757964066** | **0.128205128** | **MAP1A//NCOA2//STAU2//CDC27//SVIL** |
| **GO:0099081** | **supramolecular polymer** | **5** | **3.246421696** | | **0.017988145** | **1.745013622** | **0.128205128** | **MAP1A//NCOA2//STAU2//CDC27//SVIL** |
| **GO:0097731** | **9+0 non-motile cilium** | **2** | **9.702313946** | | **0.018075** | **1.742921696** | **0.051282051** | **MAP1A//IQCB1** |
| **GO:0030054** | **cell junction** | **8** | **2.311640901** | | **0.019354801** | **1.713211286** | **0.205128205** | **PTK2//VTI1A//MAP1A//NCOA2//TSC2//CAMK2D//CACNA1E//SVIL** |
| **GO:0005798** | **Golgi-associated vesicle** | **2** | **8.58550083** | | **0.022721407** | **1.643564785** | **0.051282051** | **VTI1A//SEC23B** |
| **GO:0030127** | **COPII vesicle coat** | **1** | **42.62087912** | | **0.023215211** | **1.634227365** | **0.025641026** | **SEC23B** |
| **GO:1990124** | **messenger ribonucleoprotein complex** | **1** | **42.62087912** | | **0.023215211** | **1.634227365** | **0.025641026** | **STAU2** |
| **GO:0048471** | **perinuclear region of cytoplasm** | **4** | **3.583737584** | | **0.024771225** | **1.606052524** | **0.102564103** | **CAMK2D//TSC2//SEC23B//VTI1A** |
| **GO:0030658** | **transport vesicle membrane** | **2** | **8.11826269** | | **0.025210428** | **1.598419782** | **0.051282051** | **VTI1A//SEC23B** |
| **GO:0005929** | **cilium** | **4** | **3.551739927** | | **0.025495863** | **1.593530281** | **0.102564103** | **MAP1A//IQCB1//CFAP69//PTK2** |
| **GO:0031090** | **organelle membrane** | **8** | **2.192714038** | | **0.025741999** | **1.589357737** | **0.205128205** | **TMEM120B//VTI1A//SEC23B//FKTN//AHCTF1//STAU2//RAB11FIP3//RAP2A** |
| **GO:0055037** | **recycling endosome** | **2** | **7.799899447** | | **0.027147113** | **1.566276357** | **0.051282051** | **RAB11FIP3//RAP2A** |
| **GO:0099513** | **polymeric cytoskeletal fiber** | **4** | **3.459085842** | | **0.027746923** | **1.556785169** | **0.102564103** | **MAP1A//NCOA2//STAU2//CDC27** |
| **GO:0014069** | **postsynaptic density** | **3** | **4.475192308** | | **0.029181926** | **1.534886055** | **0.076923077** | **MAP1A//NCOA2//TSC2** |
| **GO:0032279** | **asymmetric synapse** | **3** | **4.41994302** | | **0.030120899** | **1.521132063** | **0.076923077** | **MAP1A//NCOA2//TSC2** |
| **GO:0031461** | **cullin-RING ubiquitin ligase complex** | **2** | **7.321378008** | | **0.030504192** | **1.515640469** | **0.051282051** | **CDC27//TRIM21** |
| **GO:0042383** | **sarcolemma** | **2** | **7.27673546** | | **0.0308486** | **1.510764534** | **0.051282051** | **CAMK2D//PTK2** |
| **GO:0005815** | **microtubule organizing center** | **4** | **3.338138784** | | **0.031067091** | **1.507699413** | **0.102564103** | **RAB11FIP3//CDC27//IQCB1//PTK2** |
| **GO:0043034** | **costamere** | **1** | **31.4048583** | | **0.031378412** | **1.503369041** | **0.025641026** | **SVIL** |
| **GO:0030424** | **axon** | **4** | **3.296642584** | | **0.032317943** | **1.490556289** | **0.102564103** | **TSC2//CAMK2D//MAP1A//STAU2** |
| **GO:0031967** | **organelle envelope** | **5** | **2.770159274** | | **0.032885024** | **1.48300184** | **0.128205128** | **GMCL1//IPO11//TMEM120B//AHCTF1//STAU2** |
| **GO:0031975** | **envelope** | **5** | **2.770159274** | | **0.032885024** | **1.48300184** | **0.128205128** | **GMCL1//IPO11//TMEM120B//AHCTF1//STAU2** |
| **GO:0098984** | **neuron to neuron synapse** | **3** | **4.134126843** | | **0.035673706** | **1.447651766** | **0.076923077** | **MAP1A//NCOA2//TSC2** |
| **GO:0005680** | **anaphase-promoting complex** | **1** | **27.12237762** | | **0.036244385** | **1.440759266** | **0.025641026** | **CDC27** |
| **GO:0099572** | **postsynaptic specialization** | **3** | **4.077623971** | | **0.036928302** | **1.43264066** | **0.076923077** | **MAP1A//NCOA2//TSC2** |
| **GO:0098588** | **bounding membrane of organelle** | **5** | **2.65905663** | | **0.038228606** | **1.417611534** | **0.128205128** | **VTI1A//SEC23B//FKTN//RAB11FIP3//RAP2A** |
| **GO:0030135** | **coated vesicle** | **2** | **6.215544872** | | **0.041103397** | **1.386122284** | **0.051282051** | **VTI1A//SEC23B** |
| **GO:0005667** | **transcription regulator complex** | **3** | **3.891471572** | | **0.041497631** | **1.381976698** | **0.076923077** | **GTF3C2//TEAD2//TFCP2** |
| **GO:0043197** | **dendritic spine** | **2** | **6.119921105** | | **0.042269321** | **1.373974732** | **0.051282051** | **PTK2//NCOA2** |
| **GO:0005681** | **spliceosomal complex** | **2** | **6.088697017** | | **0.042660733** | **1.369971688** | **0.051282051** | **CWC27//U2AF1L4** |
| **GO:0098805** | **whole membrane** | **5** | **2.556522312** | | **0.044084642** | **1.355712679** | **0.128205128** | **TSC2//VTI1A//SEC23B//RAB11FIP3//RAP2A** |
| **GO:0044309** | **neuron spine** | **2** | **5.966923077** | | **0.044240091** | **1.354183984** | **0.051282051** | **PTK2//NCOA2** |
| **Molecular function** | | | | | | | | |
| **GO.ID** | **Term** | **Count** | **Fold.Enrichment** | | **Pvalue** | **Enrichment.Score** | **Gene.Ratio** | **GENES** |
| **GO:0019899** | **enzyme binding** | **12** | **3.107399877** | | **0.000247936** | **3.605660349** | **0.324324324** | **PTK2//IPO11//FMNL2//RAB11FIP3//TRIM21//TSC2//CDC27//CAMK2D//STAU2//NCOA2//FXN//IQCB1** |
| **GO:0044877** | **protein-containing complex binding** | **8** | **3.305060961** | | **0.002305294** | **2.637273752** | **0.216216216** | **PTK2//MAP1A//STAU2//DHX29//SVIL//FMNL2//NCOA2//TSC2** |
| **GO:0005515** | **protein binding** | **24** | **1.578044239** | | **0.002992372** | **2.523984467** | **0.648648649** | **VTI1A//TEAD2//PTK2//MAP1A//SVIL//FMNL2//NCOA2//CAMK2D//IQCB1//TFCP2//IPO11//RAB11FIP3//STAU2//FXN//TRIM21//TSC2//CDC27//GRHL1//CACNA1E//U2AF1L4//GMCL1//SEC23B//NCBP1//RAP2A** |
| **GO:0046983** | **protein dimerization activity** | **6** | **3.82523835** | | **0.004309095** | **2.365613922** | **0.162162162** | **CAMK2D//GRHL1//TRIM21//RAB11FIP3//TSC2//NCOA2** |
| **GO:0042803** | **protein homodimerization activity** | **5** | **4.572206535** | | **0.004427157** | **2.353875107** | **0.135135135** | **CAMK2D//GRHL1//TRIM21//RAB11FIP3//TSC2** |
| **GO:0008092** | **cytoskeletal protein binding** | **6** | **3.751075565** | | **0.004739806** | **2.324239451** | **0.162162162** | **PTK2//MAP1A//SVIL//FMNL2//STAU2//CAMK2D** |
| **GO:0031267** | **small GTPase binding** | **4** | **5.779959204** | | **0.004893135** | **2.310412773** | **0.108108108** | **IPO11//FMNL2//RAB11FIP3//TSC2** |
| **GO:0003779** | **actin binding** | **4** | **5.646780421** | | **0.005311264** | **2.274802129** | **0.108108108** | **SVIL//FMNL2//PTK2//MAP1A** |
| **GO:0019902** | **phosphatase binding** | **3** | **8.242273664** | | **0.005703935** | **2.243825445** | **0.081081081** | **PTK2//CDC27//TSC2** |
| **GO:0003676** | **nucleic acid binding** | **12** | **2.150367975** | | **0.006345862** | **2.197509392** | **0.324324324** | **NCBP1//GRHL1//NCOA2//TFCP2//TEAD2//ZFP426//TRIM21//AHCTF1//GTF3C2//DHX29//STAU2//U2AF1L4** |
| **GO:1901363** | **heterocyclic compound binding** | **16** | **1.833672056** | | **0.00646706** | **2.189293134** | **0.432432432** | **CAMK2D//PTK2//DHX29//RAP2A//NCBP1//GRHL1//NCOA2//TFCP2//TEAD2//ZFP426//TRIM21//AHCTF1//GTF3C2//STAU2//CNNM2//U2AF1L4** |
| **GO:0097159** | **organic cyclic compound binding** | **16** | **1.799671528** | | **0.007821817** | **2.106692339** | **0.432432432** | **CAMK2D//PTK2//DHX29//RAP2A//NCBP1//GRHL1//NCOA2//TFCP2//TEAD2//ZFP426//TRIM21//AHCTF1//GTF3C2//STAU2//CNNM2//U2AF1L4** |
| **GO:0051020** | **GTPase binding** | **4** | **4.685856028** | | **0.010129245** | **1.994422917** | **0.108108108** | **IPO11//FMNL2//RAB11FIP3//TSC2** |
| **GO:0017162** | **aryl hydrocarbon receptor binding** | **1** | **61.26756757** | | **0.016205682** | **1.79033269** | **0.027027027** | **NCOA2** |
| **GO:0140104** | **molecular carrier activity** | **1** | **61.26756757** | | **0.016205682** | **1.79033269** | **0.027027027** | **FXN** |
| **GO:0008494** | **translation activator activity** | **1** | **55.6977887** | | **0.017812125** | **1.74928427** | **0.027027027** | **DHX29** |
| **GO:0005488** | **binding** | **29** | **1.291906827** | | **0.017989755** | **1.744974743** | **0.783783784** | **VTI1A//CAMK2D//PTK2//DHX29//RAP2A//NCBP1//GRHL1//NCOA2//TFCP2//TEAD2//ZFP426//TRIM21//AHCTF1//GTF3C2//STAU2//MAP1A//SVIL//FMNL2//CACNA1E//RAB11FIP3//TSC2//U2AF1L4//GMCL1//SEC23B//IQCB1//IPO11//CNNM2//FXN//CDC27** |
| **GO:0047485** | **protein N-terminus binding** | **2** | **9.498847685** | | **0.018791946** | **1.726028243** | **0.054054054** | **CACNA1E//TSC2** |
| **GO:0008093** | **cytoskeletal anchor activity** | **1** | **51.05630631** | | **0.019416015** | **1.711839894** | **0.027027027** | **MAP1A** |
| **GO:0015037** | **peptide disulfide oxidoreductase activity** | **1** | **51.05630631** | | **0.019416015** | **1.711839894** | **0.027027027** | **PDIA5** |
| **GO:0000340** | **RNA 7-methylguanosine cap binding** | **1** | **47.12889813** | | **0.021017357** | **1.67742189** | **0.027027027** | **NCBP1** |
| **GO:0004322** | **ferroxidase activity** | **1** | **47.12889813** | | **0.021017357** | **1.67742189** | **0.027027027** | **FXN** |
| **GO:0016724** | **oxidoreductase activity, oxidizing metal ions, oxygen as acceptor** | **1** | **47.12889813** | | **0.021017357** | **1.67742189** | **0.027027027** | **FXN** |
| **GO:0099511** | **voltage-gated calcium channel activity involved in regulation of cytosolic calcium levels** | **1** | **47.12889813** | | **0.021017357** | **1.67742189** | **0.027027027** | **CACNA1E** |
| **GO:0099626** | **voltage-gated calcium channel activity involved in regulation of presynaptic cytosolic calcium levels** | **1** | **47.12889813** | | **0.021017357** | **1.67742189** | **0.027027027** | **CACNA1E** |
| **GO:0031072** | **heat shock protein binding** | **2** | **8.392817475** | | **0.023680473** | **1.625609627** | **0.054054054** | **STAU2//TSC2** |
| **GO:0043021** | **ribonucleoprotein complex binding** | **2** | **8.392817475** | | **0.023680473** | **1.625609627** | **0.054054054** | **STAU2//DHX29** |
| **GO:0001162** | **RNA polymerase II intronic transcription regulatory region sequence-specific DNA binding** | **1** | **40.84504505** | | **0.024212412** | **1.615961945** | **0.027027027** | **NCOA2** |
| **GO:0008432** | **JUN kinase binding** | **1** | **40.84504505** | | **0.024212412** | **1.615961945** | **0.027027027** | **PTK2** |
| **GO:0000339** | **RNA cap binding** | **1** | **38.29222973** | | **0.025806132** | **1.58827708** | **0.027027027** | **NCBP1** |
| **GO:0015095** | **magnesium ion transmembrane transporter activity** | **1** | **38.29222973** | | **0.025806132** | **1.58827708** | **0.027027027** | **CNNM2** |
| **GO:0043024** | **ribosomal small subunit binding** | **1** | **38.29222973** | | **0.025806132** | **1.58827708** | **0.027027027** | **DHX29** |
| **GO:0016853** | **isomerase activity** | **2** | **8.008832362** | | **0.025829683** | **1.587880924** | **0.054054054** | **CWC27//PDIA5** |
| **GO:0003756** | **protein disulfide isomerase activity** | **1** | **36.03974563** | | **0.02739732** | **1.562291919** | **0.027027027** | **PDIA5** |
| **GO:0016864** | **intramolecular oxidoreductase activity, transposing S-S bonds** | **1** | **36.03974563** | | **0.02739732** | **1.562291919** | **0.027027027** | **PDIA5** |
| **GO:0000978** | **RNA polymerase II cis-regulatory region sequence-specific DNA binding** | **5** | **2.876411623** | | **0.028395291** | **1.54675367** | **0.135135135** | **NCOA2//GRHL1//TFCP2//TEAD2//ZFP426** |
| **GO:0017016** | **Ras GTPase binding** | **3** | **4.493953611** | | **0.028803286** | **1.540557966** | **0.081081081** | **IPO11//FMNL2//RAB11FIP3** |
| **GO:0008199** | **ferric iron binding** | **1** | **34.03753754** | | **0.028985979** | **1.537812031** | **0.027027027** | **FXN** |
| **GO:0061608** | **nuclear import signal receptor activity** | **1** | **34.03753754** | | **0.028985979** | **1.537812031** | **0.027027027** | **IPO11** |
| **GO:0003677** | **DNA binding** | **8** | **2.117237756** | | **0.030538121** | **1.515157692** | **0.216216216** | **GRHL1//NCOA2//TFCP2//TEAD2//ZFP426//TRIM21//AHCTF1//GTF3C2** |
| **GO:0001161** | **intronic transcription regulatory region sequence-specific DNA binding** | **1** | **32.24608819** | | **0.030572113** | **1.514674549** | **0.027027027** | **NCOA2** |
| **GO:0070182** | **DNA polymerase binding** | **1** | **32.24608819** | | **0.030572113** | **1.514674549** | **0.027027027** | **NCOA2** |
| **GO:0000987** | **cis-regulatory region sequence-specific DNA binding** | **5** | **2.810438879** | | **0.030973194** | **1.509014007** | **0.135135135** | **NCOA2//GRHL1//TFCP2//TEAD2//ZFP426** |
| **GO:0035259** | **glucocorticoid receptor binding** | **1** | **30.63378378** | | **0.032155726** | **1.492741687** | **0.027027027** | **NCOA2** |
| **GO:0019903** | **protein phosphatase binding** | **2** | **6.962223587** | | **0.03342048** | **1.475987314** | **0.054054054** | **PTK2//CDC27** |
| **GO:0016722** | **oxidoreductase activity, oxidizing metal ions** | **1** | **27.84889435** | | **0.035315404** | **1.452035823** | **0.027027027** | **FXN** |
| **GO:0046965** | **retinoid X receptor binding** | **1** | **27.84889435** | | **0.035315404** | **1.452035823** | **0.027027027** | **NCOA2** |
| **GO:0050998** | **nitric-oxide synthase binding** | **1** | **27.84889435** | | **0.035315404** | **1.452035823** | **0.027027027** | **CAMK2D** |
| **GO:0003723** | **RNA binding** | **5** | **2.670774523** | | **0.03739725** | **1.427160337** | **0.135135135** | **NCBP1//STAU2//DHX29//U2AF1L4//TRIM21** |
| **GO:0004683** | **calmodulin-dependent protein kinase activity** | **1** | **25.52815315** | | **0.038465045** | **1.414933757** | **0.027027027** | **CAMK2D** |
| **GO:0005516** | **calmodulin binding** | **2** | **6.382038288** | | **0.039153405** | **1.407230462** | **0.054054054** | **CAMK2D//IQCB1** |
| **GO:0051537** | **2 iron, 2 sulfur cluster binding** | **1** | **23.56444906** | | **0.041604679** | **1.38085782** | **0.027027027** | **FXN** |
| **GO:0046966** | **thyroid hormone receptor binding** | **1** | **22.69169169** | | **0.043170754** | **1.364810366** | **0.027027027** | **NCOA2** |
| **GO:0001223** | **transcription coactivator binding** | **1** | **21.88127413** | | **0.044734339** | **1.349358979** | **0.027027027** | **TEAD2** |
| **GO:0140142** | **nucleocytoplasmic carrier activity** | **1** | **21.88127413** | | **0.044734339** | **1.349358979** | **0.027027027** | **IPO11** |
| **GO:0051015** | **actin filament binding** | **2** | **5.835006435** | | **0.046016054** | **1.337090622** | **0.054054054** | **SVIL//FMNL2** |
| **GO:0051959** | **dynein light intermediate chain binding** | **1** | **19.76373147** | | **0.049410191** | **1.306183469** | **0.027027027** | **RAB11FIP3** |
| **GO:0071889** | **14-3-3 protein binding** | **1** | **19.76373147** | | **0.049410191** | **1.306183469** | **0.027027027** | **TSC2** |

## 1.4 Supplementary Table4

The KEGG enrich annotation of changed proteins in HFD-fed mice vs control.

| KEGG pathway analysis of down-regulated circRNAs | | | | | | | |
| --- | --- | --- | --- | --- | --- | --- | --- |
| PathwayID | Definition | Fisher-Pvalue | Count | FDR | Enrichment_Score | GeneRatio | Genes |
| mmu04141 | Protein processing in endoplasmic reticulum - Mus musculus (mouse) | 0.002080708 | 163 | 0.6866336 | 2.681789 | 0.5 | PDIA3//SEC24D |
| KEGG pathway analysis of up-regulated circRNAs | | | | | | | |
| PathwayID | Definition | Fisher-Pvalue | Count | FDR | Enrichment_Score | GeneRatio | Genes |
| mmu04012 | ErbB signaling pathway - Mus musculus (mouse) | 0.01030996 | 84 | 1 | 1.986743 | 0.125 | CAMK2D//PTK2 |
| mmu04919 | Thyroid hormone signaling pathway - Mus musculus (mouse) | 0.01968092 | 118 | 1 | 1.705955 | 0.125 | NCOA2//TSC2 |
| mmu04114 | Oocyte meiosis - Mus musculus (mouse) | 0.0199958 | 119 | 1 | 1.699061 | 0.125 | CAMK2D//CDC27 |
| mmu03040 | Spliceosome - Mus musculus (mouse) | 0.02462569 | 133 | 1 | 1.608612 | 0.125 | NCBP1//U2AF1L4 |
| mmu04360 | Axon guidance - Mus musculus (mouse) | 0.04296842 | 180 | 1 | 1.366851 | 0.125 | CAMK2D//PTK2 |
| mmu04020 | Calcium signaling pathway - Mus musculus (mouse) | 0.04873323 | 193 | 1 | 1.312175 | 0.125 | CACNA1E//CAMK2D |

## 1.5 Supplementary Table5

CeRNA network target

| **mmu-circRNA-004797 related ceRNA analysis** | | | | | | | | | | |
| --- | --- | --- | --- | --- | --- | --- | --- | --- | --- | --- |
| **SeqName** | **CeNames** | **CeSymbols** | **CeTypes** | **Pvalues** | **FDR** | **Rank** | **Common**  **MirnasNum** | **CeMirnas**  **Num** | **CeMirna**  **Coverage** | **CommonMirnas** |
| **mmu_circRNA_004797** | **ENSMUST00000029611** | **Lef1** | **protein_coding** | **0.000893387** | **0.005879883** | **1831** | **3** | **30** | **0.1** | **mmu-miR-103-2-5p,mmu-miR-107-5p,mmu-miR-103-1-5p** |
| **mmu_circRNA_004797** | **ENSMUST00000053748** | **Epb41l2** | **protein_coding** | **0.000807227** | **0.005501916** | **1743** | **3** | **29** | **0.103448276** | **mmu-miR-103-2-5p,mmu-miR-107-5p,mmu-miR-103-1-5p** |
| **mmu_circRNA_004797** | **ENSMUST00000068681** | **Ngef** | **protein_coding** | **0.000807227** | **0.005501916** | **1764** | **3** | **29** | **0.103448276** | **mmu-miR-103-2-5p,mmu-miR-107-5p,mmu-miR-103-1-5p** |
| **mmu_circRNA_004797** | **ENSMUST00000118163** | **Dmxl2** | **protein_coding** | **0.00040061** | **0.003391554** | **1395** | **3** | **23** | **0.130434783** | **mmu-miR-103-2-5p,mmu-miR-107-5p,mmu-miR-103-1-5p** |
| **mmu_circRNA_004797** | **ENSMUST00000093193** | **Dock2** | **protein_coding** | **0.000303235** | **0.002814017** | **1269** | **3** | **21** | **0.142857143** | **mmu-miR-103-2-5p,mmu-miR-107-5p,mmu-miR-103-1-5p** |
| **mmu_circRNA_004797** | **ENSMUST00000199534** | **Ldb2** | **protein_coding** | **0.0001575** | **0.001828755** | **1023** | **3** | **17** | **0.176470588** | **mmu-miR-103-2-5p,mmu-miR-107-5p,mmu-miR-103-1-5p** |
| **mmu_circRNA_004797** | **ENSMUST00000172947** | **D7Ertd443e** | **protein_coding** | **0.000106219** | **0.001431762** | **871** | **3** | **15** | **0.2** | **mmu-miR-103-2-5p,mmu-miR-107-5p,mmu-miR-103-1-5p** |
| **mmu_circRNA_004797** | **ENSMUST00000042246** | **Shc4** | **protein_coding** | **0.000102047** | **0.001431762** | **844** | **4** | **40** | **0.1** | **mmu-miR-103-2-5p,mmu-miR-107-5p,mmu-miR-103-1-5p,mmu-miR-29b-3p** |
| **mmu_circRNA_004797** | **ENSMUST00000033824** | **Lamp1** | **protein_coding** | **8.53E-05** | **0.00125053** | **820** | **3** | **14** | **0.214285714** | **mmu-miR-103-2-5p,mmu-miR-107-5p,mmu-miR-103-1-5p** |
| **mmu_circRNA_004797** | **ENSMUST00000034115** | **Pou4f2** | **protein_coding** | **5.96E-05** | **0.00103736** | **689** | **4** | **35** | **0.114285714** | **mmu-miR-103-2-5p,mmu-miR-107-5p,mmu-miR-6958-5p,mmu-miR-103-1-5p** |
| **mmu_circRNA_004797** | **ENSMUST00000162349** | **Apbb2** | **protein_coding** | **3.91E-05** | **0.000802836** | **544** | **3** | **11** | **0.272727273** | **mmu-miR-103-2-5p,mmu-miR-107-5p,mmu-miR-103-1-5p** |
| **mmu_circRNA_004797** | **ENSMUST00000002588** | **Tiam1** | **protein_coding** | **3.18E-05** | **0.0007389** | **518** | **4** | **30** | **0.133333333** | **mmu-miR-103-2-5p,mmu-miR-107-5p,mmu-miR-670-3p,mmu-miR-103-1-5p** |
| **mmu_circRNA_004797** | **ENSMUST00000033053** | **Itgax** | **protein_coding** | **2.86E-05** | **0.000670575** | **498** | **3** | **10** | **0.3** | **mmu-miR-103-2-5p,mmu-miR-107-5p,mmu-miR-103-1-5p** |
| **mmu_circRNA_004797** | **ENSMUST00000028749** | **Capn3** | **protein_coding** | **1.34E-05** | **0.000433473** | **351** | **3** | **8** | **0.375** | **mmu-miR-103-2-5p,mmu-miR-107-5p,mmu-miR-103-1-5p** |
| **mmu_circRNA_004797** | **ENSMUST00000025166** | **Cdh2** | **protein_coding** | **9.84E-06** | **0.000364305** | **325** | **5** | **49** | **0.102040816** | **mmu-miR-30c-1-3p,mmu-miR-103-2-5p,mmu-miR-107-5p,mmu-miR-30c-2-3p,mmu-miR-103-1-5p** |
| **mmu_circRNA_004797** | **ENSMUST00000114234** | **Traf2** | **protein_coding** | **0.003182502** | **0.013860541** | **2769** | **2** | **13** | **0.153846154** | **mmu-miR-103-2-5p,mmu-miR-103-1-5p** |
| **mmu_circRNA_004797** | **ENSMUST00000223097** | **Ccdc88c** | **protein_coding** | **0.000876788** | **0.005873164** | **1789** | **2** | **7** | **0.285714286** | **mmu-miR-103-2-5p,mmu-miR-103-1-5p** |
| **mmu_circRNA_004797** | **ENSMUST00000061772** | **Rbm15** | **protein_coding** | **0.000456039** | **0.003672667** | **1474** | **3** | **24** | **0.125** | **mmu-miR-103-2-5p,mmu-miR-670-3p,mmu-miR-103-1-5p** |
| **mmu_circRNA_004797** | **ENSMUST00000037423** | **Ovol2** | **protein_coding** | **0.000420737** | **0.003505363** | **1447** | **2** | **5** | **0.4** | **mmu-miR-103-2-5p,mmu-miR-103-1-5p** |
| **mmu_circRNA_004797** | **ENSMUST00000090856** | **Hrnr** | **protein_coding** | **5.20E-05** | **0.000944204** | **625** | **3** | **12** | **0.25** | **mmu-miR-103-2-5p,mmu-miR-107-5p,mmu-miR-103-1-5p** |
| **mmu-circRNA-21040 related ceRNA analysis** | | | | | | | | | | |
| **SeqName** | **CeNames** | **CeSymbols** | **CeTypes** | **Pvalues** | **FDR** | **Rank** | **CommonMirnasNum** | **CeMirnasNum** | **CeMirnaCoverage** | **CommonMirnas** |
| **mmu_circRNA_21040** | **ENSMUST00000103105** | **Aoc3** | **protein_coding** | **0.011677868** | **0.086032641** | **6542** | **11** | **106** | **0.103773585** | **mmu-miR-7681-3p,mmu-miR-7087-3p,mmu-miR-1249-5p,mmu-miR-6962-5p,mmu-miR-6337,mmu-miR-6965-5p,**  **mmu-miR-7047-5p,**  **mmu-miR-6967-5p,mmu-miR-7033-5p,mmu-miR-5114,mmu-miR-6950-5p** |
| **mmu_circRNA_21040** | **ENSMUST00000032557** | **Mark2** | **protein_coding** | **0.009377912** | **0.076055876** | **5929** | **10** | **89** | **0.112359551** | **mmu-miR-6955-5p,mmu-miR-1249-5p,mmu-miR-7073-5p,mmu-miR-6965-5p,mmu-miR-328-5p,mmu-miR-7047-5p,**  **mmu-miR-6967-5p,**  **mmu-miR-6954-5p,mmu-miR-6950-5p,mmu-miR-7035-3p** |
| **mmu_circRNA_21040** | **ENSMUST00000184945** | **Nav2** | **protein_coding** | **0.003985698** | **0.045206209** | **4255** | **11** | **92** | **0.119565217** | **mmu-miR-1249-5p,mmu-miR-29a-3p,mmu-miR-8104,mmu-miR-5107-5p,mmu-miR-5627-3p,mmu-miR-6965-5p,**  **mmu-miR-29b-3p,**  **mmu-miR-7047-5p,mmu-miR-7066-5p,mmu-miR-7229-3p,mmu-miR-29c-3p** |
| **mmu_circRNA_21040** | **ENSMUST00000023291** | **Mapk8ip2** | **protein_coding** | **0.003982787** | **0.045206209** | **4239** | **10** | **79** | **0.126582278** | **mmu-miR-1249-5p,mmu-miR-6337,mmu-miR-6916-5p,mmu-miR-7061-3p,mmu-miR-6965-5p,mmu-miR-188-5p,**  **mmu-miR-3109-3p,mmu-miR-6904-5p,mmu-miR-6914-5p,mmu-miR-6950-5p** |
| **mmu_circRNA_21040** | **ENSMUST00000114787** | **Stxbp5l** | **protein_coding** | **0.002921196** | **0.038445511** | **3651** | **14** | **129** | **0.108527132** | **mmu-miR-7681-3p,mmu-miR-1249-5p,mmu-miR-7073-5p,mmu-miR-5710,mmu-miR-29a-3p,mmu-miR-877-3p,**  **mmu-miR-6916-5p,mmu-miR-6965-5p,mmu-miR-29b-3p,mmu-miR-7229-3p,mmu-miR-6904-5p,mmu-miR-29c-3p,**  **mmu-miR-881-3p,mmu-miR-6365** |
| **mmu_circRNA_21040** | **ENSMUST00000017458** | **Mpp2** | **protein_coding** | **0.002670181** | **0.035923913** | **3586** | **13** | **114** | **0.114035088** | **mmu-miR-1249-5p,mmu-miR-6916-5p,mmu-miR-705,mmu-miR-148a-3p,mmu-miR-6965-5p,mmu-miR-7047-5p**  **,mmu-miR-1941-5p,mmu-miR-6967-5p,mmu-miR-6922-5p,mmu-miR-6993-5p,mmu-miR-7008-5p,mmu-miR-6935-5p,**  **mmu-miR-7033-5p** |
| **mmu_circRNA_21040** | **ENSMUST00000003461** | **Ogdh** | **protein_coding** | **0.002190346** | **0.031818355** | **3314** | **10** | **73** | **0.136986301** | **mmu-miR-1249-5p,mmu-miR-3474,mmu-miR-6337,mmu-miR-717,mmu-miR-6965-5p,mmu-miR-328-5p,**  **mmu-miR-7047-5p,mmu-miR-7033-5p,mmu-miR-6945-5p,mmu-miR-3110-3p** |
| **mmu_circRNA_21040** | **ENSMUST00000120638** | **Syn3** | **protein_coding** | **0.001765483** | **0.028161506** | **3010** | **13** | **109** | **0.119266055** | **mmu-miR-6955-5p,mmu-miR-1249-5p,mmu-miR-7073-5p,mmu-miR-3474,mmu-miR-29a-3p,mmu-miR-6337,**  **mmu-miR-10b-5p,mmu-miR-10a-5p,mmu-miR-6965-5p,mmu-miR-29b-3p,mmu-miR-3109-3p,mmu-miR-6945-5p,**  **mmu-miR-29c-3p** |
| **mmu_circRNA_21040** | **ENSMUST00000046587** | **Scamp5** | **protein_coding** | **0.001131791** | **0.021299733** | **2563** | **13** | **104** | **0.125** | **mmu-miR-1249-5p,mmu-miR-3474,mmu-miR-8113,mmu-miR-6916-5p,mmu-miR-6961-5p,mmu-miR-148a-3p,**  **mmu-miR-6965-5p,mmu-miR-328-5p,mmu-miR-7047-5p,mmu-miR-145a-3p,mmu-miR-6967-5p,mmu-miR-7229-3p,**  **mmu-miR-6954-5p** |
| **mmu_circRNA_21040** | **ENSMUST00000179865** | **Canx** | **protein_coding** | **0.001114955** | **0.021085106** | **2537** | **10** | **67** | **0.149253731** | **mmu-miR-1249-5p,mmu-miR-103-2-5p,mmu-miR-6962-5p,mmu-miR-1931,mmu-miR-705,mmu-miR-103-1-5p,**  **mmu-miR-2183,mmu-miR-6965-5p,mmu-miR-7047-5p,mmu-miR-6922-5p** |
| **mmu_circRNA_21040** | **ENSMUST00000038495** | **Crp** | **protein_coding** | **0.000728433** | **0.016174297** | **2174** | **12** | **87** | **0.137931034** | **mmu-miR-7032-3p,mmu-miR-1249-5p,mmu-miR-6960-5p,mmu-miR-29a-3p,mmu-miR-6965-5p,mmu-miR-29b-3p,**  **mmu-miR-7047-5p,mmu-miR-1941-5p,mmu-miR-6967-5p,mmu-miR-7239-5p,mmu-miR-7229-3p,mmu-miR-29c-3p** |
| **mmu_circRNA_21040** | **ENSMUST00000069520** | **Syp** | **protein_coding** | **0.000724333** | **0.016142655** | **2155** | **11** | **75** | **0.146666667** | **mmu-miR-1249-5p,mmu-miR-7073-5p,mmu-miR-877-3p,mmu-miR-717,mmu-miR-5107-5p,mmu-miR-6965-5p,**  **mmu-miR-328-5p,mmu-miR-7047-5p,mmu-miR-6967-5p,mmu-miR-6950-3p,mmu-miR-6954-5p** |
| **mmu_circRNA_21040** | **ENSMUST00000002677** | **Axl** | **protein_coding** | **0.000592026** | **0.014196863** | **2008** | **10** | **62** | **0.161290323** | **mmu-miR-1249-5p,mmu-miR-3474,mmu-miR-7009-5p,mmu-miR-7061-3p,mmu-miR-6965-5p,mmu-miR-188-5p,**  **mmu-miR-7047-5p,mmu-miR-6950-3p,mmu-miR-6904-5p,mmu-miR-5114** |
| **mmu_circRNA_21040** | **ENSMUST00000211508** | **Olfr488** | **protein_coding** | **0.000586058** | **0.014138024** | **1998** | **12** | **85** | **0.141176471** | **mmu-miR-7681-3p,mmu-miR-7093-5p,mmu-miR-1249-5p,mmu-miR-6960-5p,mmu-miR-8113,mmu-miR-6962-5p**  **,mmu-miR-6916-5p,mmu-miR-877-5p,mmu-miR-6965-5p,mmu-miR-7047-5p,mmu-miR-6967-5p,mmu-miR-3109-3p** |
| **mmu_circRNA_21040** | **ENSMUST00000025751** | **Ighmbp2** | **protein_coding** | **0.000450208** | **0.012080281** | **1799** | **10** | **60** | **0.166666667** | **mmu-miR-7093-5p,mmu-miR-1249-5p,mmu-miR-6960-5p,mmu-miR-8104,mmu-miR-6916-5p,mmu-miR-6965-5p,**  **mmu-miR-7047-5p,mmu-miR-6967-5p,mmu-miR-6995-5p,mmu-miR-881-3p** |
| **mmu_circRNA_21040** | **ENSMUST00000080036** | **Htt** | **protein_coding** | **0.000328765** | **0.010019313** | **1575** | **12** | **80** | **0.15** | **mmu-miR-1249-5p,mmu-miR-705,mmu-miR-182-3p,mmu-miR-6965-5p,mmu-miR-7047-5p,mmu-miR-145a-3p**  **,mmu-miR-6967-5p,mmu-miR-6922-5p,mmu-miR-6993-5p,mmu-miR-7008-5p,mmu-miR-6935-5p,mmu-miR-6950-5p** |
| **mmu_circRNA_21040** | **ENSMUST00000032093** | **Prickle2** | **protein_coding** | **0.00030121** | **0.009423213** | **1543** | **11** | **68** | **0.161764706** | **mmu-miR-1249-5p,mmu-miR-7084-3p,mmu-miR-7009-5p,mmu-miR-6975-3p,mmu-miR-148a-3p,mmu-miR-6965-5p,**  **mmu-miR-7047-5p,mmu-miR-6967-5p,mmu-miR-6922-5p,mmu-miR-7008-5p,mmu-miR-136-5p** |
| **mmu_circRNA_21040** | **ENSMUST00000141387** | **Sypl2** | **protein_coding** | **0.000300658** | **0.009423213** | **1536** | **13** | **91** | **0.142857143** | **mmu-miR-1249-5p,mmu-miR-7073-5p,mmu-miR-29a-3p,mmu-miR-8113,mmu-miR-6337,mmu-miR-6916-5p,**  **mmu-miR-7043-3p,mmu-miR-5107-5p,mmu-miR-6965-5p,mmu-miR-29b-3p,mmu-miR-7047-5p,mmu-miR-6904-5p,**  **mmu-miR-29c-3p** |
| **mmu_circRNA_21040** | **ENSMUST00000054607** | **Ahcy** | **protein_coding** | **0.000199121** | **0.007265293** | **1320** | **12** | **76** | **0.157894737** | **mmu-miR-1249-5p,mmu-miR-6337,mmu-miR-6975-3p,mmu-miR-5627-3p,mmu-miR-6965-5p,mmu-miR-7047-5p,**  **mmu-miR-6967-5p,mmu-miR-6922-5p,mmu-miR-6993-5p,mmu-miR-7229-3p,mmu-miR-7033-5p,mmu-miR-3082-5p** |
| **mmu_circRNA_21040** | **ENSMUST00000103027** | **Mgat5b** | **protein_coding** | **6.47E-05** | **0.003578994** | **870** | **12** | **68** | **0.176470588** | **mmu-miR-1249-5p,mmu-miR-705,mmu-miR-7042-3p,mmu-miR-6965-5p,mmu-miR-7047-5p,mmu-miR-6967-5p,**  **mmu-miR-3099-5p,mmu-miR-6993-5p,mmu-miR-7008-5p,mmu-miR-6935-5p,mmu-miR-6945-5p,mmu-miR-6954-5p** |
| **mmu_circRNA_21040** | **ENSMUST00000194598** | **Syngap1** | **protein_coding** | **6.14E-05** | **0.00345493** | **855** | **15** | **101** | **0.148514851** | **mmu-miR-1249-5p,mmu-miR-705,mmu-miR-5107-5p,mmu-miR-6965-5p,mmu-miR-328-5p,mmu-miR-7047-5p,**  **mmu-miR-6967-5p,mmu-miR-7239-5p,mmu-miR-6993-5p,mmu-miR-6995-5p,mmu-miR-207,mmu-miR-6945-5p**  **,mmu-miR-6356,mmu-miR-3110-3p,mmu-miR-6954-5p** |
| **mmu_circRNA_21040** | **ENSMUST00000063663** | **B3gat2** | **protein_coding** | **4.76E-05** | **0.00296046** | **771** | **11** | **56** | **0.196428571** | **mmu-miR-1249-5p,mmu-miR-705,mmu-miR-6965-5p,mmu-miR-7047-5p,mmu-miR-6967-5p,mmu-miR-6922-5p,**  **mmu-miR-3099-5p,mmu-miR-6993-5p,mmu-miR-7008-5p,mmu-miR-6935-5p,mmu-miR-3082-5p** |
| **mmu_circRNA_21040** | **ENSMUST00000067444** | **Gfap** | **protein_coding** | **4.76E-05** | **0.00296046** | **774** | **11** | **56** | **0.196428571** | **mmu-miR-7093-5p,mmu-miR-1249-5p,mmu-miR-6960-5p,mmu-miR-8104,mmu-miR-705,mmu-miR-6975-3p,**  **mmu-miR-7042-3p,mmu-miR-6965-5p,mmu-miR-7047-5p,mmu-miR-6967-5p,mmu-miR-6950-5p** |
| **mmu_circRNA_21040** | **ENSMUST00000017576** | **Rbfox3** | **protein_coding** | **4.44E-05** | **0.002795821** | **765** | **14** | **87** | **0.16091954** | **mmu-miR-7087-3p,mmu-miR-1249-5p,mmu-miR-3474,mmu-miR-6337,mmu-miR-7009-5p,mmu-miR-3552,**  **mmu-miR-6961-5p,mmu-miR-5627-3p,mmu-miR-6965-5p,mmu-miR-7047-5p,mmu-miR-6904-5p,mmu-miR-6914-5p**  **,mmu-miR-6945-5p,mmu-miR-6954-5p** |
| **mmu_circRNA_21040** | **ENSMUST00000028794** | **Siglec1** | **protein_coding** | **3.50E-05** | **0.002395518** | **704** | **10** | **45** | **0.222222222** | **mmu-miR-1249-5p,mmu-miR-7073-5p,mmu-miR-10b-5p,mmu-miR-10a-5p,mmu-miR-6965-5p,mmu-miR-7047-5p,**  **mmu-miR-6967-5p,mmu-miR-6950-3p,mmu-miR-7033-5p,mmu-miR-6950-5p** |
| **mmu_circRNA_21040** | **ENSMUST00000040280** | **Slc25a23** | **protein_coding** | **2.90E-05** | **0.002090159** | **660** | **15** | **95** | **0.157894737** | **mmu-miR-1249-5p,mmu-miR-7073-5p,mmu-miR-3474,mmu-miR-7009-5p,mmu-miR-705,mmu-miR-7042-3p,**  **mmu-miR-6965-5p,mmu-miR-7047-5p,mmu-miR-6967-5p,mmu-miR-6922-5p,mmu-miR-6935-5p,mmu-miR-6914-5p,**  **mmu-miR-6945-5p,mmu-miR-6954-5p,mmu-miR-6950-5p** |
| **mmu_circRNA_21040** | **ENSMUST00000047889** | **Atp1b2** | **protein_coding** | **2.90E-05** | **0.002090159** | **663** | **15** | **95** | **0.157894737** | **mmu-miR-7681-3p,mmu-miR-1249-5p,mmu-miR-7073-5p,mmu-miR-3474,mmu-miR-10b-5p,mmu-miR-10a-5p,**  **mmu-miR-7009-5p,mmu-miR-6961-5p,mmu-miR-5627-3p,mmu-miR-6965-5p,mmu-miR-7047-5p,mmu-miR-7680-5p,**  **mmu-miR-6914-5p,mmu-miR-6945-5p,mmu-miR-3082-5p** |
| **mmu_circRNA_21040** | **ENSMUST00000095873** | **Kcnj6** | **protein_coding** | **2.40E-05** | **0.00189984** | **609** | **13** | **72** | **0.180555556** | **mmu-miR-7093-5p,mmu-miR-1249-5p,mmu-miR-6960-5p,mmu-miR-3474,mmu-miR-8104,mmu-miR-6916-5p,**  **mmu-miR-5627-3p,mmu-miR-6965-5p,mmu-miR-328-5p,mmu-miR-7047-5p,mmu-miR-1941-5p,mmu-miR-6967-5p,**  **mmu-miR-6995-5p** |
| **mmu_circRNA_21040** | **ENSMUST00000168786** | **Iqsec2** | **protein_coding** | **1.91E-05** | **0.001606957** | **573** | **14** | **81** | **0.172839506** | **mmu-miR-1249-5p,mmu-miR-8113,mmu-miR-6337,mmu-miR-1931,mmu-miR-337-3p,mmu-miR-6916-5p,**  **mmu-miR-6975-3p,mmu-miR-3552,mmu-miR-5627-3p,mmu-miR-6965-5p,mmu-miR-328-5p,mmu-miR-7215-3p**  **,mmu-miR-7047-5p,mmu-miR-7033-5p** |
| **mmu_circRNA_21040** | **ENSMUST00000018710** | **Slc2a4** | **protein_coding** | **1.52E-05** | **0.001396932** | **525** | **11** | **50** | **0.22** | **mmu-miR-1249-5p,mmu-miR-3108-3p,mmu-miR-10b-5p,mmu-miR-10a-5p,mmu-miR-5107-5p,mmu-miR-5627-3p,**  **mmu-miR-6965-5p,mmu-miR-7047-5p,mmu-miR-3109-3p,mmu-miR-7008-5p,mmu-miR-6950-5p** |
| **mmu_circRNA_21040** | **ENSMUST00000006112** | **Ephb3** | **protein_coding** | **1.24E-05** | **0.00122163** | **489** | **18** | **123** | **0.146341463** | **mmu-miR-7087-3p,mmu-miR-6955-5p,mmu-miR-1249-5p,mmu-miR-3474,mmu-miR-146a-5p,mmu-miR-6961-5p,**  **mmu-miR-5627-3p,mmu-miR-6965-5p,mmu-miR-328-5p,mmu-miR-7215-3p,mmu-miR-7047-5p,mmu-miR-3109-3p,**  **mmu-miR-6904-5p,mmu-miR-6914-5p,mmu-miR-5114,mmu-miR-6945-5p,mmu-miR-6356,mmu-miR-146b-5p** |
| **mmu_circRNA_21040** | **ENSMUST00000007961** | **Zmiz1** | **protein_coding** | **1.10E-05** | **0.001129692** | **472** | **15** | **88** | **0.170454545** | **mmu-miR-1249-5p,mmu-miR-3474,mmu-miR-6337,mmu-miR-6916-5p,mmu-miR-705,mmu-miR-3552,mmu-miR-6965-5p,**  **mmu-miR-7047-5p,mmu-miR-6967-5p,mmu-miR-6922-5p,mmu-miR-6935-5p,mmu-miR-6904-5p,mmu-miR-6914-5p**  **,mmu-miR-5114,mmu-miR-6954-5p** |
| **mmu_circRNA_21040** | **ENSMUST00000159068** | **Adra1a** | **protein_coding** | **4.21E-06** | **0.000604895** | **336** | **13** | **62** | **0.209677419** | **mmu-miR-1249-5p,mmu-miR-7073-5p,mmu-miR-705,mmu-miR-3552,mmu-miR-7061-3p,mmu-miR-6965-5p,**  **mmu-miR-7047-5p,mmu-miR-6967-5p,mmu-miR-6922-5p,mmu-miR-7239-5p,mmu-miR-6993-5p,mmu-miR-7008-5p,**  **mmu-miR-6950-3p** |
| **mmu_circRNA_21040** | **ENSMUST00000066778** | **Pi4k2a** | **protein_coding** | **3.15E-06** | **0.000509044** | **297** | **14** | **70** | **0.2** | **mmu-miR-7681-3p,mmu-miR-7087-3p,mmu-miR-1249-5p,mmu-miR-7073-5p,mmu-miR-877-5p,mmu-miR-5107-5p,**  **mmu-miR-6965-5p,mmu-miR-7215-3p,mmu-miR-7047-5p,mmu-miR-6967-5p,mmu-miR-7008-3p,mmu-miR-7033-5p,**  **mmu-miR-3110-3p,mmu-miR-6954-5p** |
| **mmu_circRNA_21040** | **ENSMUST00000079244** | **Mink1** | **protein_coding** | **1.52E-06** | **0.000321346** | **228** | **13** | **57** | **0.228070175** | **mmu-miR-1249-5p,mmu-miR-7073-5p,mmu-miR-7009-5p,mmu-miR-705,mmu-miR-6975-3p,mmu-miR-6965-5p,**  **mmu-miR-7047-5p,mmu-miR-6967-5p,mmu-miR-6922-5p,mmu-miR-3109-3p,mmu-miR-6993-5p,mmu-miR-7008-5p,**  **mmu-miR-6935-5p** |
| **mmu_circRNA_21040** | **ENSMUST00000032451** | **Slc6a11** | **protein_coding** | **2.25E-07** | **9.63E-05** | **113** | **15** | **66** | **0.227272727** | **mmu-miR-1249-5p,mmu-miR-705,mmu-miR-5107-5p,mmu-miR-6965-5p,mmu-miR-328-5p,mmu-miR-7047-5p,**  **mmu-miR-1941-5p,mmu-miR-6922-5p,mmu-miR-6993-5p,mmu-miR-7008-5p,mmu-miR-6950-3p,mmu-miR-6995-5p,**  **mmu-miR-6935-5p,mmu-miR-146b-3p,mmu-miR-7035-3p** |

# Supplementary figures

**2.1 supplementary figures of the whole images (original blot) for western blotting in hippocampal synaptic plasticity.**

**2.1.1 The representative orginal blot of Arc and internal reference protein Gapdh. The lanes showed in every figure are STD-1，STD-2, STD-3, HFD-1, HFD-2, HFD-3 from left to right.**


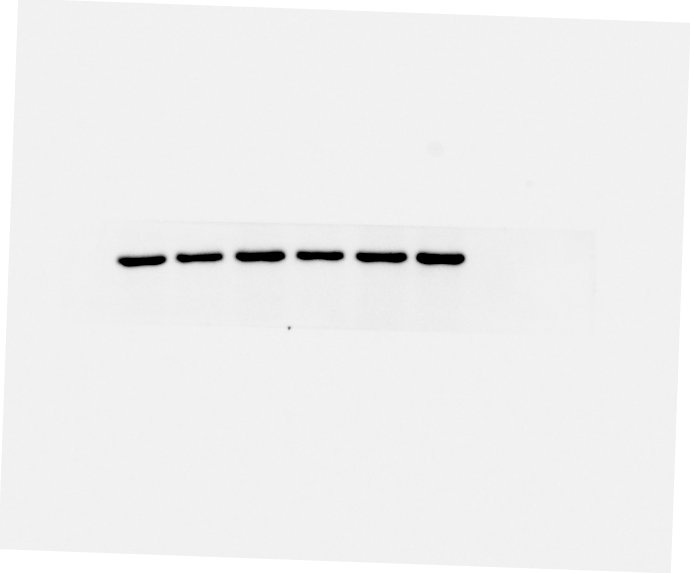

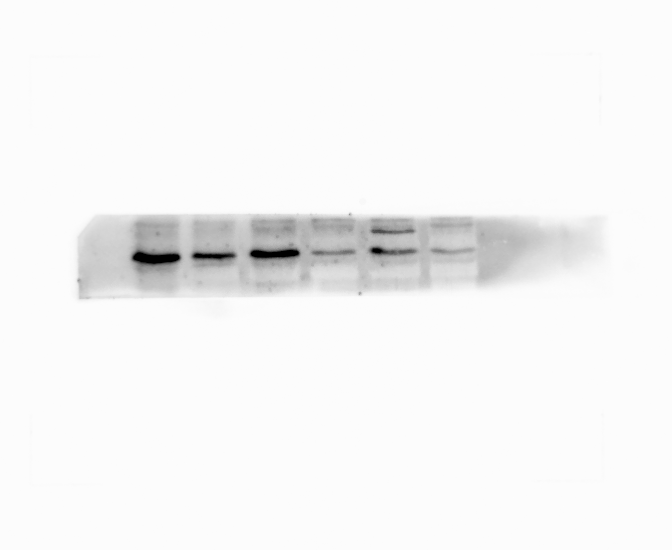


Gapdh

Arc

### 2.1.2 The **representative** orginal blot of psd-95, Syn and internal reference protein Tubulin. The lanes showed in every figure are STD-1，STD-2, STD-3, HFD-1, HFD-2, HFD-3 from left to right.


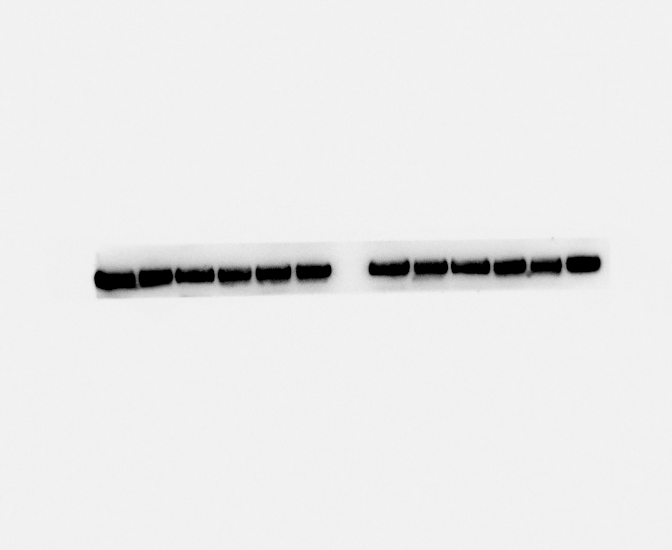

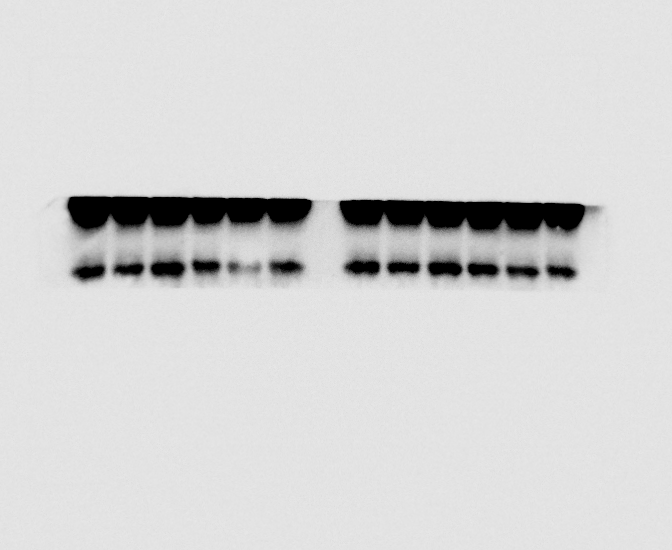

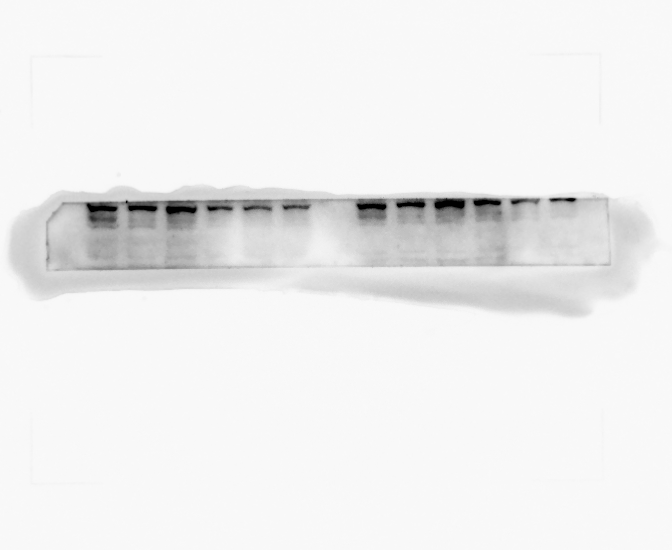


Tubulin

Syn

PSD-95

**2.2 supplementary figures of the whole images (original blot) for western blotting in ER stress.**

**2.2.1 The representative orginal blot of GPR78 and internal reference protein Actin. The lanes showed in every figure are STD-1，STD-2, STD-3, HFD-1, HFD-2, HFD-3 from left to right.**

**
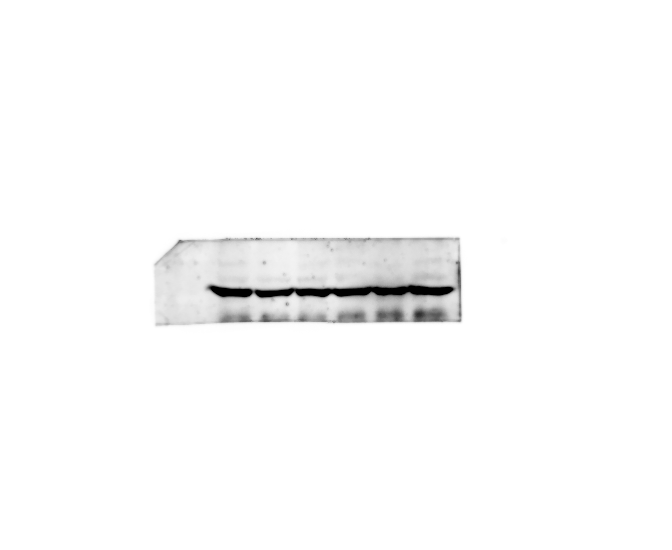

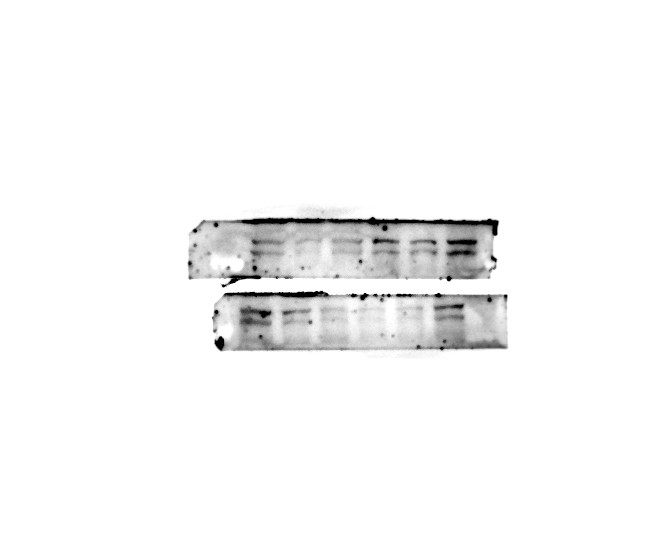
**

GPR78

Actin

**2.2.2 The representative orginal blot of CHOP and internal reference protein Actin. The lanes showed in every figure are STD-1，STD-2, STD-3, HFD-1, HFD-2, HFD-3 from left to right.**


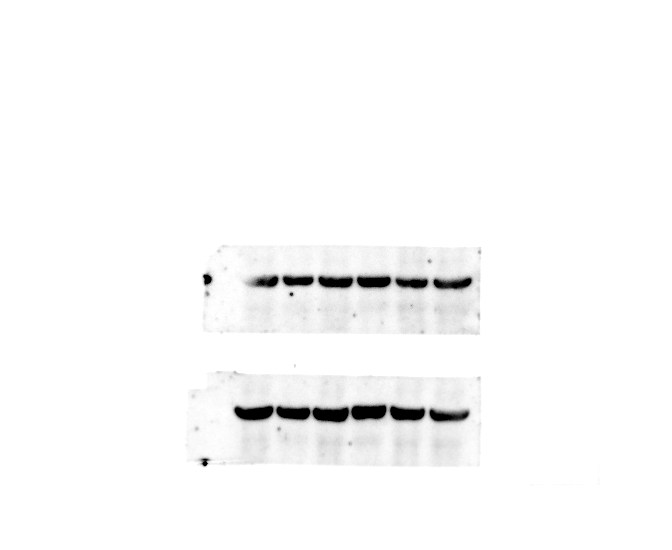

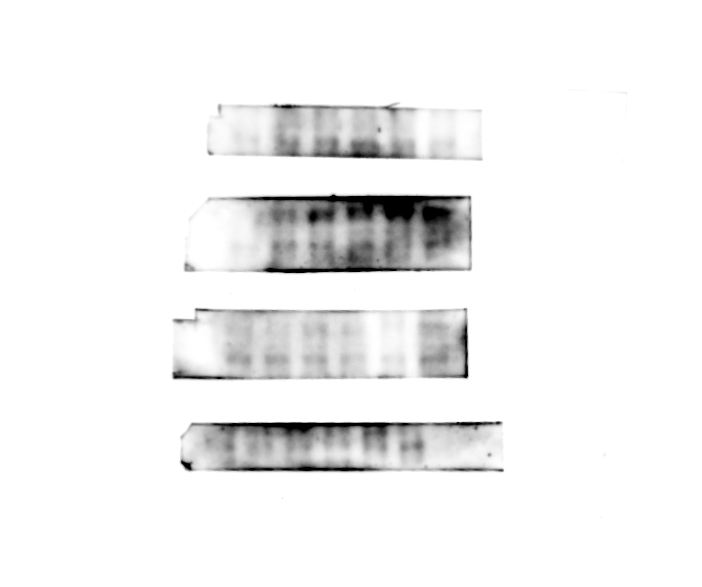


CHOP

Actin

**2.2.3 The representative orginal blot of eIF2α，p-eIF2α and internal reference protein Tubulin.The lanes showed in every figure are STD-1，STD-2, STD-3, HFD-1, HFD-2, HFD-3 from left to right.**


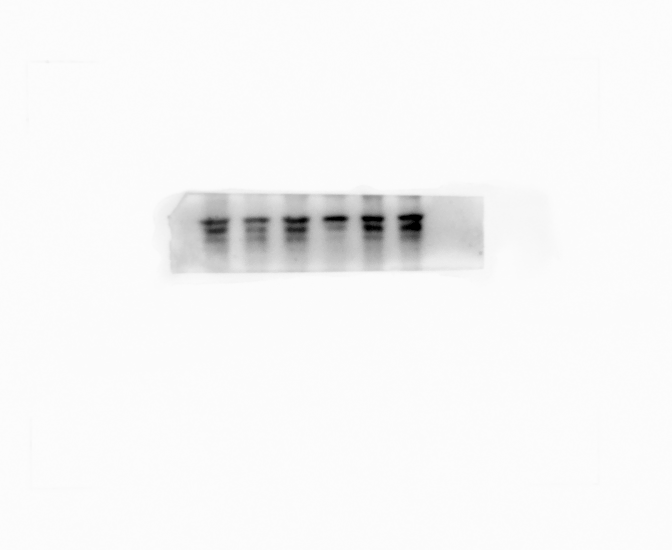

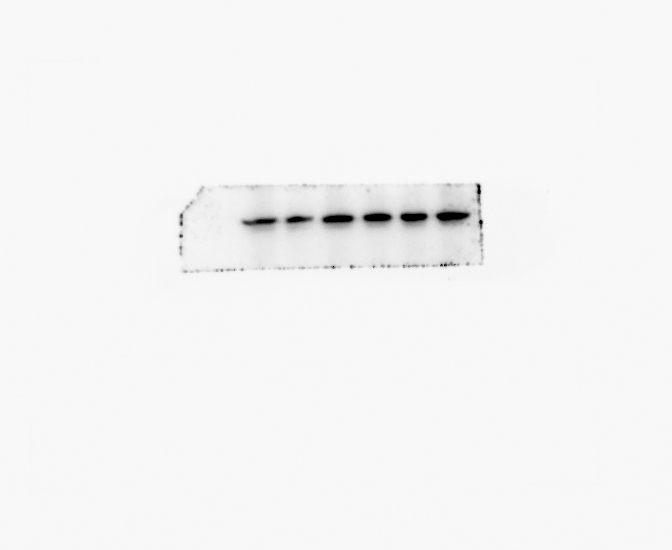

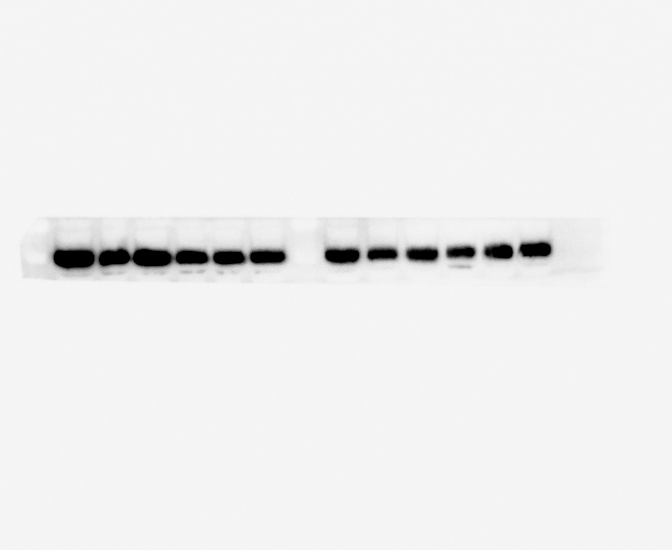


**p-eIF2α**

**Tubulin**

**eIF2α**

**2.2.4 The representative orginal blot of PERK，p-PERK and internal reference protein Gapdh.The lanes showed in every figure are STD-1，STD-2, STD-3, HFD-1, HFD-2, HFD-3 from left to right.**


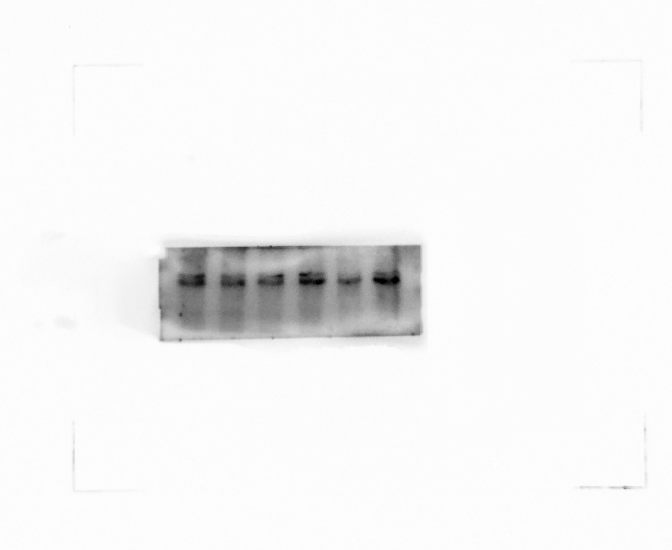

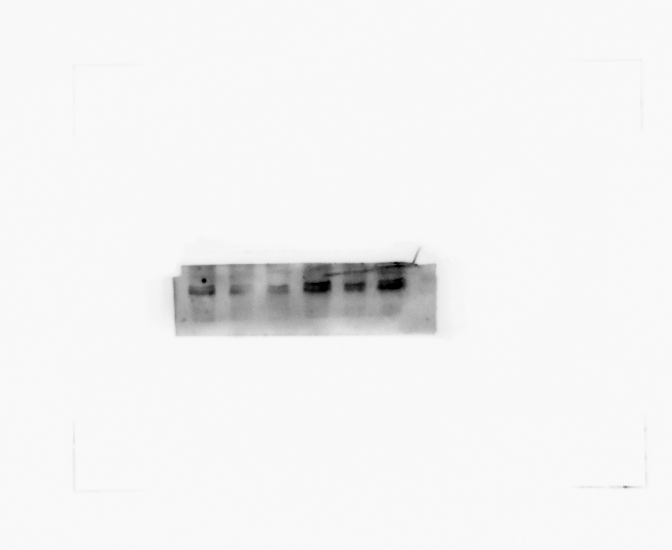

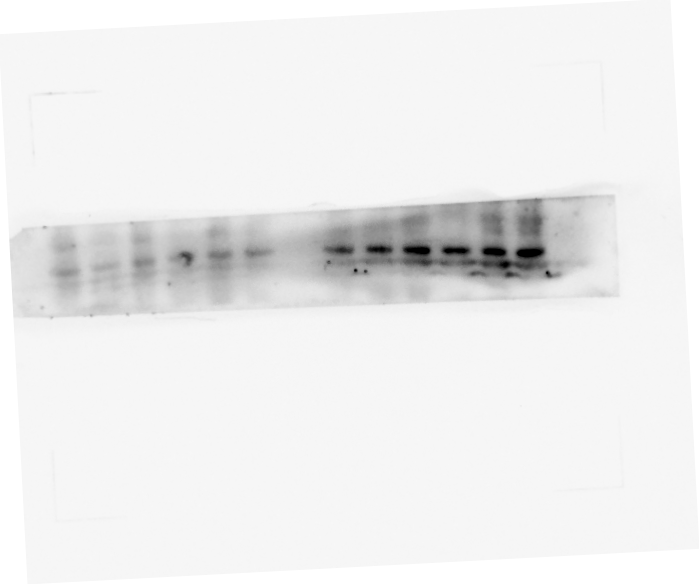


PERK

Gapdh

P-PERK
